# Programmable supramolecular chirality in non-equilibrium systems affording a multistate chiroptical switch

**DOI:** 10.1038/s41467-023-40698-9

**Published:** 2023-08-18

**Authors:** Jingjing Li, Yihan Cui, Yi-Lin Lu, Yunfei Zhang, Kaihuang Zhang, Chaonan Gu, Kaifang Wang, Yujia Liang, Chun-Sen Liu

**Affiliations:** 1 School of Chemistry and Chemical Engineering, Henan University of Technology, Zhengzhou, 450001 China; 2 College of New Energy, Zhengzhou University of Light Industry, Zhengzhou, 450002 China; 3 Department of Chemistry, Key Laboratory of Advanced Energy Materials Chemistry (Ministry of Education), Nankai University, Tianjin, 300071 China

**Keywords:** Self-assembly, Gels and hydrogels, Gels and hydrogels

## Abstract

The dynamic regulation of supramolecular chirality in non-equilibrium systems can provide valuable insights into molecular self-assembly in living systems. Herein, we demonstrate the use of chemical fuels for regulating self-assembly pathway, which thereby controls the supramolecular chirality of assembly in non-equilibrium systems. Depending on the nature of different fuel acids, the system shows pathway-dependent non-equilibrium self-assembly, resulting in either dynamic self-assembly with transient supramolecular chirality or kinetically trapped self-assembly with inverse supramolecular chirality. More importantly, successive conducting of chemical-fueled process and thermal annealing process allows for the sequential programmability of the supramolecular chirality between four different chiral hydrogels, affording a new example of a multistate supramolecular chiroptical switch that can be recycled multiple times. The current finding sheds new light on the design of future supramolecular chiral materials, offering access to alternative self-assembly pathways and kinetically controlled non-equilibrium states.

## Abstract (web summary)

The dynamic regulation of supramolecular chirality in non-equilibrium systems can provide valuable insights into molecular self-assembly in living systems. Here, the authors demonstrate the use of chemical fuels for regulating self-assembly pathway, which thereby controls the supramolecular chirality of a non-equilibrium assembled system.

## Introduction

Supramolecular chirality, resulting from asymmetric assembly of chiral or achiral molecules, has been extensively studied due to the crucial roles it plays in natural biological systems and in advanced materials^1–7^. Various external stimuli such as solvent, light, temperature, pH, and achiral additives have been exploited to date to regulate the structure and chirality of the supramolecular assemblies^2,8–13^. Despite huge progress in this field, currently developed supramolecular materials mainly exist in equilibrium with their environment^14^ and remain in sharp contrast with biological non-equilibrium supramolecular structures, in particular, in terms of their autonomy and adaptivity^15^.

Living biological materials often exist far from equilibrium with their surrounding environment, involving continuous energy dissipation, kinetic control, and complex orchestration through feedback loops^15^. Inspired by biological aspects, molecular assemblies coupled with chemical fuel-driven reaction networks have been recently developed to drive the synthetic self-assembled systems far from equilibrium^16–21^. Nonetheless, examples of such systems most commonly focus on tailoring the lifetime or mechanical properties of transient supramolecular assemblies. The few examples involving supramolecular chirality regulation under a non-equilibrium state have been fueled by enzymes working in a complex tandem manner^22–25^. It thus remains a significant challenge in fully synthetic non-equilibrium systems to achieve programmable supramolecular chirality in a controlled and reproducible manner resembling that in a living system.

Guanosine (G) and its derivatives have been widely used in supramolecular self-assembly chemistry for the development of hydrogels, ionophores, catalysts, and in biological applications^26–34^. In particular, guanosine–boron hydrogels have been recently developed as an attractive platform to study self-assembly systems and to create innovative materials^28,31,32,35–37^. Notably, the structure and functions of the bulk materials are often dictated by the molecular design of the building blocks in thermodynamic equilibrium. The guanosine moieties promote extensive self-assembly via the formation of highly ordered G-quadruplex structures^32,36^. The boronic acid moieties introduce new attractive functions such as anticancer and antibacterial activity, and luminescence response^28,31,38^. Nonetheless, recent kinetic studies on self-assembling systems have clearly demonstrated that the properties of supramolecular materials can also depend on the selected preparation pathway^39,40^. The assemblies can remain trapped in a local minimum and the intended thermodynamic minimum is sometimes not reached^41,42^. In general, kinetically trapped structures are often observed for assemblies in, or close to, equilibrium via precise control of heating/cooling steps and optimization of solvent processing^39,40,43^. Most recently, research attempts have been made to use chemical fuels to achieve kinetic control and to selectively reach or avoid the kinetically trapped assemblies^44–48^. For example, Hermans and co-workers demonstrated the use of two redox agents as chemical fuels to drive assembly and disassembly cycles of a perylenediimide derivative in water, allowing the system to circumvent strong kinetic trapping by overcoming assembly barriers^49^. Boekhoven and co-workers demonstrated the pathway dependence in carbodiimide fuel-driven dissipative self-assembly, and kinetically trapped assemblies could be successfully achieved by changing the amount of fuel added^46,47^. Despite these advances, it remains elusive to program the molecular self-assembly pathway and thus the supramolecular chirality of the bulk materials. Previously, we developed a boronic ester-based chemical reaction network using KOH and lactone as chemical fuels to drive assembly and disassembly cycles of G and a mono-benzoxaborole (MB) derivative^50^. Due to weak interactions between G and MB, only a transient *P*-helical supramolecular gel was obtained under the stimuli of chemical fuels.

**Fig. 1:**
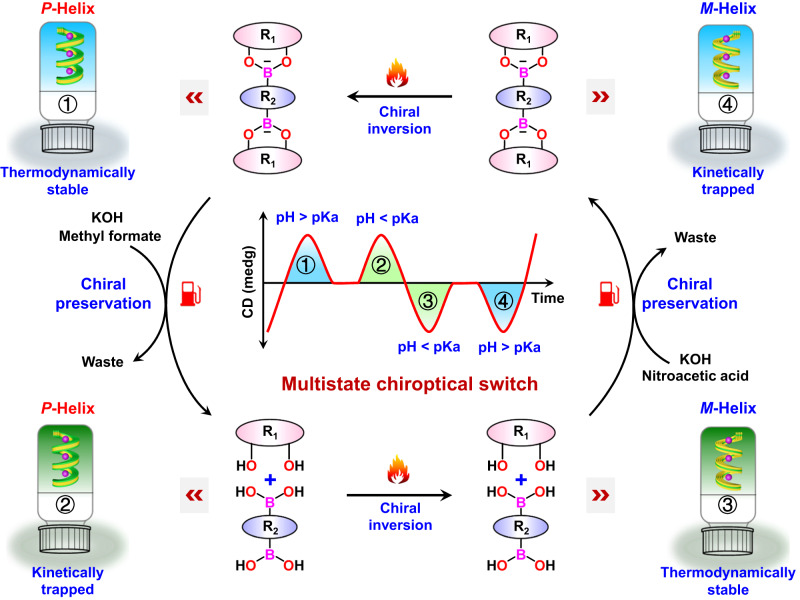
Illustration of the design concept of a multistate supramolecular chiroptical switch. The multistate supramolecular chiroptical switch is achieved via coupling of successive chemical-fueled process and thermal annealing process by using guanosine and a bis-benzoxaborole derivative as precursors. Chemical processes retain the supramolecular chirality, whereas thermal processes reverse the supramolecular chirality.

In this work, we demonstrate the use of chemical fuels to regulate the self-assembly pathway of G and a bis-benzoxaborole derivative and thereby program the supramolecular chirality in non-equilibrium systems. This enables access to a rare example of a multistate supramolecular chiroptical switch that can be recycled multiple times (Fig. 1). The nature of the chemical fuels dictates the self-assembly pathway of supramolecules, resulting in either dissipative self-assembly with transient *P*-helical supramolecular chirality or kinetically trapped self-assembly with inverse *M*- and *P*-helical supramolecular chirality. Exposure to subsequent heating–cooling cyclic process led to the transformation of the kinetically trapped hydrogels into their thermodynamically favored state with opposite helicity. Consequently, an unusual example of a multistate supramolecular chiroptical switch formed by four different chiral hydrogels is achieved (Fig. 1). The entire switching process is found to be repeatable over multiple cycles, demonstrating sequential programmability.

## Results

### Thermally triggered self-assembly and gelation (thermogels)

**Fig. 2:**
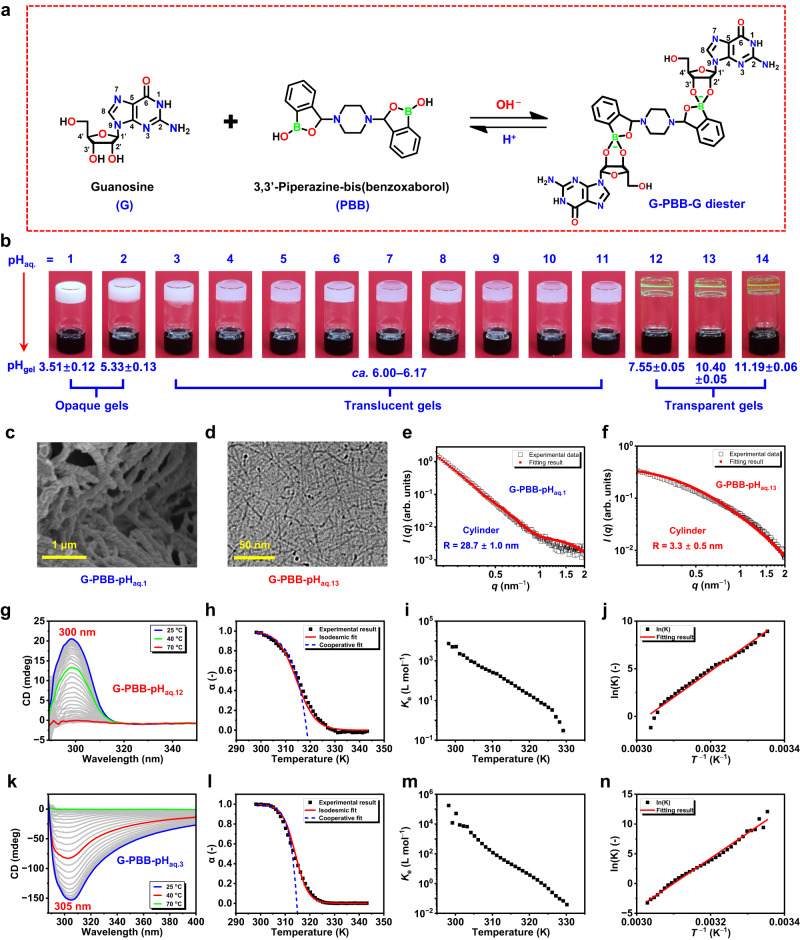
Thermally triggered self-assembly and gelation. **a** Chemical structures of G, PBB, and their reversible reaction under pH stimuli. **b** Optical images of G and PBB mixtures at their CGCs in aqueous K^+^. **c** Cryo-SEM image of G-PBB-pH_aq.1_ hydrogel. **d** Cryo-TEM image of G-PBB-pH_aq.13_ hydrogel. **e**, **f** SAXS analysis of the hydrogels. **g, k** Temperature-dependent CD spectra of G-PBB hydrogels at 1 °C intervals. **h**, **l** Temperature-dependent degree of aggregation, α, calculated from the CD signals at 300 and 305 nm in graphs (g) and (k), respectively. Lines are the corresponding isodesmic fit (red solid line) and cooperative fit (blue dashed line). **i, m** elongation equilibrium constant, *K*_e_, as a function of temperature. **j**, **n** Van’t Hoff plot. Graphs (**g**–**j**) are for G-PBB-pH_aq.12_ hydrogel and graphs (**k**–**n**) are for G-PBB-pH_aq.3_ hydrogel. In all graphs, hydrogels were prepared at their CGCs and molar ratio of G:PBB = 2:1.

First, the conventional thermal annealing approach was followed to investigate the pH-dependent gelation properties and self-assembly mechanisms of G and 3,3’-piperazine-bis(benzoxaborol) (PBB) mixture under equilibrium conditions (Fig. 2a). Details of synthesis and characterization of PBB are presented in Supplementary Figs. 1–3. After a simple heating–cooling cycle, mixture of G and PBB (ratio 2:1) exhibited exceptional gelation capacity in aqueous K^+^ within a broad range of pH (1–14) (Fig. 2b). These gels were prepared via conventional heating–cooling method are defined as G-PBB-pH_aq.n_ thermogels, where n = 1–14 represents the pH of the aqueous solution used to prepare the gels. Detailed characteristics of the gelation properties of these hydrogels are presented in Supplementary Table 2, Supplementary Figs. 7 and 8. In particular, transparent hydrogel with obvious Tyndall effect was achieved at critical gelator concentration (CGC) of 1.5% w/v of G in aqueous solution with pH 13; however, the translucent hydrogel was observed at 0.5% w/v of G in aqueous solution with pH 7 and opaque hydrogel was observed at 4.0% w/v of G in aqueous solution with pH 1 (Fig. 2b). In these systems, the final pH of the gel was affected by the presence of PBB. The actual pH values of G-PBB-pH_aq.1_, G-PBB-pH_aq.7_, and G-PBB-pH_aq.13_ hydrogels at their CGCs were 3.51 ± 0.12, 6.07 ± 0.09, and 10.40 ± 0.05, respectively (Fig. 2b, Supplementary Table 2), clearly showing a broad pH window crossing the pKa of PBB itself (7.44 ± 0.01, Supplementary Fig. 9). Control experiments showed that, without addition of G or PBB, or replacing KOH with LiOH, no hydrogels could be obtained in pH 1–14 aqueous. These findings highlight the significant role of G, PBB, and K^+^ as well as their strong interactions for gelation (Supplementary Fig. 10).

Insights into gel morphology were obtained from cryo-scanning electron microscopy (SEM), cryo-transmission electron microscopy (TEM), and atomic force microscopy (AFM) (Fig. 2c, d). Interestingly, cryo-SEM of the acidic G-PBB-pH_aq.1_ hydrogel displayed left-handed helical fiber structures with a diameter of approximately 150 nm (Fig. 2c and Supplementary Fig. 11a). However, due to low resolution, cryo-SEM of the alkaline G-PBB-pH_aq.13_ hydrogel did not show discernable fiber structures (Supplementary Fig. 11b). Cryo-TEM revealed that G-PBB-pH_aq.13_ hydrogel produced uniform fibers with a diameter of ~3 nm (Fig. 2d and Supplementary Fig. 11c), agreeing well with its high transparency. However, the fibers were too thin to discern their macromolecular helicity. AFM height profiles of a single nanofiber of these gels showed similar morphology and thickness to that of the cryo-SEM/TEM observations (Supplementary Fig. 12). The small-angle X-ray scattering (SAXS) data for these hydrogels (Fig. 2e, f) fit well to a cylindrical model with a radius of about 28.7 ± 1.0 nm and 3.3 ± 0.5 nm for G-PBB-pH_aq.1_ and G-PBB-pH_aq.13_ hydrogels, respectively. The circular dichroism (CD) spectrum of G-PBB-pH_aq.1_ hydrogel displayed a strong negative CD signal at 310 nm (Supplementary Fig. 13), indicating a left-handed helical stacking of the supramolecular assemblies^51^, which is consistent with the cryo-SEM observations. In contrast, G-PBB-pH_aq.13_ hydrogel showed a positive CD signal at around 300 nm, generating an opposite CD spectrum compared to G-PBB-pH_aq.1_ hydrogel (Supplementary Fig. 13). CD spectra of G-PBB hydrogels prepared in aqueous solutions with other pH are shown in Supplementary Fig. 13. Linear dichroism effects can be neglected owing to the low signal intensity^39^ (Supplementary Fig. 14) and the corroborating evidence of microscopy images.

The melting curves of two representative G-PBB hydrogels with inverse helicity at the same concentration (0.5% w/v of G) were investigated by temperature-dependent CD spectroscopy to identify the supramolecular polymerization mechanism^52,53^. Figure 2g, k demonstrate that for both hydrogels, upon heating the gel from 298.15 to 343.15 K, the CD signals gradually decreased with increasing temperature and were silent in solution state, which indicated gelation-induced supramolecular chirality^4,29^. For G-PBB-pH_aq.12_ hydrogel, when the fraction of aggregates (α), from the normalized CD curves, was plotted as a function of temperature, the data could be well fitted to the isodesmic model (Fig. 2h)^54^, giving a melting temperature (*T*_m_) of 314.5 K and a binding constant (*K*_e_) of 5.3 × 10^3^ M^−1^ at 300.15 K (Fig. 2i and Supplementary Table 3). The molar enthalpy (∆*H*), determined by using the model, was −242.3 kJ·mol^−1^ (Supplementary Table 3). This ∆*H* value corresponds well with the value of −229.8 kJ·mol^−1^ evaluated from Van’t Hoff plot (Fig. 2j and Supplementary Table 3). The corresponding molar entropy (∆*S*) was determined to be −695.8 J·mol^−1^·K^−1^. The same isodesmic model was also used to fit the data of G-PBB-pH_aq.3_ hydrogel (Fig. 2k–n), giving a series of thermodynamic parameters as presented in Supplementary Table 3. The negative enthalpy and entropy values indicate that the self-assembly is an enthalpy-driven process.

**Fig. 3:**
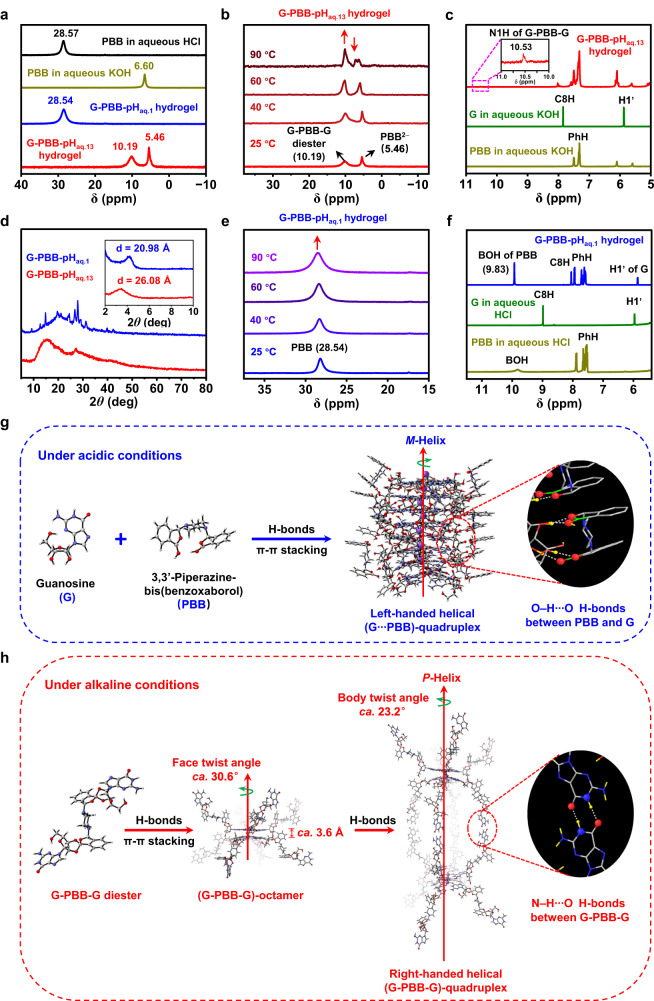
Assembly mechanism of G and PBB mixtures. **a**, **d** ^11^B NMR spectra and XRD patterns. **b**, **e** Variable-temperature ^11^B NMR spectra of G-PBB-pH_aq.13_ and G-PBB-pH_aq.1_ hydrogels. **c**, **f** ^1^H NMR spectra of free G, PBB and G-PBB hydrogels under both acidic and alkaline conditions. **g**, **h** Assembly mechanism of G and PBB mixtures under acidic (**g**) and alkaline (**h**) conditions (Supplementary Movie 1). Atoms are colored as follows: red - oxygen, blue - nitrogen, gray - carbon, pink - boron, white - hydrogen, and purple - potassium.

In order to reveal the chemical component responsible for gelation in both acidic and alkaline conditions, different spectroscopy tests were performed (Fig. 3). For alkaline hydrogels, considering G-PBB-pH_aq.13_ hydrogel as a representative example, ^11^B NMR spectra showed that the boron signal of free PBB molecules in aqueous HCl with pH 1 corresponded to a low-field signal at 28.57 ppm, which shifted to a high-field signal at 6.60 ppm after deprotonation with KOH (pH = 13) (Fig. 3a). This result indicates the change in hybridization from the acidic trigonal configuration (sp^2^) to the alkaline tetrahedral configuration (sp^3^)^55^. These results reveal the formation of anionic (bis)benzoxaborolate species (PBB^2−^) in aqueous KOH (pH = 13)^55^. Further addition of G in PBB/KOH aqueous solution resulted in hydrogelation, which caused the shift of the PBB^2−^ signal to 5.46 ppm along with the appearance of a new boron signal at 10.19 ppm (Fig. 3a). This new peak was assigned to the G-PBB-G (bis)benzoxaborolate complex formed via the formation of dynamic boronic ester bonds between *cis*-diol of G and deprotonated PBB^29,50^. This result was further confirmed by ^1^H NMR spectra, which showed a new resonance peak at 10.53 ppm in the spectrum of G-PBB-pH_aq.13_ hydrogel (Fig. 3c), attributed to N1H of G-PBB-G complex^50,56^. The N1H signal of free G in aqueous KOH was not observed probably due to the rapid proton exchange^56^. Accordingly, the C8H and ribose C1ˊH signals of G also shifted downfield due to the formation of G-PBB-G complex (Fig. 3c)^50,56^. Electrospray ionization mass spectrometry (ESI-MS) analysis confirmed the presence of species with m/z = 878.28, corresponding to G-PBB-G diesters (Supplementary Fig. 15). The importance of G-PBB-G diesters for hydrogelation was further proved by variable-temperature ^11^B NMR (VT-NMR) spectroscopic analysis of G-PBB-pH_aq.13_ hydrogels (Fig. 3b). With the increase in temperature from 25 to 90 °C, the relative intensity of G-PBB-G diesters signal relative to PBB^2−^ monomers (I_10.19_/I_5.46_) increased gradually (Supplementary Fig. 16) due to the dissociation of the G-PBB-G diesters in the gel phase to solution phase during melting of the hydrogel^50^. However, for acidic hydrogels, considering G-PBB-pH_aq.1_ hydrogel as an example, only one signal for neutral PBB monomers was observed at 28.54 ppm (Fig. 3a). Despite the increase in the temperature to 90 °C, no other peaks were observed (Fig. 3e), indicating the absence of covalent interactions between G and PBB molecules in these gels. Similarly, ^1^H NMR spectrum of G-PBB-pH_aq.1_ hydrogel showed the presence of a mixture of G and PBB. The BOH of PBB appeared at 9.83 ppm, and the C8H and ribose C1ˊH of G appeared at 7.95 and 5.76 ppm, respectively (Fig. 3f)^50,56^. No new resonance peak was observed, consolidating the result of ^11^B NMR spectrum. Consequently, it was speculated that G and PBB molecules interacted with each other via non-covalent interactions and co-assembled to form the gel fibers. These non-covalent interactions were quite critical for the long-term stability of G-PBB-pH_aq.1_ hydrogels, as evidently, G alone precipitates rapidly in aqueous solutions with pH 1 (within 10 min only) (Supplementary Fig. 10). It should be noted that although self-assembled molecules are NMR silent, VT-NMR spectra can provide reliable information about the molecular structures in gel systems. As the temperature increases, the self-assembled molecules dissolve due to the melting of the gels, allowing them to be detected by NMR. The above results indicate that covalently bonded G-PBB-G diesters self-assembled and promoted the formation of alkaline G-PBB-pH_aq.13_ hydrogel; however, non-covalently bonded G and PBB mixtures co-assembled to form the acidic G-PBB-pH_aq.1_ hydrogel.

The distinct aggregates of G-PBB-pH_aq.1_ and G-PBB-pH_aq.13_ hydrogels were further revealed by X-ray diffraction (XRD) (Fig. 3d). Small-angle XRD (SAXRD) of G-PBB-pH_aq.1_ xerogel showed a prominent peak at 2*θ* = 4.21° with an interlayer distance of 20.98 Å; however, G-PBB-pH_aq.13_ xerogel displayed a slightly larger interlayer distance of 26.08 Å (2*θ* = 3.38°, Fig. 3d). Most importantly, wide-angle XRD (WAXRD) (Fig. 3d) performed on the G-PBB-pH_aq.1_ xerogels showed many more peaks that were significantly sharper and more intense compared to xerogel of G-PBB-pH_aq.13_, indicating more ordered stacking of the molecules in G-PBB-pH_aq.1_ hydrogel. The detailed characteristics of other G-PBB-pH_aq.n_ hydrogels are shown in Supplementary Figs 17 and 18. Notably, the ^11^B NMR spectra of G-PBB-pH_aq.12_ and G-PBB-pH_aq.14_ hydrogels showed similar signals to those of G-PBB-pH_aq.13_ hydrogel (coexistence of G-PBB-G diester and PBB^2−^ signals), indicating a similar gelation mechanism. The ^11^B NMR spectra of G-PBB-pH_aq.2_ hydrogel were similar to that of the G-PBB-pH_aq.1_ hydrogel, displaying only one signal for neutral PBB monomer, revealing their comparable self-assembly mechanism. However, the^11^B NMR spectra of G-PBB-pH_aq.3-11_ hydrogels exhibited both G-PBB-G diester and PBB signals. Therefore, in these hydrogels, both covalent and non-covalent bonding mechanisms contribute to their self-assembly. However, it is still unclear whether the two species co-assemble to form novel supramolecular structures or undergo orthodontic self-assembly.

The pKa of PBB was found to be 7.44 in water as determined by both spectrophotometric and potentiometric methods (Supplementary Fig. 9). Correlation of these observations indicates that, when the pH of the hydrogel is alkaline (above the pKa, as in the case of G-PBB-pH_aq.12–14_ hydrogels), the boron of PBB gets deprotonated. This deprotonated form of PBB is more prone to react with G and form G-PBB-G species, which undergo self-assembly process and form right-handed helical nanostructures with positive chiroptical properties (Supplementary Fig. 13). However, when the pH was sufficiently low (as in the case of G-PBB-pH_aq.1–2_ hydrogels), all the molecules of PBB got protonated and it was assumed that it co-assembled with G molecules, leading to the formation of left-handed helical nanostructures with negative chiroptical properties (Supplementary Fig. 13).

To reveal the geometric configurations of the supramolecular species formed in G-PBB hydrogels, molecular dynamics (MD) simulations were performed (Fig. 3g, h). A conformational search was first performed on all building blocks, and their track diagrams of MD simulations at all levels are shown in Supplementary Fig 19. The most stable configurations (Supplementary Fig. 20) were then selected to create the supramolecular assemblies in subsequent simulations. For G-PBB-pH_aq.1_ hydrogel, theoretical calculations indicate that four G monomers first organized into a nearly planar G-quartet via hydrogen bonding interactions, which further stacked into G-octamer, G-hexadecamer, and higher ordered G-quadruplex columns via extensive π–π stacking interactions (Fig. 3g). K^+^ contributed to the stabilization of the resulting G-quartet. Around the G-quadruplex columns, one PBB molecule hydrogen bonded to two adjacent G-quartets via multiple O–H ∙ ∙ ∙ O interactions, and many of them helically wound around the long axis of the columns, forming supramolecular (G ∙ ∙ ∙ PBB)-quadruplex nanowires with *M*-helicity (Fig. 3g, Supplementary Movie 1). The further helical intertwining of the (G ∙ ∙ ∙ PBB)-quadruplex nanowires formed left-handed helical fiber bundles as indicated by cryo-SEM and AFM images. For G-PBB-pH_aq.13_ hydrogel, the results demonstrated that four of G-PBB-G monomers also formed a (G-PBB-G)-quartet supermacrocycle (Fig. 3h), in which one of the G-bases in G-PBB-G hydrogen bonded with each other to form the (G-PBB-G)-quartet. The other G-base and PBB moieties in G-PBB-G hung around the (G-PBB-G)-quartet to form a pendant-like structure. Head-to-head π–π stacking of two (G-PBB-G)-quartets led to the formation of an octamer that was symmetrical up and down. Further stacking of the octamers via multiple N–H ∙ ∙ ∙ O hydrogen bonding between the residue G moieties led to the formation of a lantern-like hexadecamer referred to as (G-PBB-G)-quadruplex. Such one-dimensional (1D) column stacking of the G–boric diester compounds is in striking contrast to the previously proposed 2D plane-like self-assembly model^32,57,58^. Within an individual octamer, the face–face distance between the two layers of (G-PBB-G)-quartets was around 3.6 Å, and the face twist angle between them was around 30.6°. In contrast, for the hexadecamer, the body–body distance between the two stacked octamers was around 27 Å, and the body twist angle was around 23.2°. Such asymmetric stacking of the supramolecular assemblies results in the formation of the right-handed helical (G-PBB-G)-quadruplex nanowires (Fig. 3h, Supplementary Movie 1). These results clearly demonstrate the different self-assembly mechanisms of G and PBB mixture under acidic and alkaline conditions. The energies of all above-mentioned species are presented in Supplementary Table 4. The strong intermolecular hydrogen bonding and π–π stacking interactions of these species were proven experimentally by fourier transform infrared (FTIR) spectroscopy and UV–vis absorption spectroscopy (Supplementary Figs. 21, 22, see Supplementary Information for detailed discussion). Additionally, time-dependent density functional theory (TD-DFT) calculations were performed to confirm the simulated molecular packing structures (Supplementary Fig. 23). The results show that the theoretical CD spectra for helically arranged motifs derived from self-assembled G-quadruplex structures are in good agreement with the experimental results. These data consolidate the results of MD simulation.

### Chemical fuels-triggered self-assembly and gelation (chemigels)

**Fig. 4:**
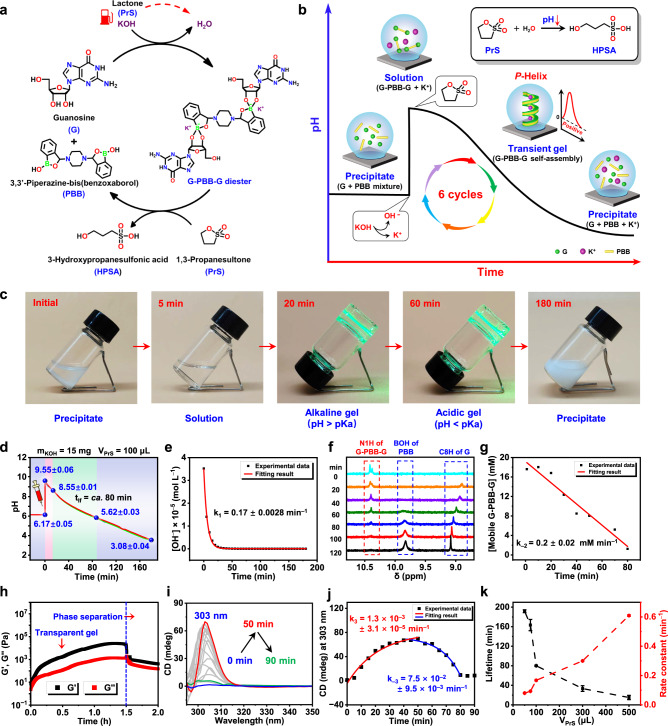
Transient control over the supramolecular chirality and self-assembly kinetics. **a** Schematic illustration of the chemical reaction network fueled with KOH and PrS. **b** Cartoon representing pH evolution profile for the temporal programming of the self-assemblies. **c** Phase transition of G and PBB mixture after cycle initiation (Supplementary Movie 2). **d** pH–time profiles after cycle initiation, three samples were tested in parallel. **e** The concentration of OH^−^ against time during the hydrolysis of PrS. The measured pH is converted to [OH^−^]. **f** Time-evolved ^1^H NMR spectra after cycle initiation. **g** Curve of concentration of mobile G-PBB-G versus time. **h**, **i** Time-dependent rheological moduli profiles (**h**) and CD spectra (**i**) after cycle initiation. **j** CD signal at 303 nm versus time. **k** The plot of rate constant of PrS hydrolysis and lifetime of the transient hydrogels against PrS concentrations.

The inverse chiroptical signal and broad pH sensitivity properties of the G-PBB hydrogels inspired us to develop in situ regulation of the helical structures resembling those in living system^59,60^. Toward this end, this system was further coupled with pH feedback loop to improve the system control. KOH and 1,3-propanesultone (PrS) were first employed as chemical fuels to drive the autonomous formation and dissociation of G-PBB-G diester in a chemical reaction network (Fig. 4a, b). Briefly, KOH (15 mg, 53.5 mM) and PrS (100 μL, 1.9 mM) were added to the mixture of G and PBB (2:1, 1.0% w/v in G, the total volume was 5.0 mL) with an initial pH of 6.17 ± 0.05 (Fig. 4d). This led to a rapid increase in the pH to 9.55 ± 0.06 (Fig. 4d), which chemical conversion of the G and PBB mixtures into their corresponding boronate G-PBB-G (Fig. 4a). Spontaneous hydrolysis of PrS in water yielded 3-hydroxypropanesulfonic acid (HPSA) over time (Fig. 4b), which reduced the pH of the system (Fig. 4d) and in turn led to the decrease in the solubility of G-PBB-G. Consequently, G-PBB-G self-assembled and formed a transient hydrogel with a lifetime of around 80 min across the pH range of 8.55 ± 0.01 to 5.62 ± 0.03 (Fig. 4c, d). The hydrolysis rate of PrS (k_1_) was found to be 0.17 ± 0.0028 min^−1^ based on (pseudo) first-order kinetic fitting (Fig. 4e). Time-dependent rheological test confirmed the formation of the transient hydrogel (Fig. 4h). Further decrease of the pH led to complete dissociation of the G-PBB-G, which in turn disassembled the hydrogel and formed precipitate made of G and PBB mixture (Fig. 4b, c, Supplementary Movie 2). Refueling with KOH and PrS reverted the G and PBB mixture back to G-PBB-G diesters, allowing temporal and repetitive access to gel states for at least six cycles (Supplementary Figs. 24, 25 and Supplementary Table 5). Notably, an important issue with synthetic dissipative systems is the accumulation of waste products, which will poison the system^44,61–63^. By using small molecules as chemical fuels (KOH and PrS), our system maintains the waste at a minimal level. As a result, the system can maintain a stable assembly cycle of at least six rounds.

To link the observed phenomenon to molecular-scale reactions, detailed kinetic analysis was performed. The concentrations of G-PBB-G after addition of chemical fuels were monitored by NMR spectroscopy method (Fig. 4f, g)^64,65^. The results show that G-PBB-G formation (k_2_) is almost instantaneous upon addition of KOH and PrS. The reaction had reached full conversion before the first data point could be measured (data not shown). Over time, G-PBB-G gradually dissociated as evidenced by the gradually decreased characteristic signal at *ca*. 10.4 ppm in the ^1^H NMR spectra (Fig. 4f). Accordingly, the generated PBB (*ca*. 9.8 ppm) and G (*ca*. 8.9 ppm) signals gradually appeared and increased over time (Fig. 4f). Linear fits of the time-dependent dissociation data of G-PBB-G provide the rate constant (k_−2_) of 0.2 ± 0.02 mM·min^−1^ (Fig. 4g). Furthermore, time-evolved CD spectra were employed to monitor the self-assembly and disassembly kinetics of G-PBB-G (Fig. 4i, j). Over the first 50 min, the positive CD signal at 300 nm gradually increased, indicating an increase in chiral ordering with *P*-helix (Fig. 4j). After *ca*. 50 min, the CD signal showed continuous decrease and became almost invisible after *ca*. 90 min (Fig. 4j). Linear fits of these data provide a self-assembly rate constant (k_3_) of 1.3 × 10^−3^ ± 3.1 × 10^−5^ min^−1^, and a disassembly rate constant (k_−3_) of 7.5 × 10^−2^ ± 9.5 × 10^−3^ min^−1^ (Fig. 4j).

The chemical reaction network was driven by chemical fuels; therefore, herein, an attempt was further made to achieve kinetic control of the entire system by changing fuel-conversion kinetics. It was observed that lifetimes of the transient hydrogels could be programmed from *ca*. 15 min to more than 3 h by simply changing the amount of PrS present in the system (Supplementary Fig. 26 and Supplementary Table 6). The PrS hydrolysis kinetics are directly responsible for the observed change in the lifetime of the transient hydrogels (Fig. 4k). An increase in PrS concentration resulted in an accelerated hydrolysis rate, which increased the dissociation rate of G-PBB-G borate and ultimately reduced the lifetime of the transient gels (Fig. 4k, Supplementary Table 6 and Supplementary Fig. 26). As a result, the temporal hydrogel properties thus depend strongly on the kinetics of fuel conversion. Anyway, this system demonstrates the transient accessing of a *P*-helical supramolecular hydrogel with programmable lifetime and controllable formation kinetics.

### Kinetically trapped supramolecular chiral inversion

**Fig. 5:**
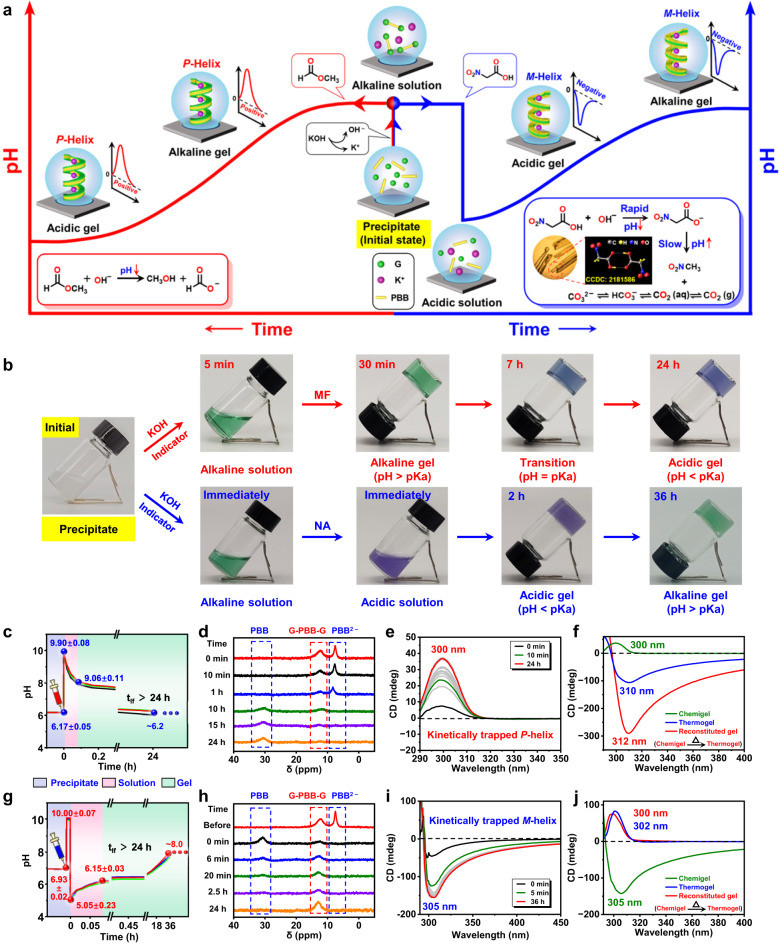
Kinetically trapped supramolecular chiral inversion. **a** Cartoon representing pH evolution profile for kinetically trapped programming of the self-assemblies. Left: KOH and MF system; Right: KOH and NA system. Inset: The POM image and single-crystal structure of NA. **b** Phase behaviors of G and PBB mixture after cycle initiation (Supplementary Movies 3 and 4). Methylene blue and neutral red (1:1) are used as a mixed indicator for monitoring pH changes (color change point: pH 7.0). **c–e** pH–time profiles (**c**), time-dependent ^11^B NMR spectra (**d**), and CD spectroscopy (**e**) after cycle initiation fueled with KOH and MF. **f** CD spectra of the as-prepared acidic chemigel, corresponding thermogel, and thermally annealed chemigel. **g–i** pH–time profiles (**g**), time-dependent ^11^B NMR spectra (**h**), and CD spectroscopy (**i**) after cycle initiation fueled with KOH and NA. **j** CD spectra of the as-prepared alkaline chemigel, corresponding thermogel, and thermally annealed chemigel.

More interestingly, when the chemical fuels (KOH/PrS) were replaced with KOH/methyl formate (MF) or KOH/nitroacetic acid (NA) system, kinetically trapped hydrogels with inverse helicities were achieved (Fig. 5a). These types of chemically fueled gels are referred to as “chemigels” as reported in the literature to distinct them from previously discussed thermogels prepared via heating–cooling cycles^16^. The spontaneous hydrolysis of MF in water over time leads to the decrease in the pH of the system (Fig. 5a)^66^. When mixing G and PBB (ratio: 2:1, 1.0% w/v in G, initial pH: 6.17 ± 0.05, the total volume was 5.0 mL) with aqueous KOH (350 μL, pH = 14) and MF (70 μL), the pH immediately jumped to 9.90 ± 0.08, which then gradually decreased and eventually got stabilized at *ca*. 6.2 after 24 h (Fig. 5c). Visual observation indicates that a hydrogel was formed across the pH range of *ca*. 9.0 to 6.2 and was maintained over time (Fig. 5b, Supplementary Movie 3). Rheological moduli of both G′ and G″ show an initial rapid increase, and then a rather slow decrease with time, which eventually stay relatively constant for several hours, thus validating stable gel state (Supplementary Fig. 27). Time-dependent ^11^B NMR spectra reveal that the chemical structure evolved from G-PBB-G diesters to G and PBB mixtures with the change in the pH from alkaline to acidic on addition of fuel (Fig. 5d). However, CD spectra show that the chirality of the hydrogel still maintained *P*-helix at the end of the reaction cycle (Fig. 5e). Further thermal annealing of this acidic chemigel (pH = *ca*. 6.2) from 80 to 25 °C with a cooling rate of 5 °C·min^−1^ reverted it to its thermodynamically favored state with *M*-helicity as observed in the thermogel under the same acidic conditions (Fig. 5f). Therefore, this acidic *P*-helical chemigel composed of G and PBB was a kinetically trapped structure, which could not autonomously transfer to the thermodynamic minimum state without heat absorption. The chemical reaction cycle, initiated by adding KOH/MF fuel, kinetically trapped the G and PBB into *P*-helical fibers by temporarily converting them into their corresponding G-PBB-G boronates. The existence of such a kinetically trapped state is likely related with the strong noncovalent interactions of the *P*-helical assemblies, producing high-energy barrier for conformation transformation^46,47^. It is worth noting that this “kinetically trapped” *P*-helix can only be obtained within a certain pH window since the gel disassemble and precipitate at very low pH levels, as discussed in the KOH/PrS system (Fig. 4). Additionally, cooling rate of the thermal annealing process did not influence the formation and chirality of gels, but did affect the intensity of the CD signals (Supplementary Fig. 28). An energy landscape for pathway dependent self-assembly of G and PBB driven by heat and chemical fuels is clearly demonstrated in Supplementary Fig. 29. Changes in MF concentration enable facile programing of the lifetime of the transient alkaline gels, the final pH of the acidic gels, as well as the intensity of the chiroptical signals (Supplementary Figs. 30 and 31 and Supplementary Table 7).

Next, when KOH and nitroacetic acid (NA) were used as chemical fuels and subsequently added to the system, subsequent changes in pH (pH_1_(high)–pH_2_(low)–pH_3_(high)) were achieved. NA is known to undergo alkaline-promoted decarboxylation in water, leading to the generation of nitroacetate that rapidly decreases the pH, which then further undergoes decarboxylation generating nitromethane and carbon dioxide that leads to the gradual increase in the pH (Fig. 5a, Supplementary Fig. 32)^67^. However, a similar compound, namely, trichloroacetic acid (TCA), although experiences similar alkaline-catalyzed decarboxylation^68^, does not exhibit this unusual pH-changing ability (Supplementary Fig. 32). NA was synthesized following the route described in Supplementary Fig 4. When G and PBB (ratio of 2:1, 0.5% w/v in G, initial pH of 6.93 ± 0.02, the total volume was 4.0 mL and the sample thickness was *ca*. 1 cm) were mixed in the presence of aqueous KOH (140 μL, pH = 14) and NA (18 mg), the pH of the system first increased to 10.00 ± 0.07, which then sharply dropped to 5.05 ± 0.23, and subsequently increased gradually and eventually got stabilized at *ca*. 8.0 after 36 h (Fig. 5g). Hydrogel was formed with the increase in the pH from 6.15 ± 0.03 to *ca*. 8.0 and then remained stable over time (Fig. 5b, g, Supplementary Movie 4). Compared with the solution system (Supplementary Fig. 32a), the rate of pH change of KOH and NA significantly decays in the presence of G and PBB due to gelation (Fig. 5g). Moreover, the thickness of the hydrogel also affects the rate of pH change, attributed to the different CO_2_ diffusion rates. During pH evolution, no apparent phase transition was noticed as evidenced by the rheological test (Supplementary Fig. 33, sample thickness 1.0 mm). In sharp contrast to the KOH and MF systems (Fig. 5e), CD spectra, in this case, showed an inverse negative chiroptical signal at ~305 nm (Fig. 5i), which also evolved with time and became saturated only after 30 min, indicating the successful realization of a stable and reverse *M*-helical supramolecular conformation. Despite the fact that the supramolecular chirality was retained during pH evolution, time-dependent ^11^B NMR spectra revealed the change of the molecular structure upon reversal of the pH. During continuous pH reversal, the G-PBB-G diesters dissociated rapidly at first and then gradually reformed (Fig. 5h). Also, a heating–cooling process was required to transform this kinetically trapped *M*-helical supramolecular nanostructure to its thermodynamically favored state with opposite *P*-helicity (Fig. 5j). These results demonstrate pathway dependent self-assembly of G and PBB driven by heat and chemical fuels (Supplementary Fig. 34). Change in the concentrations of NA led to a dramatic change in the minimum pH in this dynamic system, thereby changing the pH of the gelation point. This could eventually even reverse the chiroptical signals of the chemigels, demonstrating programmable supramolecular chirality (Supplementary Figs. 35, 36 and Supplementary Table 8). Notably, the CD spectra in Fig. 5i and 5e are not exact mirror images of each other, suggesting that the *M*-helicity observed in KOH/NA system is diastereomerically related to the *P*-helicity obtained in the KOH/MF system^8,69–71^. The mechanism of maintaining chirality through kinetic trapping driven by chemical fuels provides a general method for creating new chiral materials including liquid crystals and inorganic nanocrystals^72^. Recently, there have been reports on the preparation of transient silicon nanocrystals (SiNCs) and kinetically trapped gold nanoparticles using chemical fuels^47,73^. Unfortunately, these materials are not chiral. The development of chiral materials such as chiral crystals through the chemical fuel-driven kinetically trapped method could lead to distinct structures and properties resembling those found in living systems^74^.

### Chiroptical switching between the thermogels and the chemigels

**Fig. 6:**
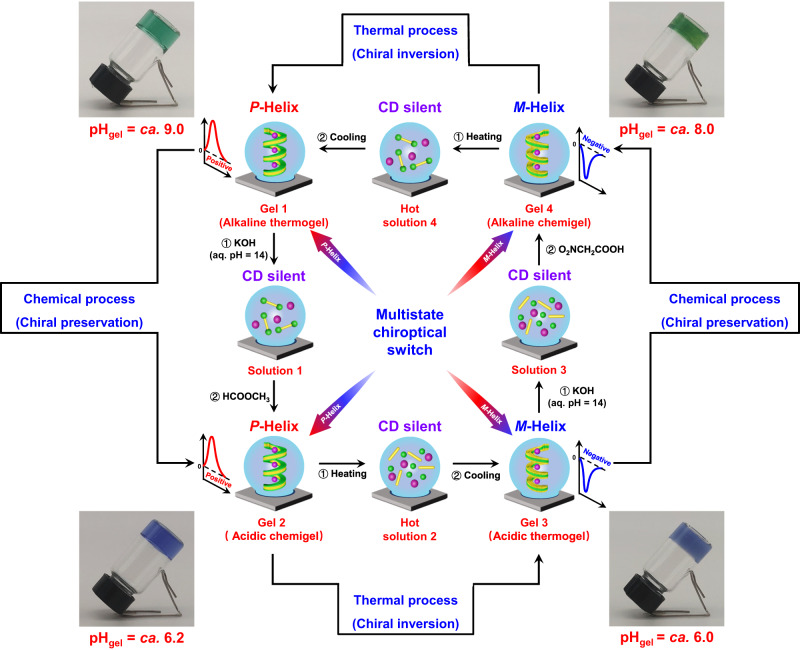
Chiroptical switching between the thermogels and the chemigels. Conducting successive chemical-fueled processes and thermal annealing process allowing sequential programmability of the supramolecular chirality, offering a three-state supramolecular chiroptical switch that can be recycled multiple times. Chemical processes retain the supramolecular chirality, whereas thermal processes reverse the supramolecular chirality. Methylene blue and neutral red (1:1, 5 mg·mL^−1^) are used as a mixed indicators for monitoring pH changes (color change point: pH 7.0).

The above-mentioned results clearly demonstrate the diverse chirality reversal of the G-PBB hydrogels in response to the pH and the preparative pathway (thermally or chemically). Surprisingly, successive conducting of chemical-fueled and thermal annealing processes allows for sequential programmability of the supramolecular chirality, offering an unusual example of a three-state supramolecular chiroptical switch that can be recycled multiple times (Fig. 6). Starting with an alkaline thermogel 1 (pH = *ca*. 9.0) with *P*-helicity made of G and PBB in aqueous KOH (pH = 12.5), addition of high concentration of aqueous KOH (pH = 14) results in an isotropic solution (Solution 1) with silent CD signal, which on subsequent addition of MF results in an acidic chemigel 2 (pH = *ca*. 6.2) with the same *P*-helicity. Thermal annealing of acidic chemigel 2 leads to an acidic thermogel 3 (pH = *ca*. 6.0) with inverse supramolecular chirality (*M*-helicity). Further addition of aqueous KOH with pH 14 to gel 3 produces an isotropic solution (Solution 3) with no observable CD signals. Subsequent addition of NA in solution 3 results in an alkaline chemigel 4 (pH = *ca*. 8.0) with the same *M*-helicity. Finally, a heating–cooling process reconverts the alkaline chemigel 4 back to the alkaline thermogel 1 (*P*-helicity), completing a full cycle of four accessible chiral aggregates. During the chemical processes, the supramolecular chirality was preserved; however, in the thermal steps, it was inversed. Refueling of gel 1 with KOH and MF restarted the chiroptical switch and the entire system could be recycled for at least three times. The detailed quantity of the added chemicals in each cycle and the experimental CD spectra during these processes are presented in Supplementary Table 9 and Supplementary Fig 37, respectively. Previously, most reported chiroptical switches were based on two types of switching phenomena driven by photo^7^, and seldom were based on three-state photoswitches^8,18^. In fact, similar to a photo stimulus, temperature is also a non-invasive stimulus that can be rapidly applied and removed. The heating and cooling rates are more readily adjustable, providing enhanced control over the chiroptical switchable systems^7,8,69,71,75^. The present multistate system is driven by heat and chemical fuels, thus opening new avenue for the development of smart chiroptical systems. It has great potential for creating chiral logic gates and for information storage and encryption, as well as for circularly polarized luminescence materials design and application^28,76–78^.

## Discussion

We report on a bio-inspired synthetic supramolecular system with programmable supramolecular chirality under non-equilibrium system. In equilibrium, G can interact with PBB to construct a wide range of pH assemblies with inverse supramolecular chirality. Underlying this behavior is an interchange of two G-quadruplex configurations presenting reverse chiroptical properties by acid/base-mediated dynamic boronic ester bonds formation and dissociation. Autonomous programming between these two pH-dependent supramolecular helical conformations was realized by generating a chemical reaction network driven by the internal pH reaction cycle. The fuel acids input demonstrates significant effects on the subsequent non-equilibrium self-assembly process, producing either transient or kinetically trapped chiral assembly with tunable right- or left-handed nanostructures. Further thermal annealing reverts the kinetically trapped hydrogels to their thermodynamically favored state with opposite helicity, thereby, affording a novel example of a three-state supramolecular chiroptical switch that can be recycled over multiple times. The present study paves a new way to gain precise control on chiral properties under non-equilibrium systems, which provides attractive new prospects for development of adaptable and controllable supramolecular chiral materials.

## Methods

### Materials

Guanosine (G, 98%) and Nitromethane (98%) were purchased from Alfa aesar. KOH (99.98%) was purchased from Acros Organics Co., Ltd. 1,3-Propanesulfonate (99%) and Trichloroacetic acid (Granulated) were purchased from Tokyo Chemical Industry. Piperazine anhydrous (99%), Formylphenylboronic acid (containing varying amounts of anhydride) (98%) and L-(+)-Tartaric acid (99%) were purchased from Shanghai Aladdin Biochemical Technology Co., Ltd. Methyl formate (MF, 99%) was purchased from Shanghai Macklin Biochemical Co., Ltd. Lithium hydroxide, anhydrous (99%) was purchased from Beijing InnoChem Science & Technology Co., Ltd. All other chemicals used in this study were analytical grade reagents.

### Characterization

Circular dichroism (CD) spectra were obtained on a Chirascan CD spectrometer where the path length was chosen appropriately. Corresponding temperature-dependent measurements were performed with a TC 125 temperature controller with adjustable heating/cooling rate. The FTIR spectra were collected with a Germany BRUKER Tensor 27 FTIR spectrometer with an ATR attachment. UV-visible spectroscopy measurements were performed on a HITACHI UH4150 spectrophotometry. Mass spectrometry experiments were performed on a Waters Xevo G2-XS QTof Spectrometer. Powder X-ray diffraction (PXRD) profiles were performed using a Bruker D8 Advance X-ray diffractometer at 25 °C. Atomic force microscopy (AFM) measurements were performed at ambient conditions with a Bruker Fastscan apparatus in tapping mode. NMR measurements were performed on a 600 MHz NMR spectrometer (JEOL ECZ600R/S3) equipped with a 14.09 T superconducting magnet and a 5.0 mm 600 MHz broadband Z-gradient high-resolution ROYAL probe (JEOL RESONANCE Inc., Japan). All ^11^B NMR spectra were obtained at 192.43 MHzC. The ^11^B background signal from the NMR probe and the NMR tube was removed by performing backward linear FID prediction. The chemical shift references of ^11^B NMR and ^1^H NMR were BF_3_OEt_2_ and TMS (or D_2_O), respectively. Rheological tests were performed by using a Hake Mars rheometer (Thermo Scientific) with a parallel plate geometry (20 mm diameter, 1.0 mm gap). Crystal images were obtained by 59XC polarizing microscope. Crystal data was obtained using a Rigaku oxford diffraction SuperNova single crystal diffractometer with graphite-monochromated using Cu Kα X-ray source (λ = 1.5418 Å) with ω-scan technique at room temperature. The structure was solved using SHELXS (direct methods) and refined by SHELXL (full-matrix least-squares techniques) in the Olex2 package. All non-hydrogen atoms were refined using anisotropic displacement parameters. Hydrogen atoms attached to carbon were placed in geometrically idealized positions and refined using a riding model.

Cryogenic SEM (Cryo-SEM). Cryogenic scanning electron microscopy (cryo-SEM) measurements were conducted using a Regulus 8100 field emission scanning electron microscope. The sample, applied to a copper holder, was mounted onto a stub shuttle before being rapidly frozen by plunging into slush (a mixture of solid and liquid nitrogen). The frozen sample was then transferred using a sealed vacuum transfer device to the precooled stage of the preparation chamber, which was attached to the SEM. After slicing, the sample was sublimated at −85 °C for 10 min. It was then sputter-coated with platinum at 10 mA for 30 s. Images were taken at −140 °C under an accelerated voltage of 5 keV.

Cryogenic TEM (Cryo-TEM). The lacey carbon support film copper grids were glow discharged for 45 s before placing a few microliters of each sample on them (TED PELLA, INC). The sample-containing grids were then vitrified in liquid ethane using the Virtobot (Thermofisher, Shanghai Nanoport, China) and cryo-transferred via the Elsa™ holder (Gatan, model 698, ultra-low profile) to the Thermofisher Talos F200X transmission electron microscopy with Ceta camera in bright field mode at an acceleration voltage of 200 kV. During image acquisition by EPU software, the samples were kept close to liquid nitrogen temperature. To reduce beam damage and increase contrast, a 30 µm objective aperture and a total dose of 20 e^−^/Å^2^, along with a defocus of −3 μm, were used.

Small-angle X-ray scattering (SAXS) experiments were performed on a Nano-inXider (λ = 0.154 nm, Xenocs, France). The scattering amplitude for a cylindrical aggregate is given by:

1 $${{\emptyset }}\left({{{{{\rm{q}}}}}},{{{{{\rm{R}}}}}}\right)={\left(2v\frac{{{{{{\rm{sin }}}}}}\left(\frac{{qL}{{{{{\rm{cos }}}}}}\theta }{2}\right)}{\frac{{qL}{{{{{\rm{cos }}}}}}\theta }{2}}\frac{{J}_{1}\left({qR}\,{{{{{\rm{sin }}}}}}\theta \right)}{{qR}{{{{{\rm{sin}}}}}} \theta }\right)}^{2}$$

Here *R* is the average radius of the cylinder; *L* and ν denote the height and volume of the cylinder, respectively. *J*_1_ is the first-order Bessel function. *θ* denotes the angle between the scattering vector *q* (*q* = 4πsin *θ*/λ, λ is the wavelength of X-ray) and the cylinder axis.

### Preparation of thermally-triggered hydrogels (thermogels)

Considering G-PBB-pH_aq.7_ hydrogel (0.5% w/v) as an example, thermogels were prepared as follows. Briefly, G (25.0 mg, 0.0883 mmol), PBB (15.4 mg, 0.0441 mmol), and KCl (3.3 mg, 0.0441 mmol) were added into a clean vial (G:PBB:K^+^ = 2:1:1), which was followed by the addition of distilled water (5.0 mL). The vial was heated to completely dissolve the mixture, and then, cooled down to room temperature, yielding a translucent hydrogel, which was able to support its weight upon vial inversion. For G-PBB-pH_aq.1–6_ hydrogels, the distilled water was replaced with corresponding concentrations of HCl solution. For G-PBB-pH_aq.8–14_ hydrogels, the distilled water was replaced with corresponding concentrations of KOH solution. Additional KCl was added if necessary to ensure gelation (keeping the ratio of G:PBB:K^+^ as 2:1:1).

### Preparation of chemical fuels-triggered hydrogels (chemigels)

For preparing chemigels, KOH and 1,3-propanesulfonate (PrS) system was considered as a representative example. To a precipitate of G and PBB in water (50.0 mg G and 30.9 mg PBB in 5.0 mL water), the KOH fuel (15 mg, unless mentioned otherwise) was added. The precipitates were dissolved by stirring for only 1–2 min. Subsequent addition of PrS (100 μL, unless mentioned otherwise) led to a hydrogel formation after a certain time, which turned into precipitates with time. The full cycle was monitored using a pH meter (FiveEasy Plus FE28, Mettler Toledo). The preparation of other chemigels fueled with KOH/MF and KOH/NA followed a similar procedure. For refueling experiments, samples were prepared following a similar procedure as described above. The detailed amounts of the chemicals for each cycle are presented in Supplementary Tables 5–9. The lifetime of the transient hydrogels is defined by the time they are no longer self-supporting by the vial inversion test.

### Supplementary information

Supplementary Information

Peer Review File

Description of Additional Supplementary Files

Supplementary Movie 1

Supplementary Movie 2

Supplementary Movie 3

Supplementary Movie 4

### Source data

Source Data

## Data Availability

The data that support the findings of this study are available within this Article and its Supplementary Information. Source data are provided with this paper and available in figshare database under 10.6084/m9.figshare.23576619. The X-ray crystallographic coordinates for nitroacetic acid reported in this study have been deposited at the Cambridge Crystallographic Data Centre (CCDC), under deposition numbers 2181586. These data can be obtained free of charge from The Cambridge Crystallographic Data Centre via www.ccdc.cam.ac.uk/data_request/cif. Source data are provided with this paper.
