# Supplementary material for: Programmable supramolecular chirality in non-equilibrium systems affording a multistate chiroptical switch
Source: Nat Commun. 2023 Aug 18;14:5030. doi: 10.1038/s41467-023-40698-9 (PMC10439165; doi:10.1038/s41467-023-40698-9)
Supplement: Supplementary file 1 — Supplementary Information [file 41467_2023_40698_MOESM1_ESM.pdf]

Supplementary Information

Programmable supramolecular chirality in non-equilibrium systems affording a multistate chiroptical switch

Jingjing Li,[1] Yihan Cui,[2] Yi-Lin Lu,[3] Yunfei Zhang,[2] Kaihuang Zhang,[2] Chaonan Gu,[2] Kaifang Wang,[1,2] Yujia Liang,[1,2] and Chun-Sen Liu*,[2]

[1] School of Chemistry and Chemical Engineering, Henan University of Technology, Zhengzhou, 450001, China.

[2] College of New Energy, Zhengzhou University of Light Industry, Zhengzhou, 450002, China.

[3] Department of Chemistry, Key Laboratory of Advanced Energy Materials Chemistry (Ministry of Education), Nankai University, Tianjin, 300071, China.

E-mail: nkchunsenliu@163.com (C.-S. Liu).

Supplementary Methods

1. Synthesis of 3,3'-piperazine-bis(benzoxaborol)

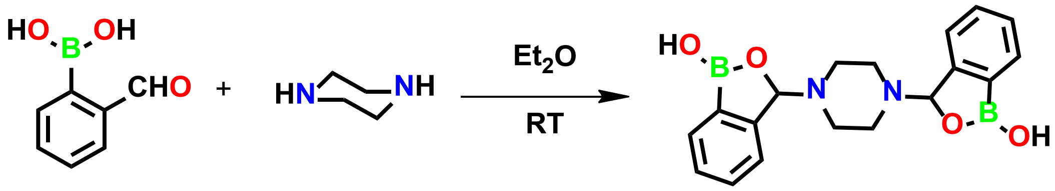

Supplementary Figure 1. Synthesis route of 3,3'-piperazine-bis(benzoxaborol) (PBB).

PBB was synthesized by a previously reported method with slight modification.[1] A solution of 2-formylphenylboronic acid (9.60 g, 64 mmol) in diethyl ether (500 mL) was prepared in a 1000-mL three-necked round bottomed flask equipped with a mechanical stirrer and a dropping funnel. Solution of piperazine (2.70 g, 32.2 mmol) in diethyl ether (360 mL) was slowly added dropwise (about 1 drop per second) into the 2-formylphenylboronic acid solution with stirring. After addition of about 15% of the piperazine solution, a fine white solid precipitated. The entire piperazine solution was then poured into the suspension and the reaction mixture was stirred for another 1 h and then left for 24 h at room temperature. The solid was filtered off, then washed twice with diethyl ether, and finally dried in vacuum, which afforded 10.20 g PBB (92% yield) as white solid, m.p. 219.5–220.8 °C (lit.[1]: 220 °C). ^{1}H-NMR (600 MHz, DMSO-d_6): δ 9.17 (1H, s, BOH), 9.15 (1H, s, BOH), 7.72-7.31 (8H, m, Ph), 5.85 (1H, s, CH), 5.80 (1H, s, CH), 2.59 (4H, br, m, CH_2CH_2), 2.42 (4H, br, m, CH_2CH_2); ^{13}C-NMR (600 MHz, DMSO-d_6): δ 153.05, 132.70, 131.42, 131.37, 130.66, 128.59, 122.95, 96.11, 46.88.

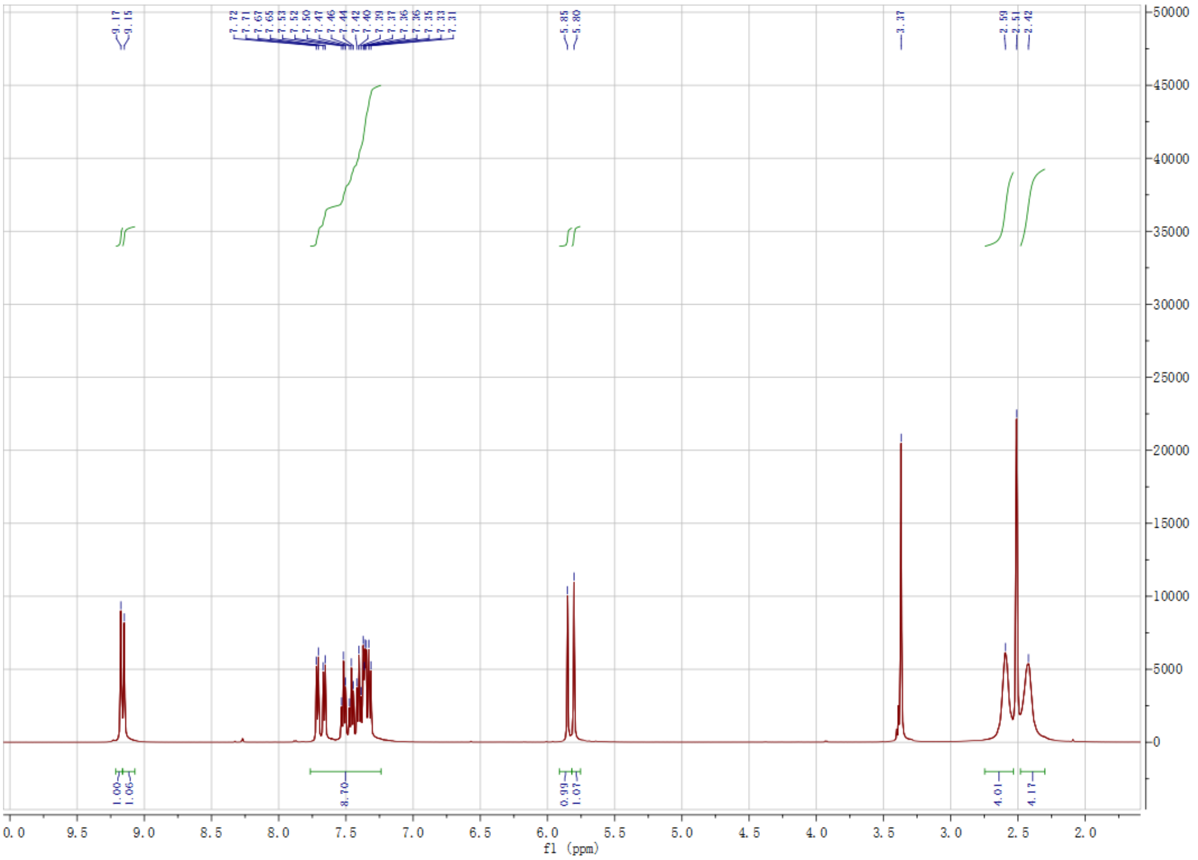

Supplementary Figure 2. ^{1}H NMR spectrum of 3,3'-piperazine-bis(benzoxaborol) in DMSO-d_6.

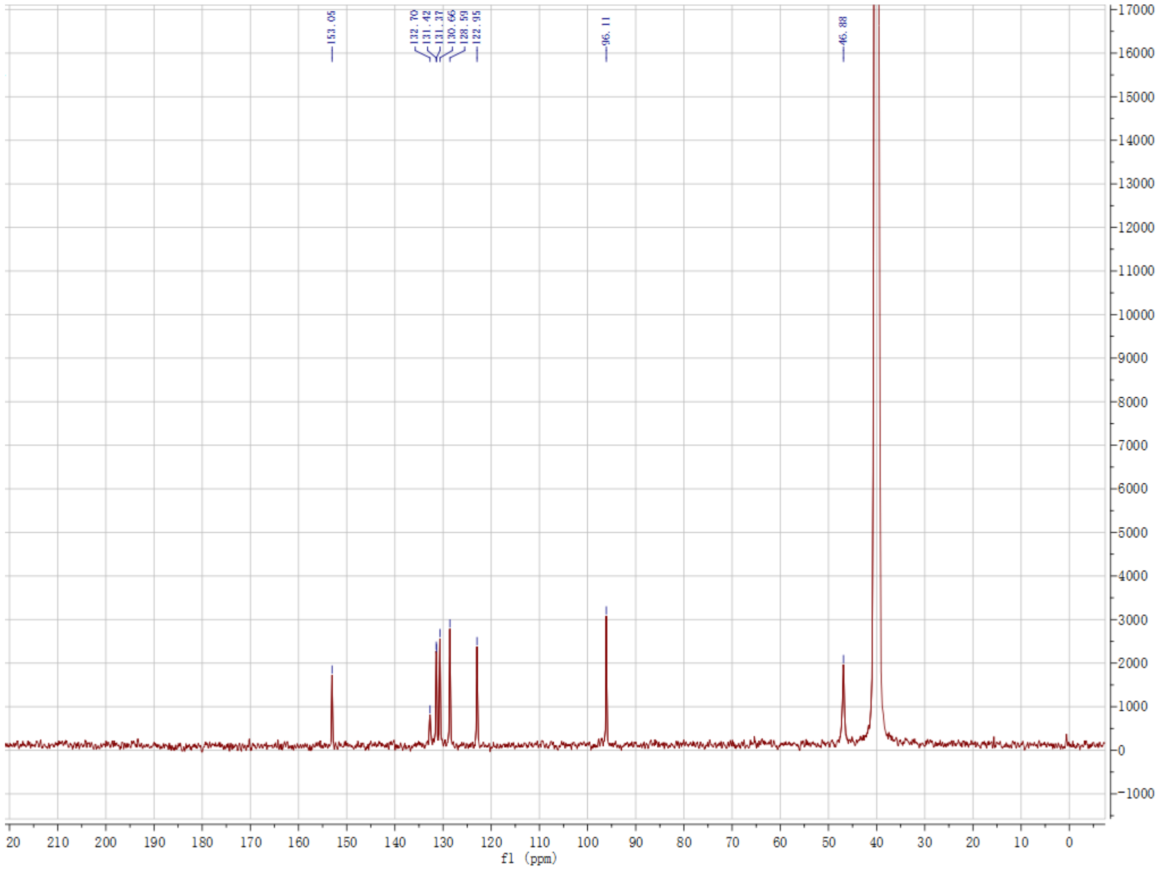

Supplementary Figure 3. ^{13}C NMR spectrum of 3,3'-piperazine-bis(benzoxaborol) in DMSO-d_6.

2. Synthesis of nitroacetic acid

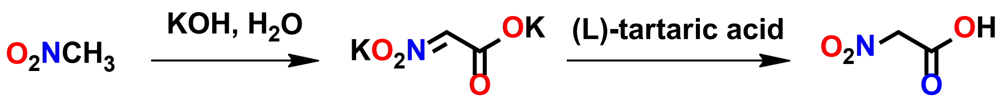

Supplementary Figure 4. Synthesis route of nitroacetic acid (NA).

Nitroacetic acid (NA) was synthesized by following a previously reported method with slight modification.[2,3] In a 1000-mL three-necked round bottomed flask equipped with a thermometer and a condenser, KOH (241.94 g, 4.312 mol) was dissolved in water (242 mL) with stirring and the temperature was gradually increased during addition. When the temperature reached 67 °C, nitromethane (56.4 mL, 1.048 mol) was slowly added dropwise. The temperature was further increased to 95 °C during the addition. Furthermore, the temperature was maintained between 95 and 100 °C for 7 h. Cooling the flask to room temperature afforded dipotassium nitroacetate as yellow crystals. The crystals were filtered and washed with cold methanol. The product (27.24 g, 28.7%) was then dried overnight under vacuum and used without any further purification.

In a 250-mL round bottomed flask, dipotassium nitroacetate (9.0 g, 49.8 mmol) was first dissolved in cold water (30.0 mL) with stirring. The resulting orange solution was then cooled down to −15 °C in an ice–brine bath, and a cold solution (–10 °C) of L-tartaric acid (64.2 g, 428.4 mmol) in water (120 mL) was added dropwise while maintaining the temperature between −5 to −15 °C. After addition, the mixture was stirred at −5 to −15 °C for 4 h. After the completion of the reaction, the solid was filtered and the filtrate was then extracted with cold anhydrous diethyl ether (3 × 70 mL). The organic layer was combined and dried over anhydrous magnesium sulfate and concentrated under reduced pressure to afford yellow oil. The as-obtained oil was then dissolved in cold chloroform and concentrated under reduced pressure. The dissolution and concentration process was repeated three times (3 × 30 mL), which afforded NA (2.27 g, 25.4%) as yellowish crystalline solid. Recrystallization of NA from chloroform led to the formation of high-quality colorless rod-shaped crystals, whose structure was confirmed by single-crystal XRD (CCDC:2181586, Supplementary Table 1). For more details about the crystal data of nitroacetic acid, see the Crystallographic Information File CCDC-2181586. This data can be obtained free of charge from The

Cambridge Crystallographic Data Center *via* www.ccdc.cam.ac.uk/data_request/cif. Notably, the compound was stable as a solid, but it started decomposing in solution. Precisely, NA used in this study was always freshly prepared and stored under −30 °C dry conditions, m.p. 81.7–83.4 °C (single crystals), lit.[3]: 84–85 °C (powders). ^{1}H NMR (600 MHz, CD_3CN) δ: 5.30 (s, 2H); ^{13}C-NMR (600 MHz, CD_3CN): δ 163.90, 77.03.

3. Procedure for pKa determination

Potentiometric titrations: Potentiometric titrations were performed using a Mettler Toledo pH meter (FiveEasy Plus FE28). The pH meter was first calibrated with five buffer solutions (pH = 1.68, 4.00, 6.86, 9.18, and 12.46). PBB with a concentration of 0.001 M was firstly dissolved in 0.01 M KOH solution (100 mL), and then titrated with 0.01 M HCl solution at 25 °C.

Spectrophotometric Titrations: Spectrophotometric titrations were performed under conditions analogous to those used for the potentiometric measurements. PBB with a concentration of 0.001 M was prepared in solutions with different pH. The UV–vis spectroscopy measurements were performed using HITACHI UH4150 spectrophotometer at 25 °C. The optical path length was 1.0 cm. The pKa was calculated by using the Henderson–Hasselbach equation.[4]

4. Computer simulations

Conformation searches were performed for the building blocks of the assemblies by molecular dynamics (MD) simulations by using xTB software.[5] The conformations for each building block, which were obtained by dynamic quenching by using Grimme's extended tight-binding model (GFN0-XTB),[6] were further optimized by using Grimme's extended tight-binding model (GFN2-xTB)[7] to screen out the most stable conformation for each building block. Then, the most stable conformation was used to construct assemblies. Geometry optimizations of assemblies were also performed by using Grimme's extended tight-binding model (GFN2-xTB).[7] The theoretical ECD spectra of the simulated self-assembled structure were calculated using time-dependent density functional theory (TD-DFT) in combination with the B3LYP [8-11] method and def2svp[12-13] basis set. As there were a large number of atoms in the self-assembled system, we calculated the ECD spectra of the helically arranged motifs derived from the self-assembled G-quadruplex structures. Molecular graphs were visualized by using CYLView.[14]

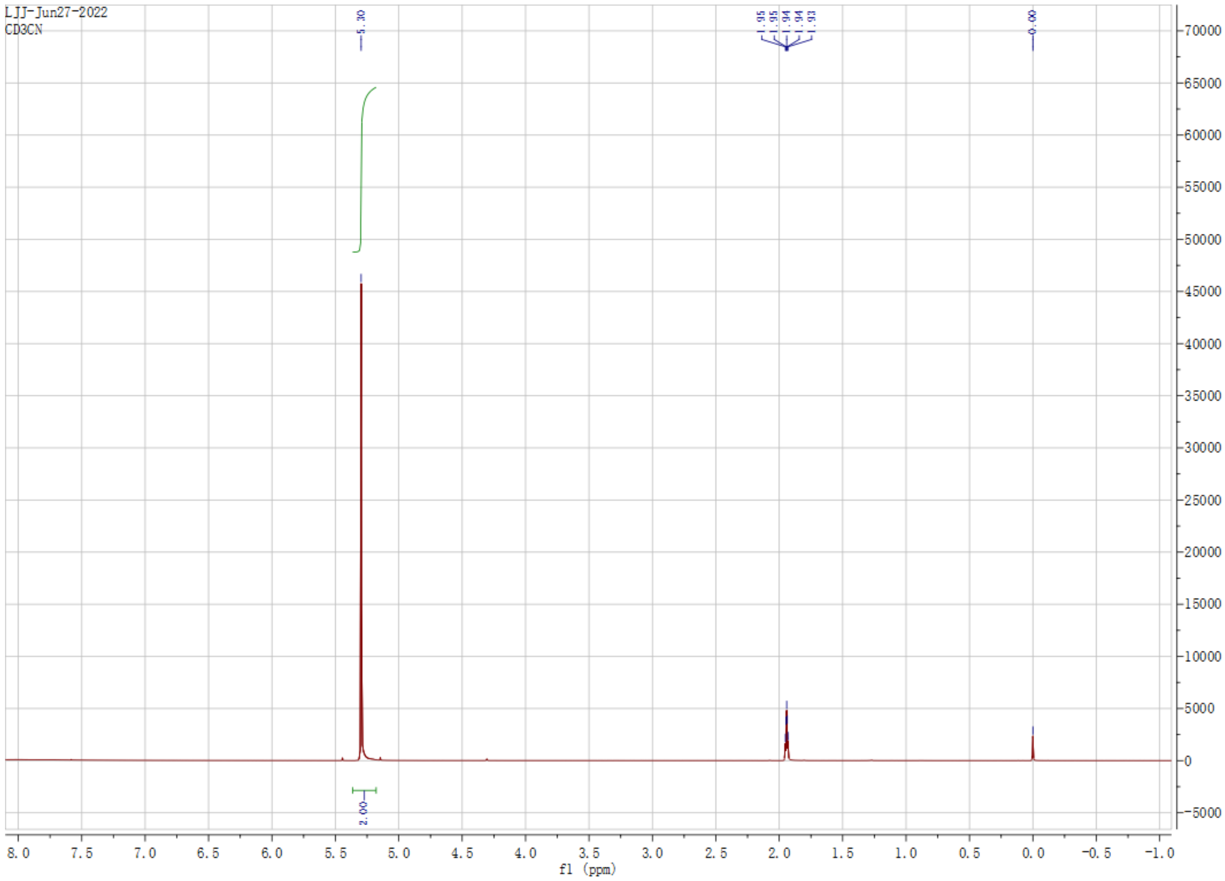

Supplementary Figure 5. ^{1}H NMR spectrum of nitroacetic acid in CD_3CN.

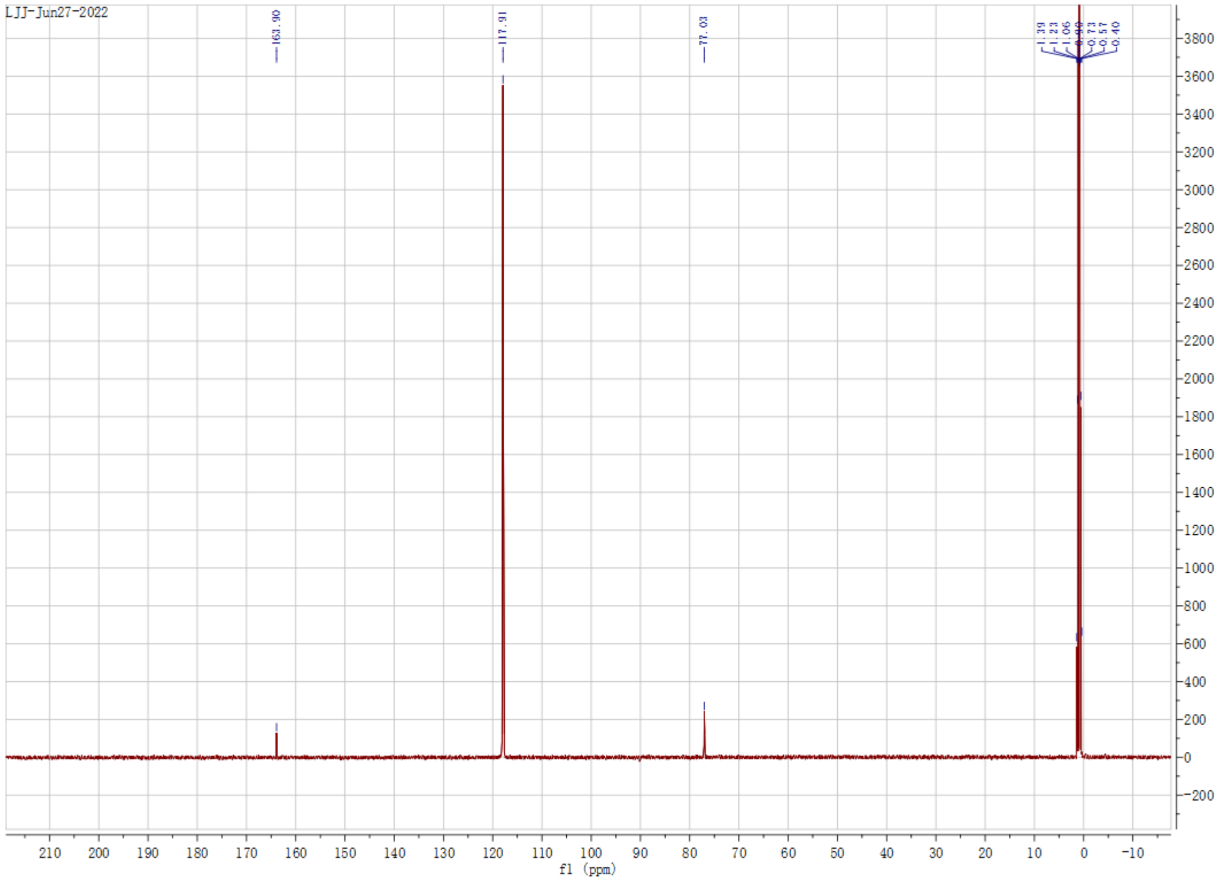

Supplementary Figure 6. ^{13}C NMR spectrum of nitroacetic acid in CD_3CN.

Supplementary Table 1. Crystal data and structure refinement for nitroacetic acid.

Empirical formula	$C_2H_3NO_4$
Formula weight	105.05
Temperature/K	293.78(11)
Crystal system	monoclinic
Space group	$P2_1/n$
a/Å	5.2178(5)
b/Å	11.0568(10)
c/Å	7.5408(8)
α/°	90
β/°	107.716(11)
γ/°	90
Volume/Å^3	414.41(7)
Z	4
ρ_{calc}g/cm^3	1.684
μ/mm^{-1}	1.516
F(000)	216.0
Crystal size/mm^3	0.12 × 0.08 × 0.04
Radiation	CuKα (λ = 1.54184)
Index ranges	$-5 \leq h \leq 4$, $-12 \leq k \leq 12$, $-8 \leq l \leq 8$
Reflections collected	1059
Independent reflections	632 [R_{int} = 0.0167, R_{sigma} = 0.0277]
Data/restraints/parameters	632/0/65
Goodness-of-fit on F^2	1.161
Final R indexes [I>=2σ (I)]	R_1 = 0.0910, wR_2 = 0.1864
Final R indexes [all data]	R_1 = 0.0939, wR_2 = 0.1934
Largest diff. peak/hole / e Å^{-3}	0.56/-0.78

Supplementary Table 2. Gelation properties of G-PBB-pH$_{aq.n}$ hydrogels.

Solvents[a]	pH = 1	pH = 2	pH = 3	pH = 4	pH = 5	pH = 6	pH = 7	pH = 8	pH = 9	pH = 10	pH = 11	pH = 12	pH = 13	pH = 14
Appearance[b]	OG	OG	TG	TG	TG	TG	TG	TG	TG	TG	TG	CG	CG	CG
CGCs (w/v)[c]	4.0%	2.0%	0.5%	0.5%	0.5%	0.5%	0.5%	0.5%	0.5%	0.5%	0.5%	0.5%	1.5%	17.0%
T_{gel} (°C)[d]	88.1–89.2	79.9–81.6	63.6–64.4	65.0–66.0	63.4–66.2	65.6–66.8	66.9–67.6	64.3–66.2	64.8–65.3	65.9–66.1	65.9–66.3	66.9–67.1	68.7–69.6	65.7–67.1
pH_{gel}[e]	3.51 ± 0.12	5.33 ± 0.13	6.00 ± 0.05	6.06 ± 0.06	6.08 ± 0.06	6.06 ± 0.08	6.07 ± 0.09	6.13 ± 0.08	6.11 ± 0.12	6.15 ± 0.08	6.17 ± 0.07	7.55 ± 0.05	10.40 ± 0.05	11.19 ± 0.06

a: pH values were adjusted by using different concentrations of KOH or HCl solution, for example, the solution with pH 1 was a 0.1 M solution of HCl, the solution with pH 13 was a 0.1 M KOH solution. b: OG: opaque gel; TG : translucent gel; CG: clear gel. c: Molar ratio of G and PBB was fixed to 2:1 in all pH range and the concentration referred to the starting concentration of G. Additional KCl was added to ensure gelation with a ratio of 2:1:1 for G, PBB and K^+ if necessary. d: T_{gel} was determined by falling ball method at their CGCs. e: the actual pH of the hydrogels at their CGCs.

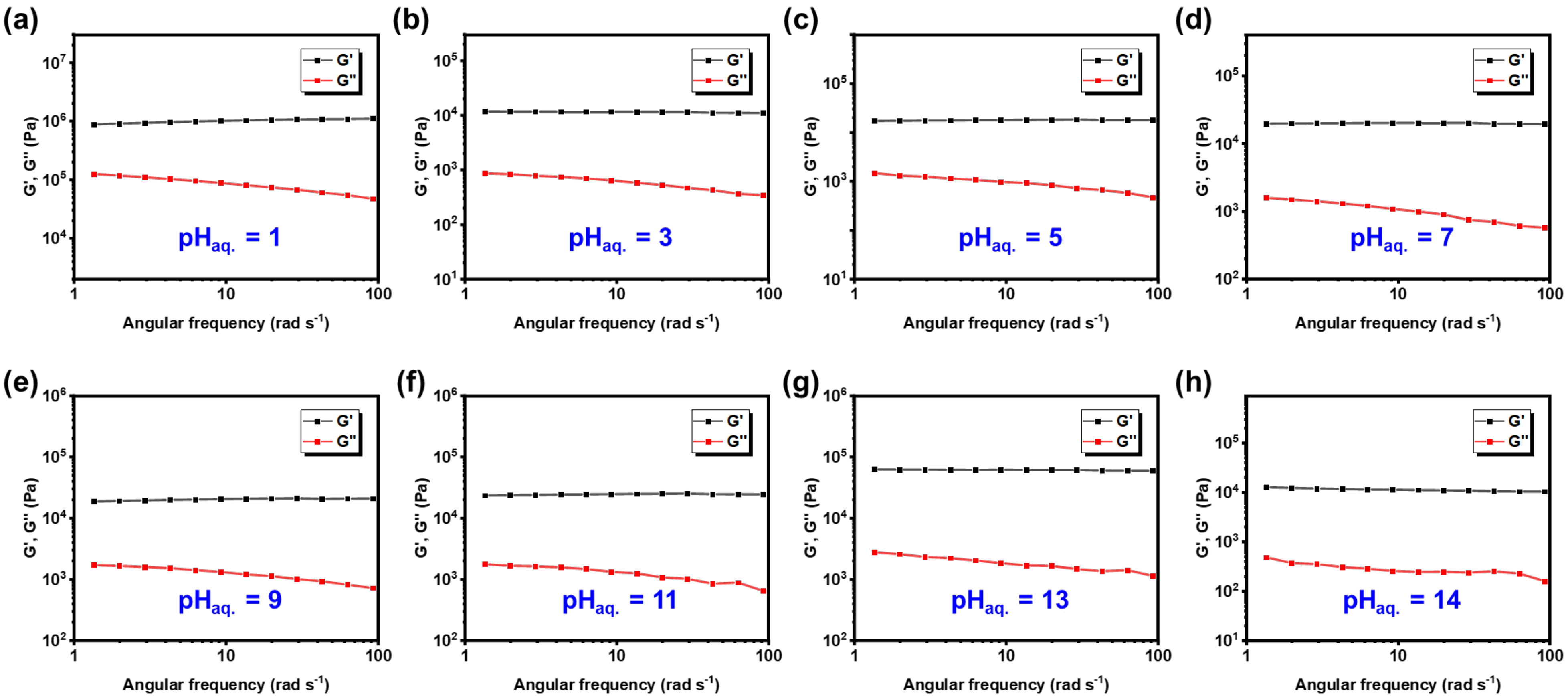

Supplementary Figure 7. Dynamic frequency sweep of G-PBB-pH$_{aq.n}$ hydrogels. Pannels (a-h) are for G-PBB-pH$_{aq.1}$, G-PBB-pH$_{aq.3}$, G-PBB-pH$_{aq.5}$, G-PBB-pH$_{aq.7}$, G-PBB-pH$_{aq.9}$, G-PBB-pH$_{aq.11}$, G-PBB-pH$_{aq.13}$ and G-PBB-pH$_{aq.14}$ hydrogels, respectively. All samples were measured at their CGCs.

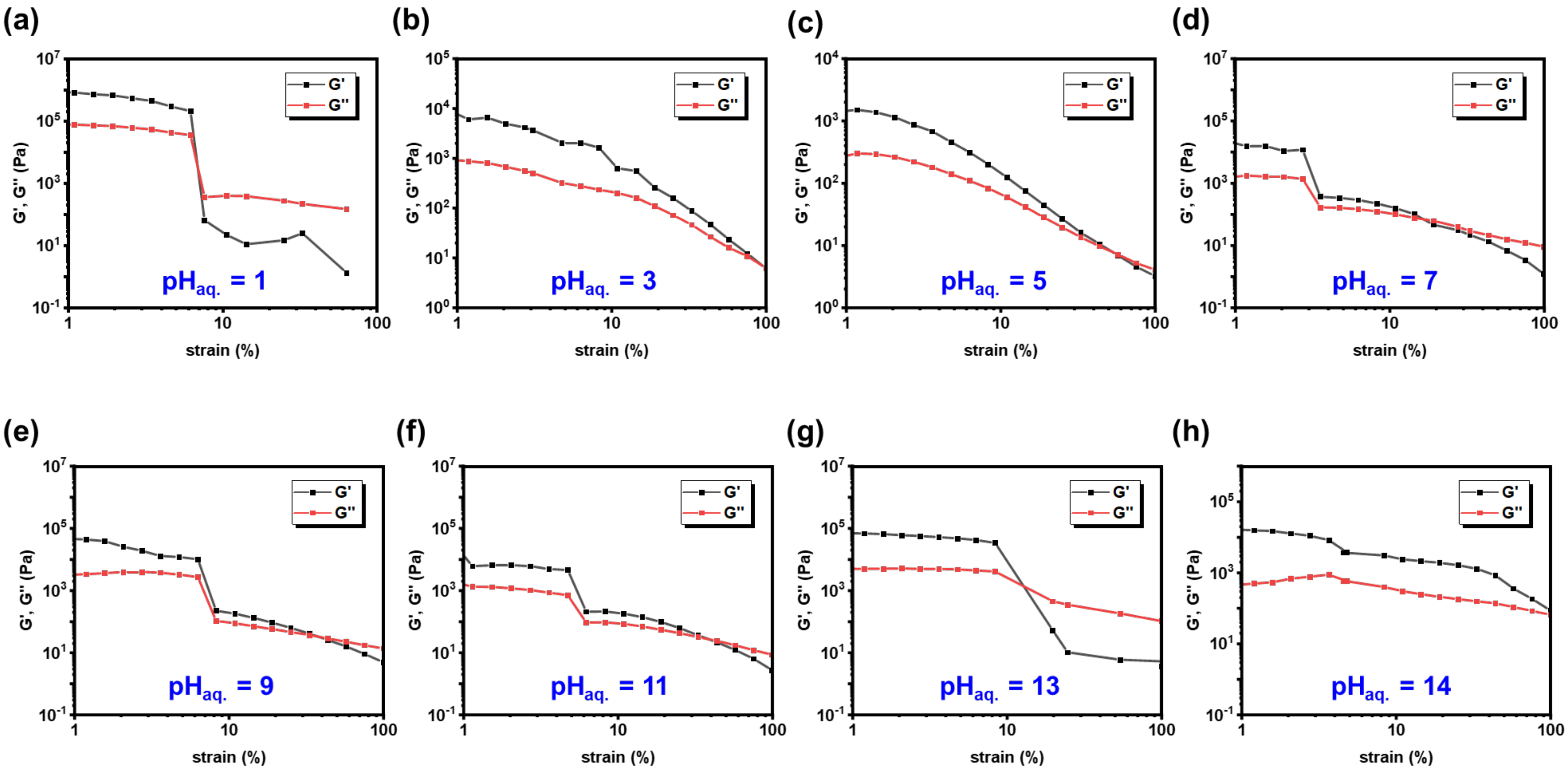

Supplementary Figure 8. Dynamic strain sweep of G-PBB-pH$_{aq.n}$ hydrogels. Pannels (a-h) are for G-PBB-pH$_{aq.1}$, G-PBB-pH$_{aq.3}$, G-PBB-pH$_{aq.5}$, G-PBB-pH$_{aq.7}$, G-PBB-pH$_{aq.9}$, G-PBB-pH$_{aq.11}$, G-PBB-pH$_{aq.13}$ and G-PBB-pH$_{aq.14}$ hydrogels, respectively. All samples were measured at their CGCs.

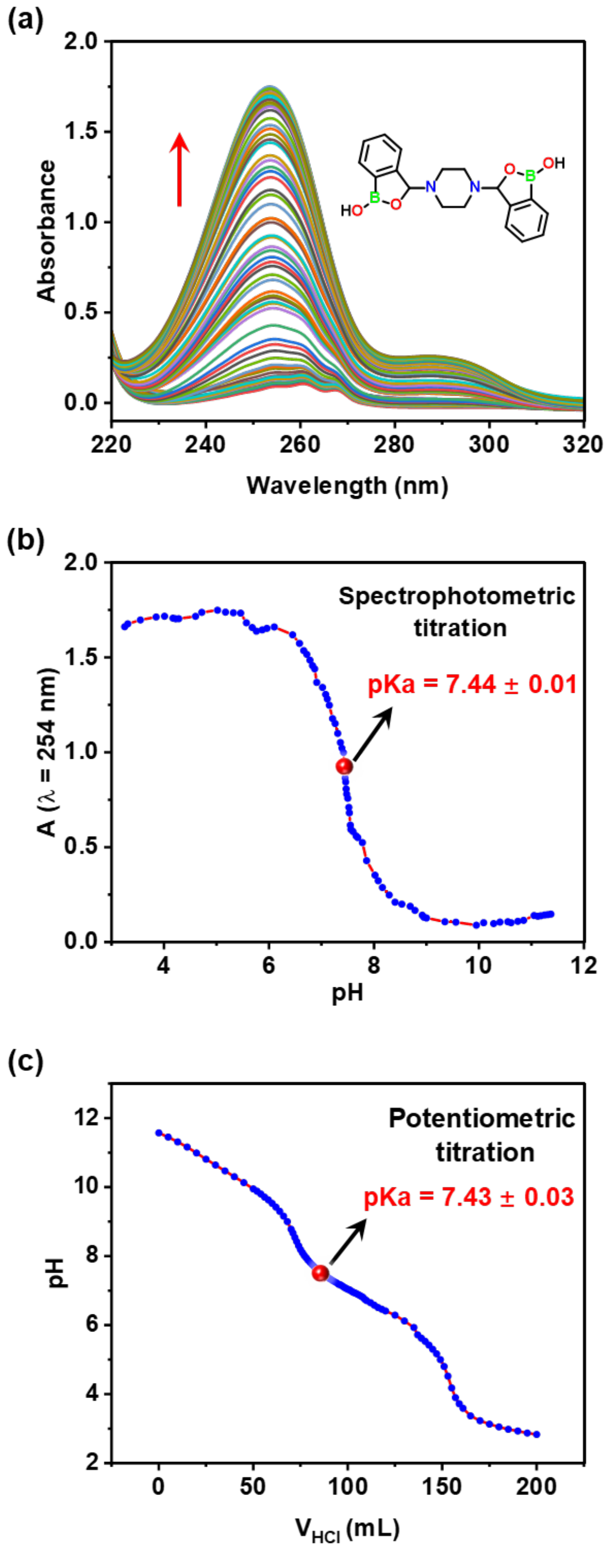

Supplementary Figure 9. (a) Spectrophotometric titration of PBB in water with varying pH (optical pathlength = 1.0 cm). Arrow indicates changes in the absorbance with respect to the decrease of pH. (b) Absorbance change at 254 nm vs. pH. (c) Potentiometric titration of PBB in water. PBB with a concentration of 0.001 M was firstly dissolved in 0.01 M KOH solution (100 mL), and then titrated with 0.01 M HCl solution at 25 °C.

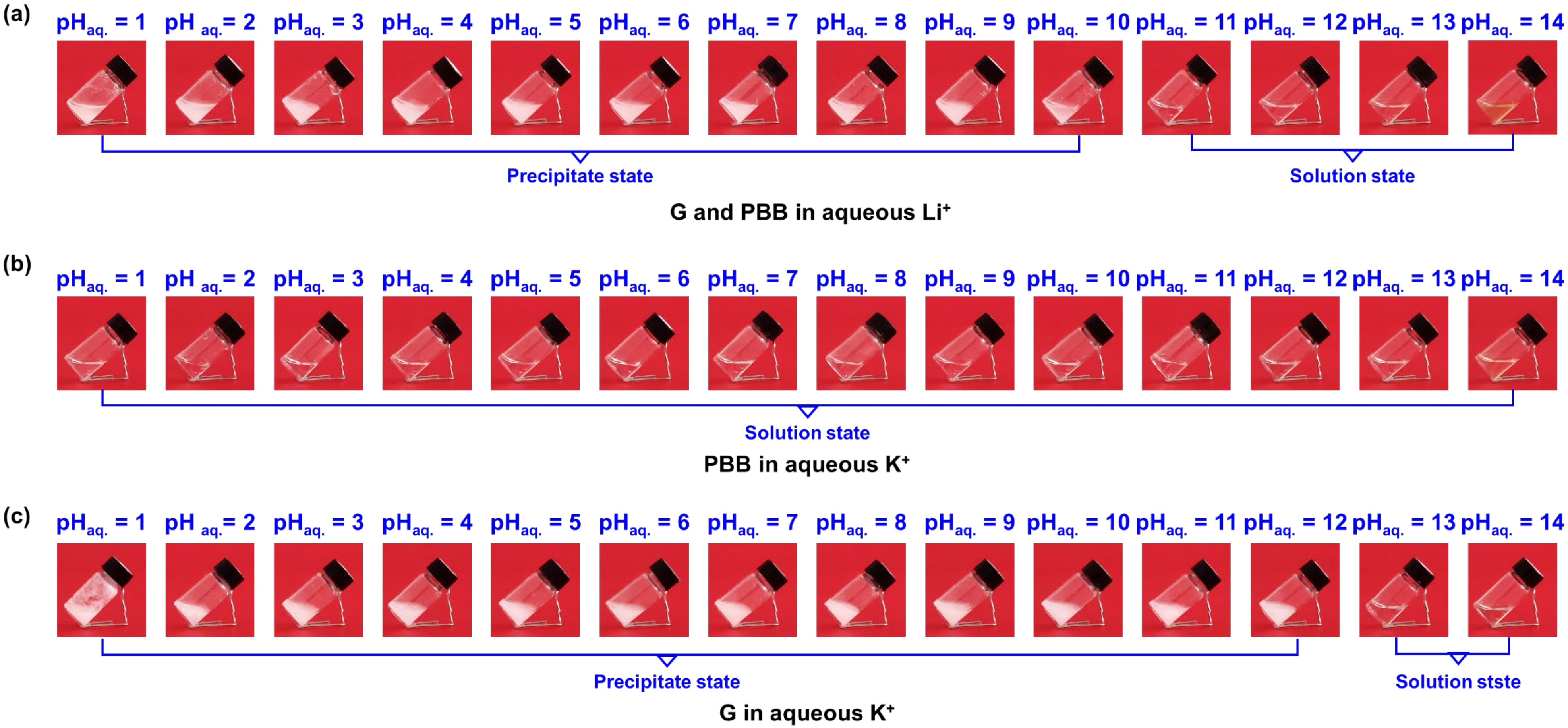

Supplementary Figure 10. Gelation behaviors of (a) G and PBB mixture in aqueous Li^+, (b) PBB in aqueous K^+ and (c) G in aqueous K^+. The samples were prepared in aqueous solutions with different pH.

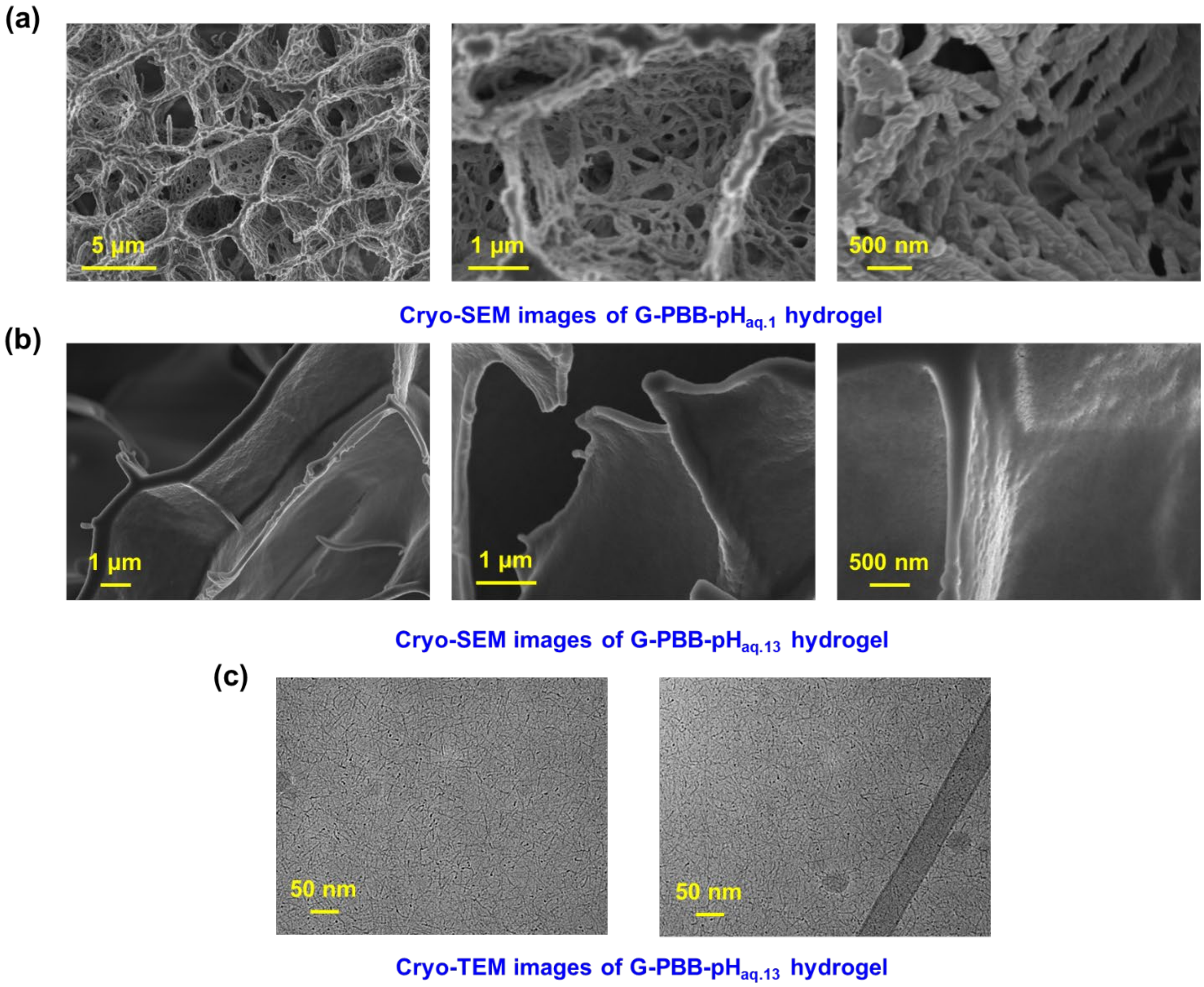

Supplementary Figure 11. Cryo-SEM images (a) G-PBB-$pH_{aq.1}$ and (b) G-PBB-$pH_{aq.13}$ hydrogels at different magnifications. (c) Two large cryo-TEM images G-PBB-$pH_{aq.13}$ hydrogels, which clearly shows the ultrathin fibers.

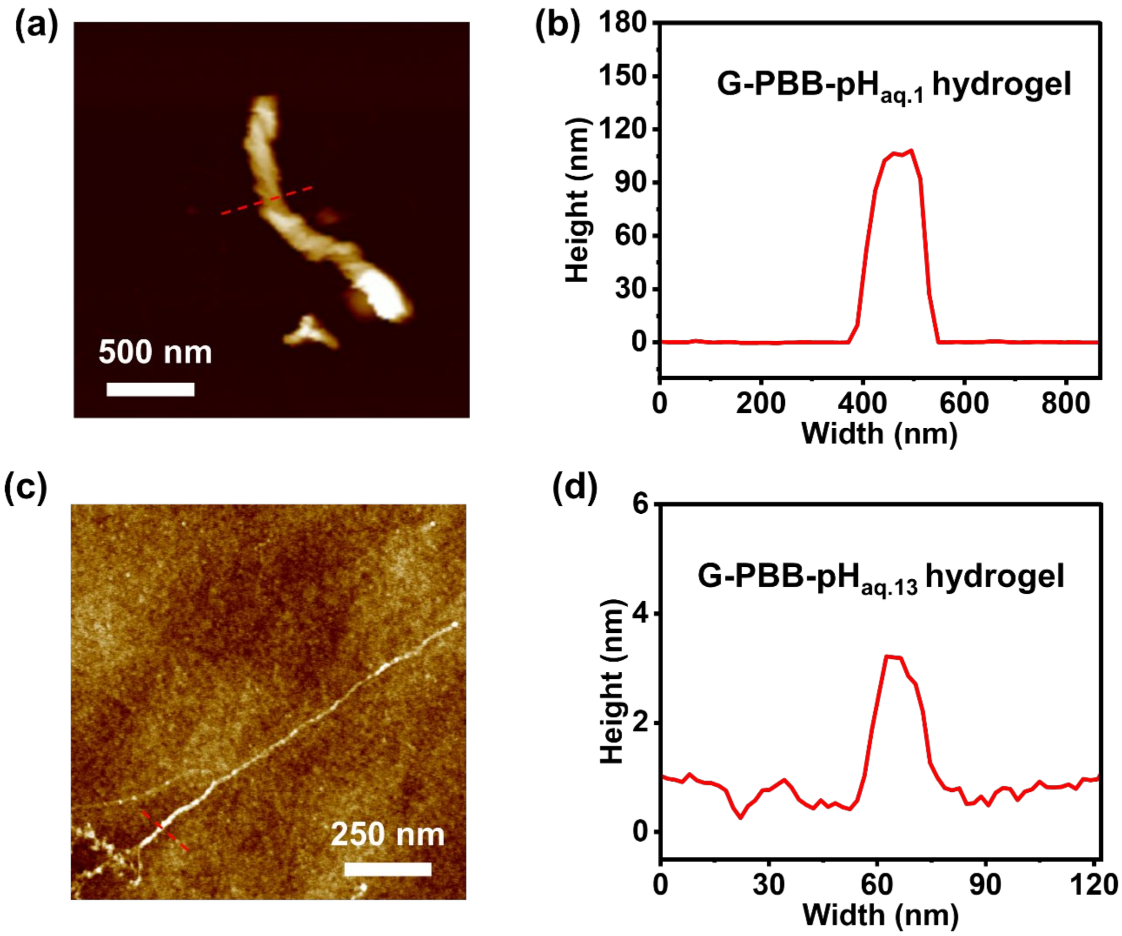

Supplementary Figure 12. AFM images of (a) G-PBB-pH$_{aq.1}$ and (c) G-PBB-pH$_{aq.13}$ hydrogels on silicon substrates and their height profiles (b and d) along the red lines.

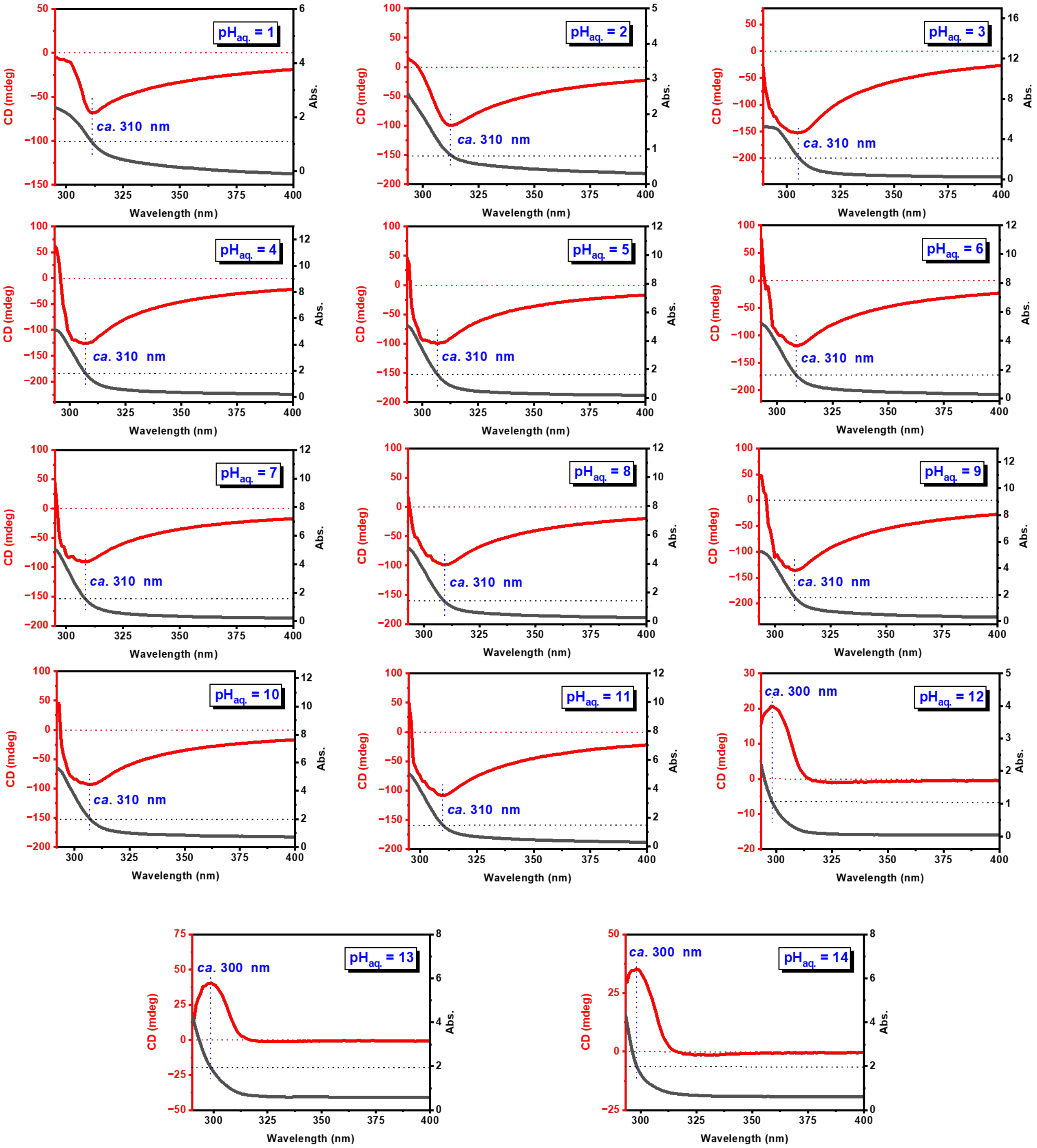

Supplementary Figure 13. The CD and UV-vis spectra of G-PBB-pH$_{aq.n}$ hydrogels (n = 1−14) at their CGCs (for data optimization, optical paths of opaque G-PBB-pH$_{aq.1\text{-}2}$ hydrogels were 0.1 mm, others were 0.5 mm).

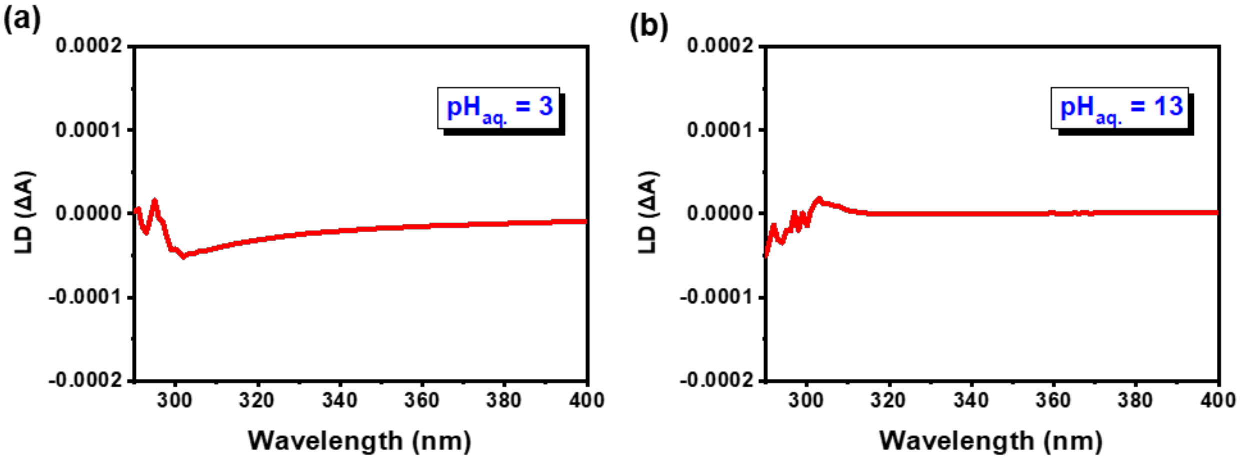

Supplementary Figure 14. LD spectra of two representative hydrogels with inverse CD response: (a) G-PBB-$pH_{aq.3}$ hydrogel and (b) G-PBB-$pH_{aq.13}$ hydrogel.

Supplementary Table 3. Thermodynamic parameters describing the self-assembly of two representative G-PBB-$pH_{aq.n}$ hydrogels (n = 3 or 12).

Sample	$C^{(a)}$ (mM)	T_m (K)	$\Delta H^{(b)}$ (kJ mol^{-1})	$\Delta H^{(c)}$ (kJ mol^{-1})	$\Delta S^{(c)}$ (J mol^{-1} K^{-1})	$K_e^{(d)}$ (M^{-1})
G-PBB-$pH_{aq.12}$	17.6	314.5	−242.3	−229.8	−695.8	5.3×10^3
G-PBB-$pH_{aq.3}$	17.6	314.1	−350.8	−351.0	−1087.8	5.2×10^4

(a) Corresponding to 0.5% w/v of G (G: PBB = 2:1).

(b) Values come from model calculations.

(c) Values come from Van't Hoff plot.

(d) Values determined at 300.15 K.

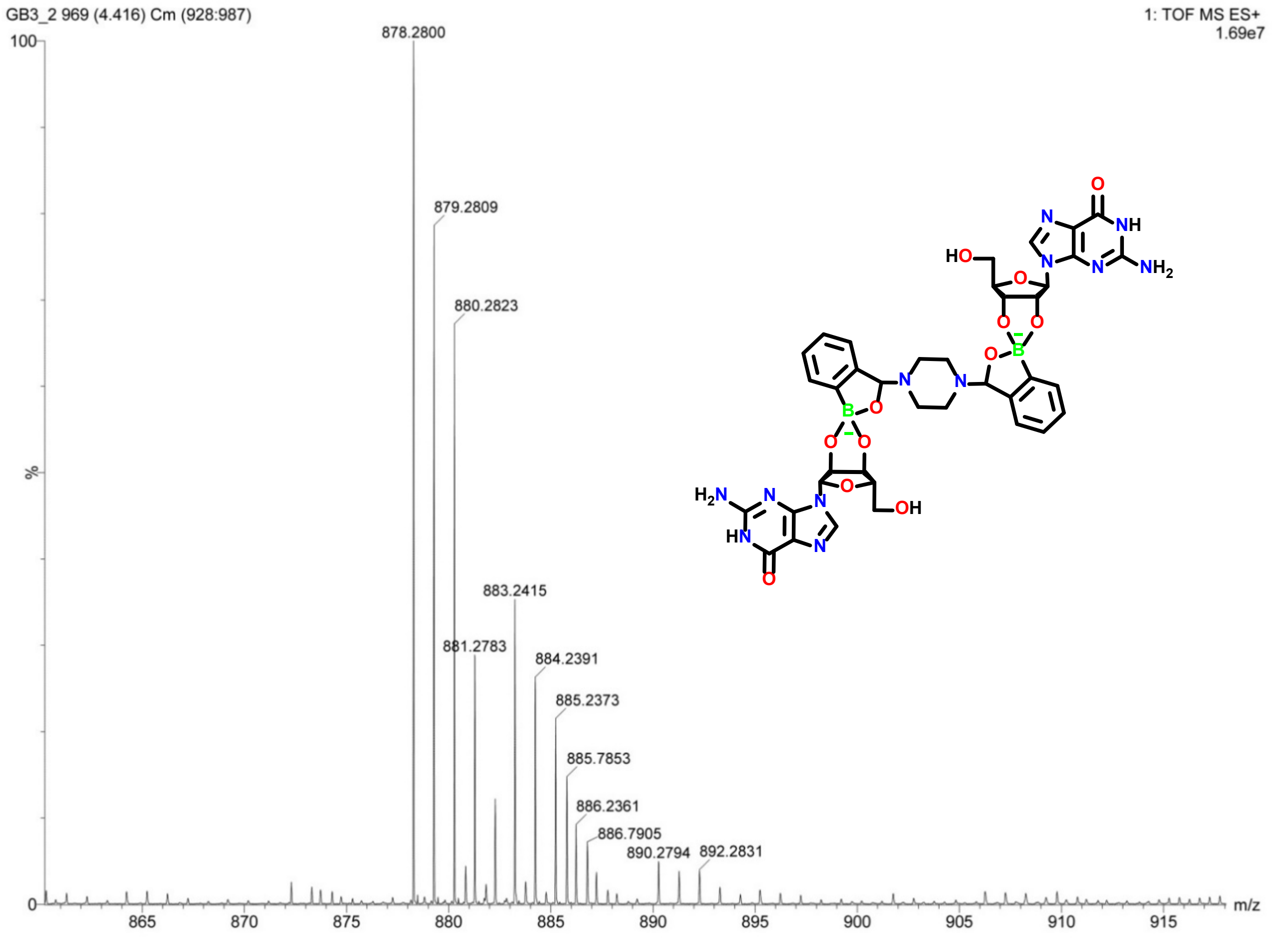

Supplementary Figure 15. The mass spectra of G-PBB-pH$_{aq.13}$ xerogel in methanol, showing the existence of G-PBB-G diester (the peak at 878.2800).

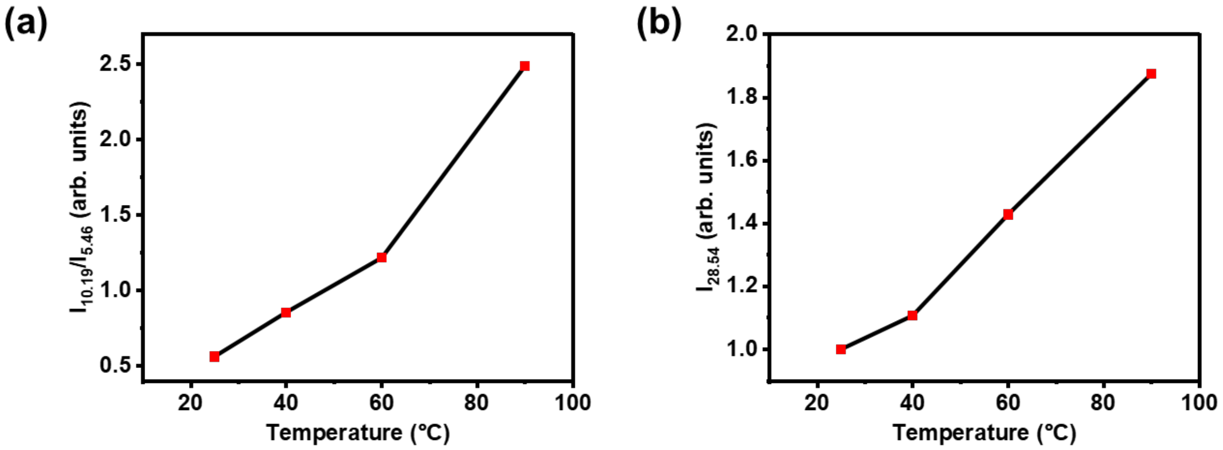

Supplementary Figure 16. (a) The relative intensity of the two peaks ($I_{10.19}/I_{5.46}$) in Figure 3b in the main text as a function of temperature. (b) The normalized intensity of the single peak ($I_{28.54}$) in Figure 3e in the main text as a function of temperature.

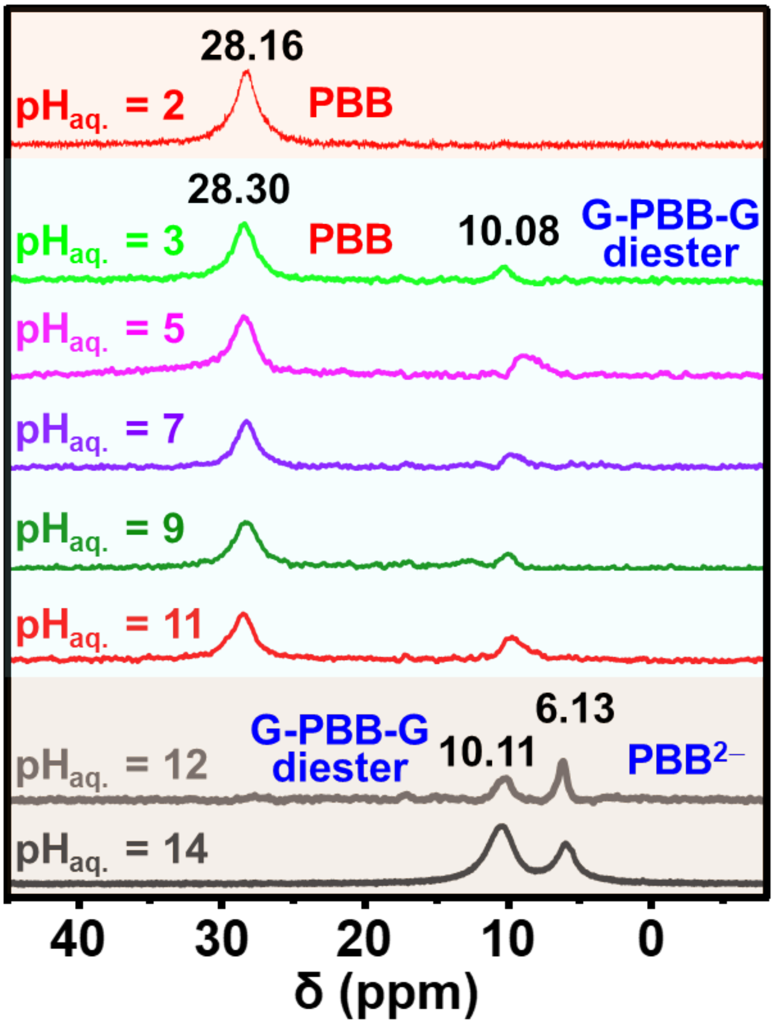

Supplementary Figure 17. ^{11}B NMR spectra of G-PBB-pH$_{aq.n}$ hydrogels.

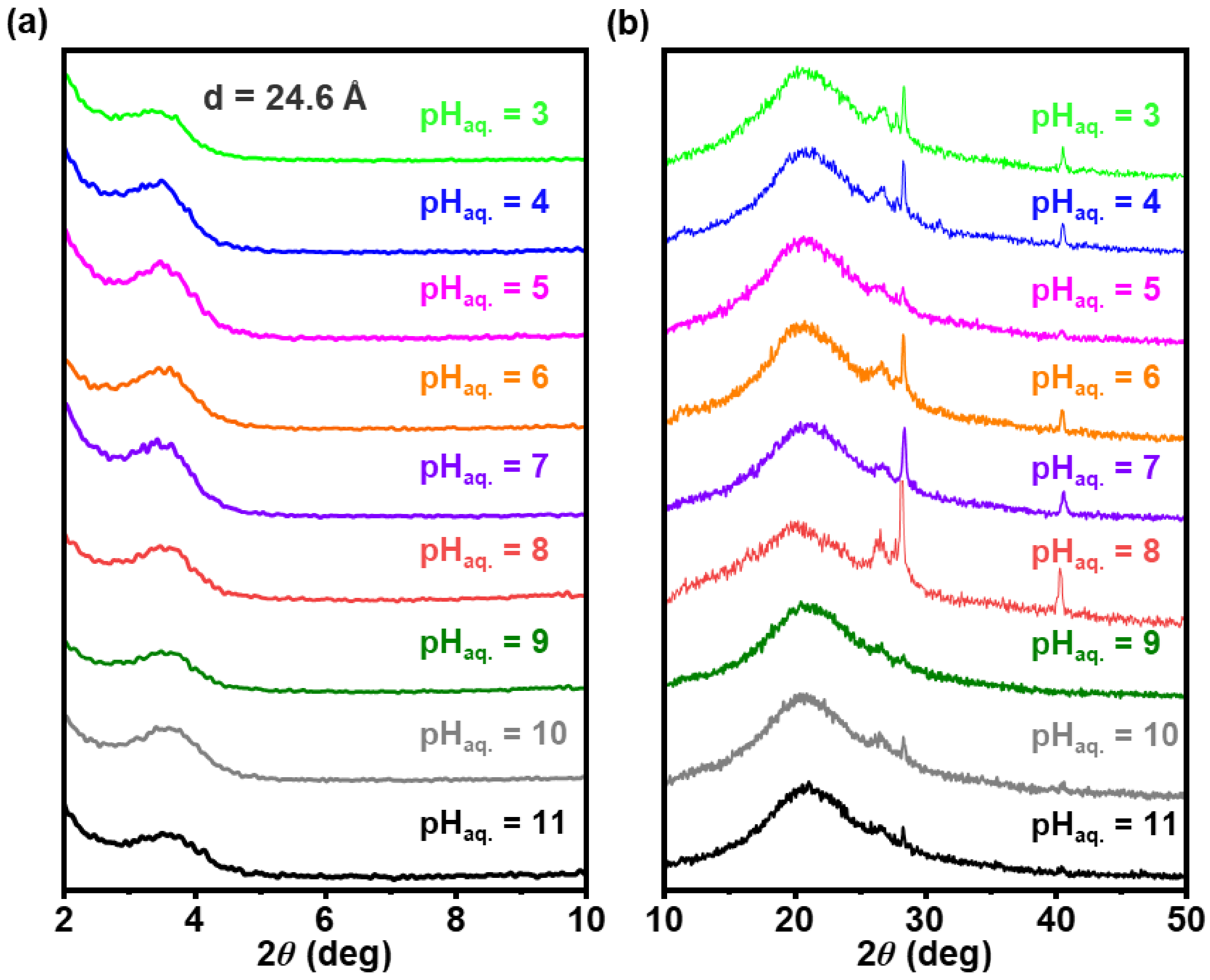

Supplementary Figure 18. (a) Small-angle XRD and (b) wide-angle XRD patterns of G-PBB-$pH_{aq.n}$ hydrogels.

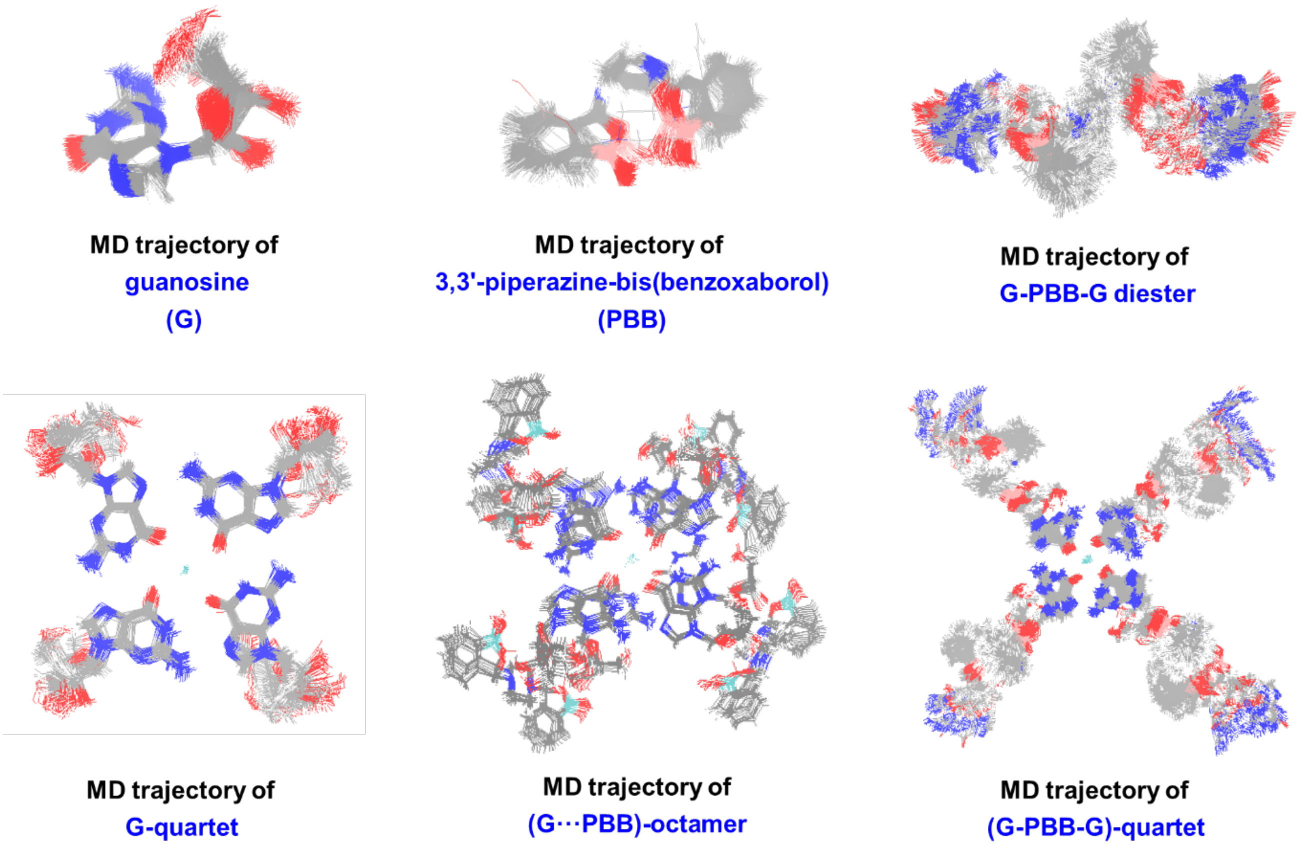

Supplementary Figure 19. The track diagrams of MD simulations for G, PBB, G-PBB-G diester, G-quartet, (G···PBB)-octamer and (G-PBB-G)-quartet.

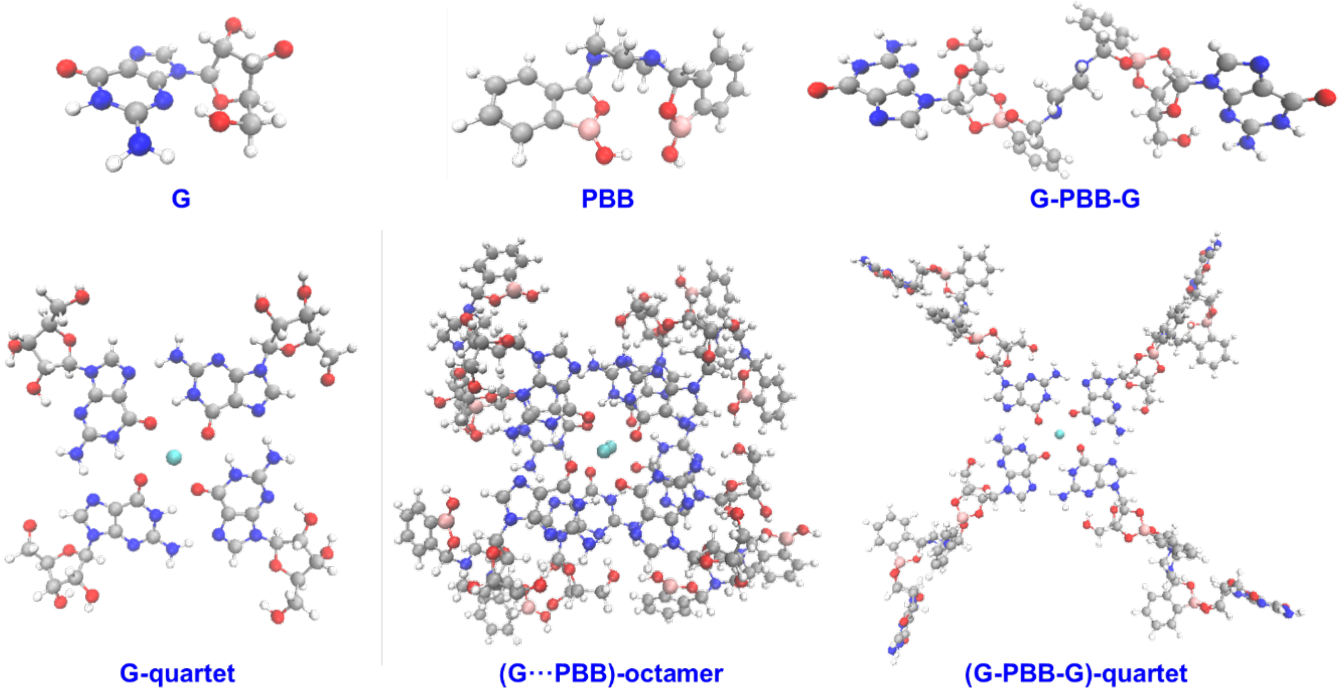

Supplementary Figure 20. The optimized geometries of the most stable conformations for building blocks.

Supplementary Table 4. GFN2-xTB calculated electronic energies of the structures discussed in the text.

Species	E (Hartree)	Species	E (Hartree)
**G**	–63.0626	**(G-PBB-G)-quadruplex**	–3018.3999
**PBB**	–73.5160	**G-quartet**	–252.3195
**G-PBB-G**	–188.9611	**(G···PBB)-octamer**	–798.9261
**(G-PBB-G)-quartet**	–755.6772	**(G···PBB)-hexadecamer**	–1450.9429
**(G-PBB-G)-octamer**	–1510.2908	**(G···PBB)-quadruplex**	–3048.8067

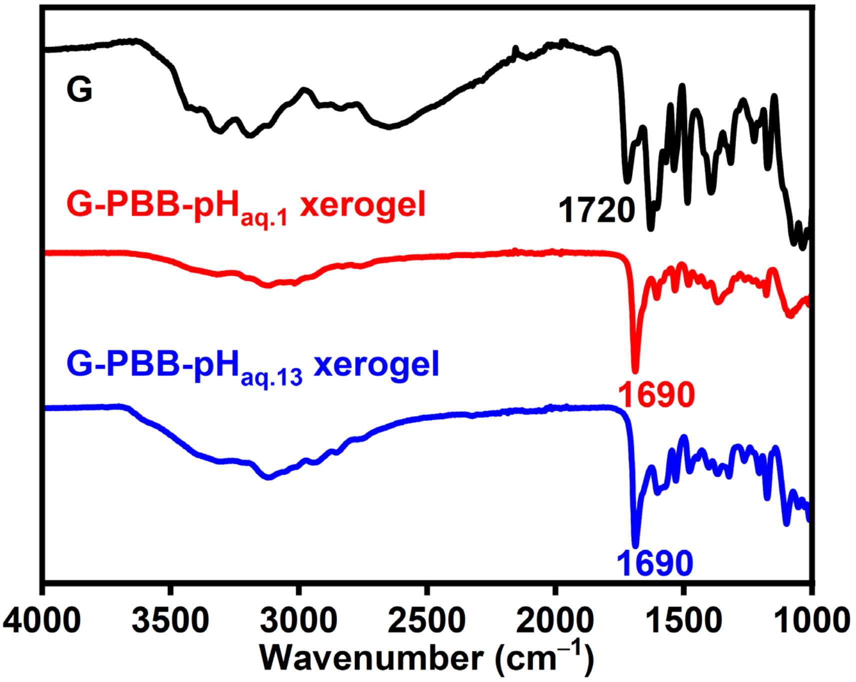

Supplementary Figure 21. FTIR spectra of free G, G-PBB-$pH_{aq.1}$ and G-PBB-$pH_{aq.13}$ xerogels.

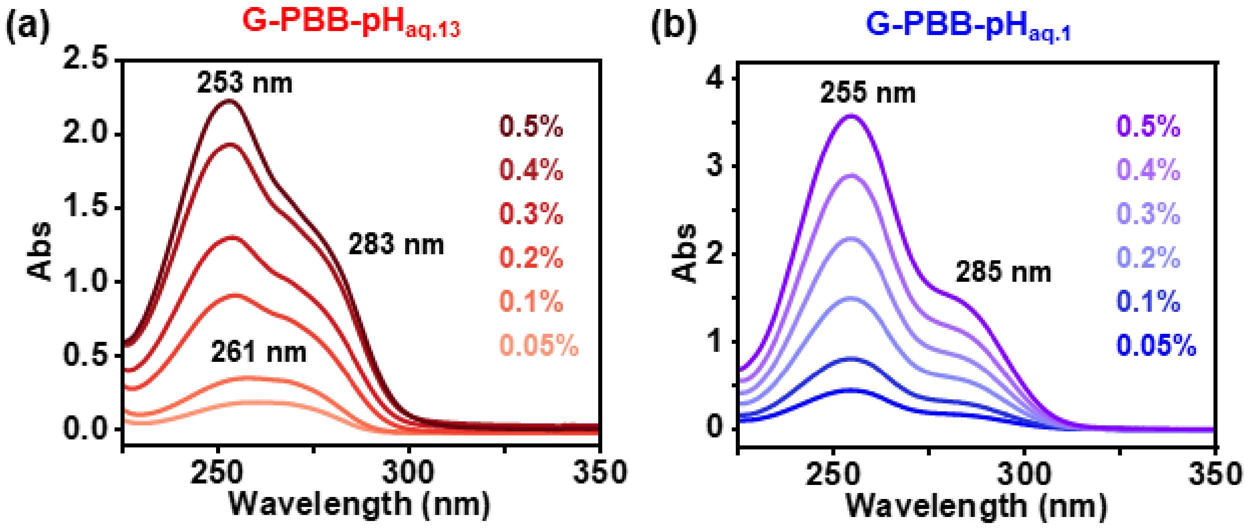

Supplementary Figure 22. Concentration-dependent UV-vis spectra of (a) G-PBB-pH$_{aq.13}$ hydrogel and (b) G-PBB-pH$_{aq.1}$ hydrogel.

Under alkaline condition, considering G-PBB-pH$_{aq.13}$ hydrogel as a representative example, the low wavenumber shift of C6=O vibration band in free G from 1720 to 1690 cm^{-1} on gelation indicates the formation of (G-PBB-G)-quartet, with C6=O oxygens being involved in H-bonding (Supplementary Figure 21).[15,16] concentration-dependent UV–vis spectra showed a typical blue shift of the absorption maximum upon self-assembly (from 261 to 253 nm) (Supplementary Figure 22), indicating H-type π–π stacking of (G-PBB-G)-quartets,[17] forming higher-ordered (G-PBB-G)-quadruplex structure.[18] For acidic G-PBB-pH$_{aq.1}$ hydrogels, concentration-dependent UV–vis spectra showed only aggregated structures at 255 nm and no visible shift occurred at all concentrations (Supplementary Figure 22). This result indicates that aggregation occurred even at sub-micromolar concentrations. However, for FTIR spectra, again, a low wavenumber shift of the C6=O vibration band of G was observed upon self-assembly from molecularly dissolved state to gel state (Supplementary Figure 21). These results provide strong evidence for involvement of intermolecular hydrogen bonding and π–π stacking interactions for formation of the G-PBB hydrogels.

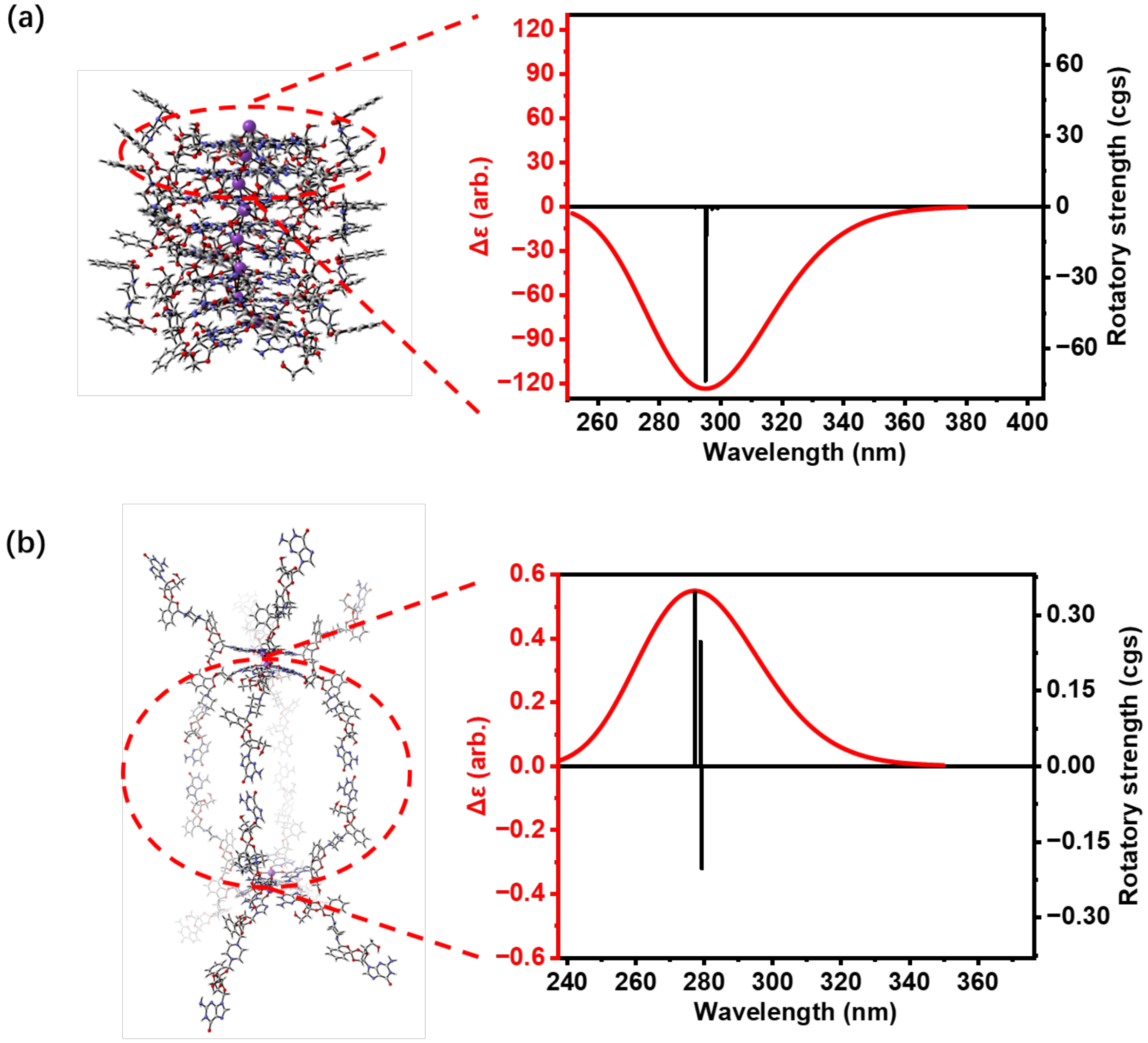

Supplementary Figure 23. The calculated ECD spectra for G-PBB hydrogels under (a) acid and (b) alkaline conditions, respectively. As there were a large number of atoms in the self-assembled system, we calculated the ECD spectra of the helically arranged motifs derived from the self-assembled G-quadruplex structures.

Supplementary Table 5. The quantity of added fuels for each cycle. Three samples were tested in parallel in each cycle.

	1st cycle		2nd cycle		3rd cycle		4th cycle		5th cycle		6th cycle	
	**PrS (μL)**	**KOH (mg)**	**PrS (μL)**	**KOH (mg)**	**PrS (μL)**	**KOH (mg)**	**PrS (μL)**	**KOH (mg)**	**PrS (μL)**	**KOH (mg)**	**PrS (μL)**	**KOH (mg)**
**Sample 1**	50	15	48	29	46	32	44	29	42	33	40	29
**Sample 2**	50	15	48	31	46	32	44	30	42	32	40	27
**Sample 3**	50	15	48	30	46	31	44	29	42	32	40	28

Note: the initial concentration of G was 1.0% w/v, the molar ratio of G and PBB was 2:1, and the total volume was 5.0 mL.

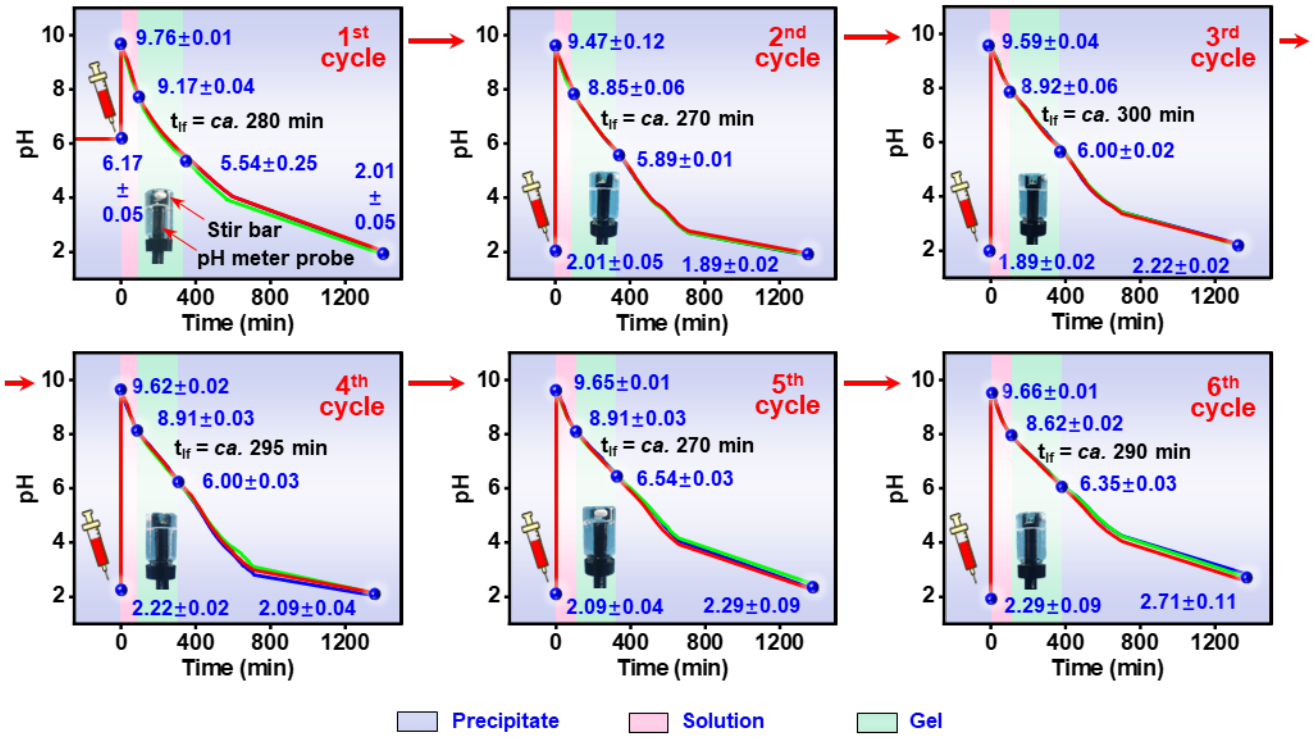

Supplementary Figure 24. pH-time profiles for six cycles of fuel addition. The insets show the experimental photographs of the transient hydrogels on inverting the vial during the cycles. In each cycle, three samples were tested in parallel.

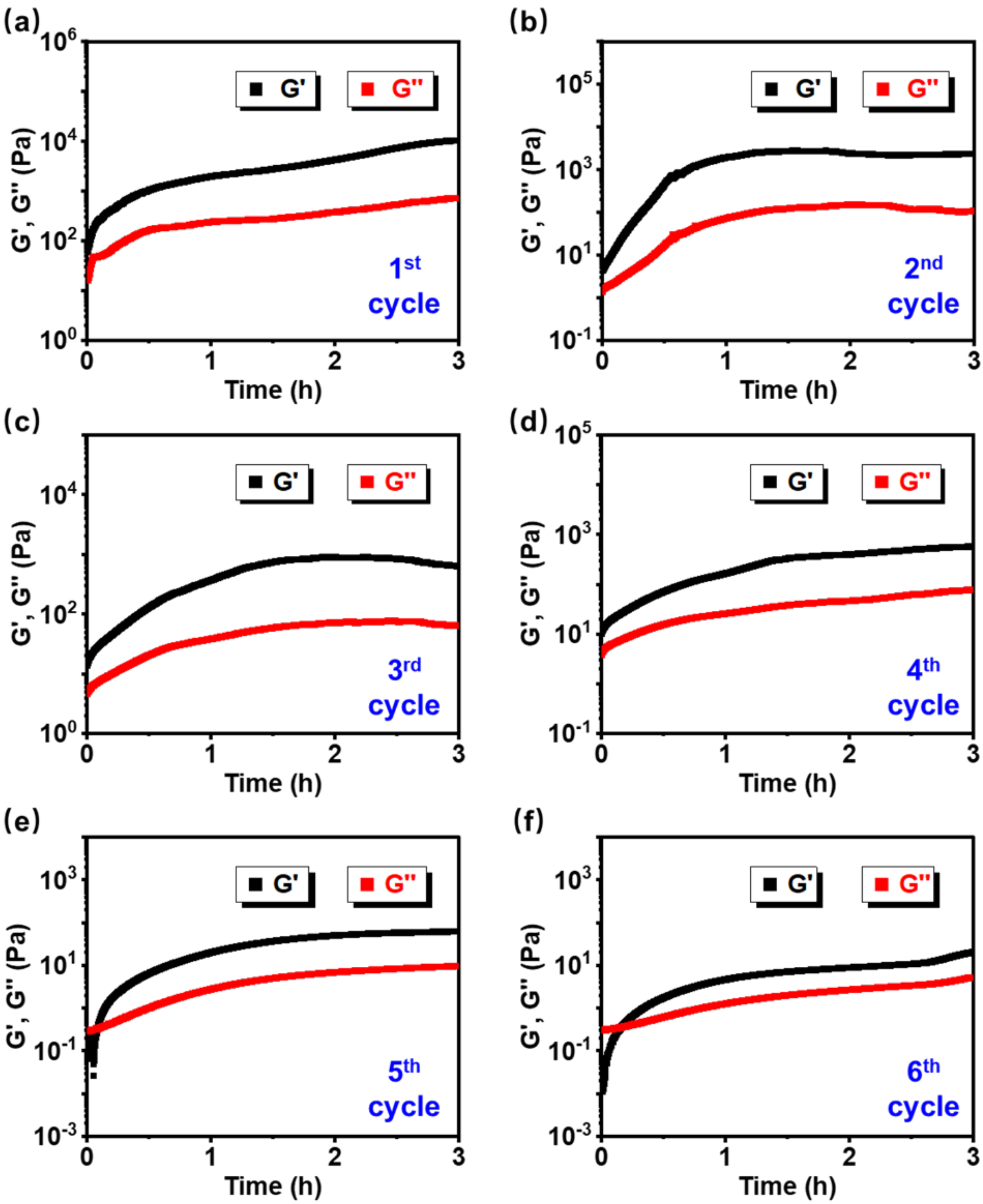

Supplementary Figure 25. Rheology time sweeps of the transient hydrogels for six repetitive cycles of the fuel addition. Pannels (a-f) are for the 1st, 2nd, 3rd, 4th, 5th and 6th cycles, respectively. The samples were prepared in separated containers, and the samples were placed between two plates for rheological tests immediately after refueling. The addition of fuels was performed every 12 hours.

Supplementary Table 6. Representative pH values and lifetime of the solution/gel state of the system with varying PrS concentration, related to Supplementary Figure 26.

m_{KOH} (mg)	V_{PrS} (μL)	pH values		Lifetime of gel state (min)
		Starting point of gel state	Starting point of phase separation state	
15	50	9.17	5.54	280
	75	9.02	5.61	162
	100	8.55	5.62	80
	300	8.03	5.41	35
	500	6.78	5.14	15

Note: the initial concentration of G was 1.0% w/v, the molar ratio of G and PBB was 2:1, and the total volume was 5.0 mL.

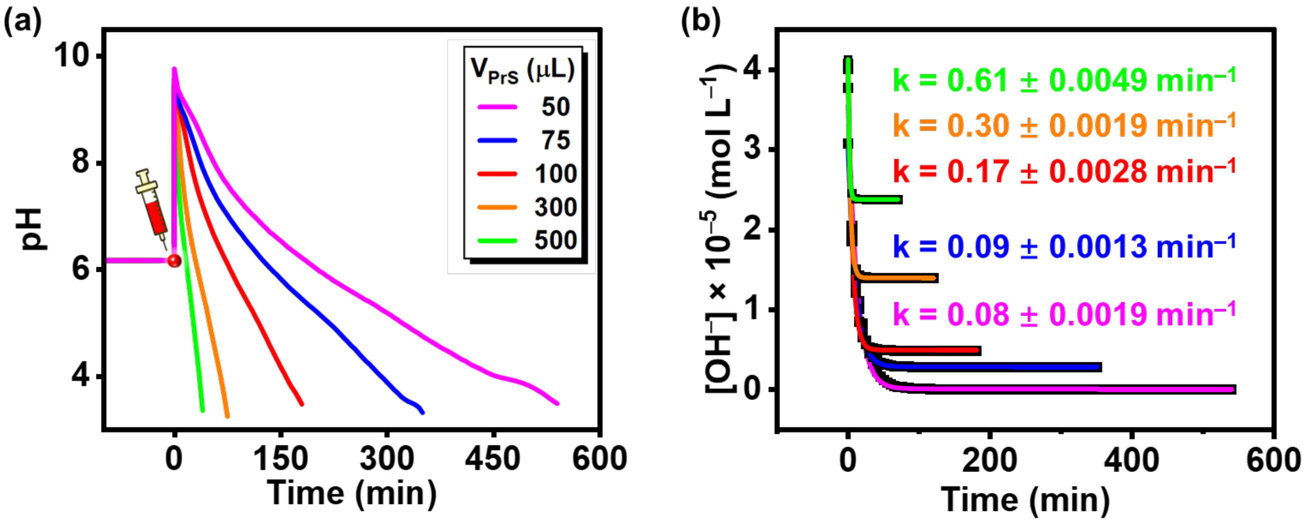

Supplementary Figure 26. (a) PH-time profiles of the G and PBB mixtures during one reaction cycle with varying PrS concentrations. In each case, three samples were tested in parallel, while only one group of data was shown to aviod poor visualization. (b) The concentration of OH^- against time in the hydrolysis of PrS (symbols). The measured pH is converted to $[OH^-]$. Exponential fits (lines) give the (pseudo) first order rate constants. Note, the y axis offset is applied for clarity.

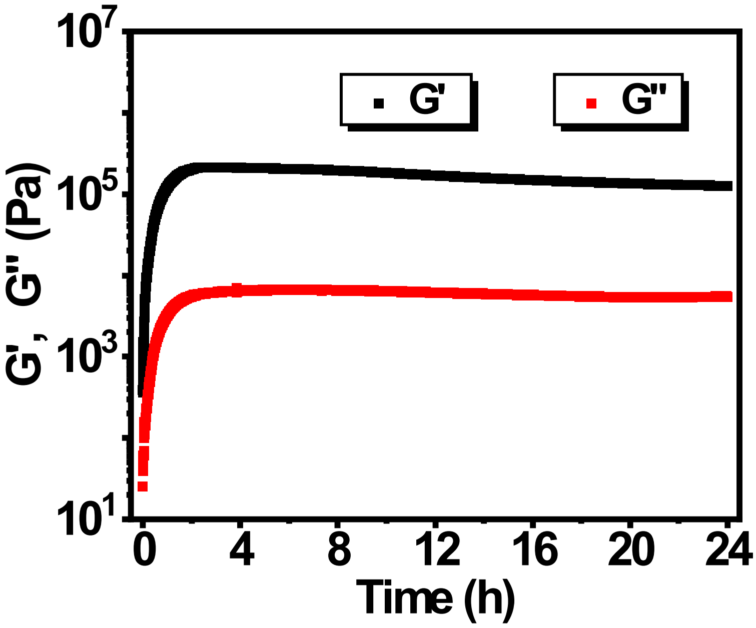

Supplementary Figure 27. Time-dependent rheological moduli profiles of the sample after the cycle initiation driven by KOH and methyl formate (MF).

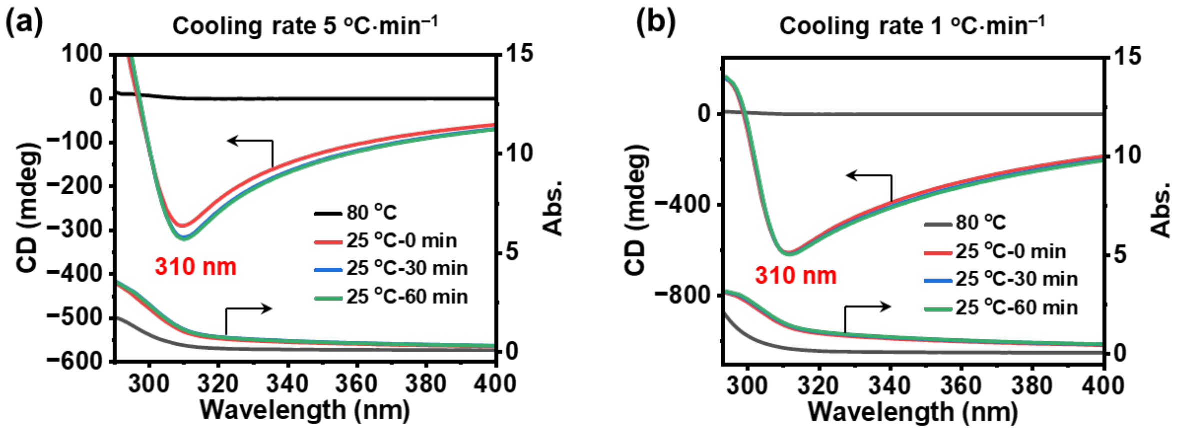

Supplementary Figure 28. CD and UV-vis spectra of the reconstituted gels prepared *via* further thermal annealing of the chemigels at different cooling rates. (a) 5 °C min^{-1} and (b) 1 °C min^{-1}. After cooling to 25 °C, the gels quickly reached thermodynamic equilibrium, and the spectral curves almost overlapped after different waiting time.

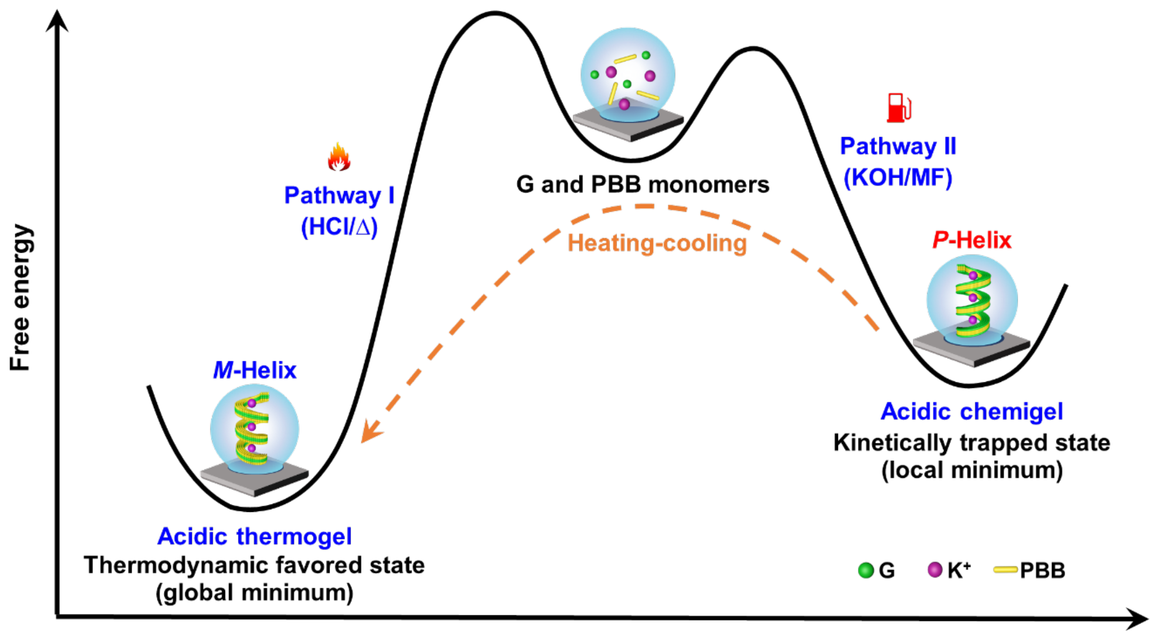

Supplementary Figure 29. Energy landscape for pathway dependent self-assembly of G and PBB mixture under acidic condition (pathway I: thermal-driven process; pathway II: chemical fuel-driven process).

Supplementary Table 7. Representative pH values and lifetime of the alkaline and acidic gels of the system with varying methyl formate (MF) concentration, related to Supplementary Figures 30 and 31.

KOH (aq. pH = 14) (μL)	V_{MF} (μL)	pH values		Lifetime of alkaline gel (min)	Lifetime of acidic gel
		Starting point of gel state	Final state		
350	30	9.57	7.26	1053	> 24 h
	50	9.54	6.71	130	> 24 h
	70	9.06	6.24	77	> 24 h
	100	8.73	5.86	24	> 24 h

Note: the initial concentration of G was 1.0% w/v, the molar ratio of G and PBB was 2:1, and the total volume was 5.0 mL.

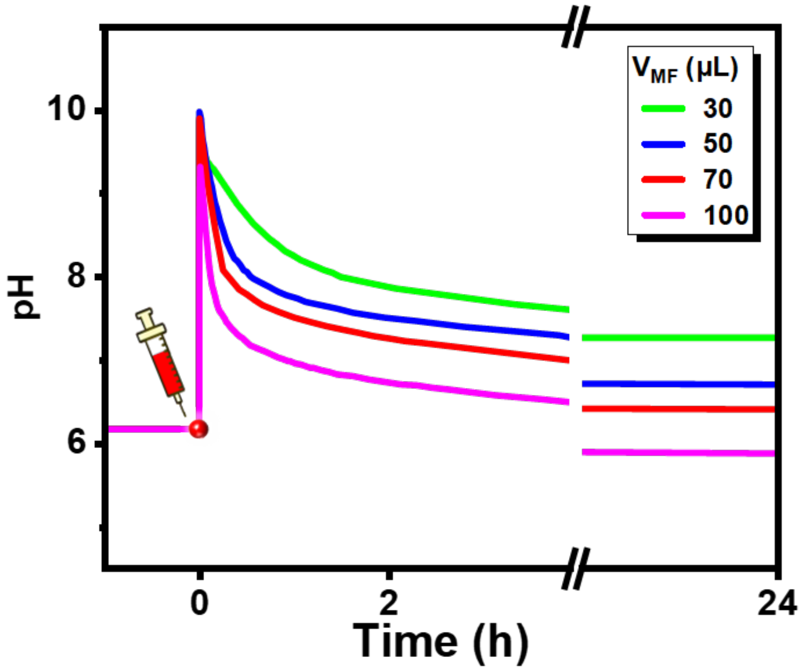

Supplementary Figure 30. Representative pH-time profiles of G and PBB mixture during one reaction cycle with varying methyl formate concentrations. In each case, three samples were tested in parallel, while only one group of data was shown to aviod poor visualization.

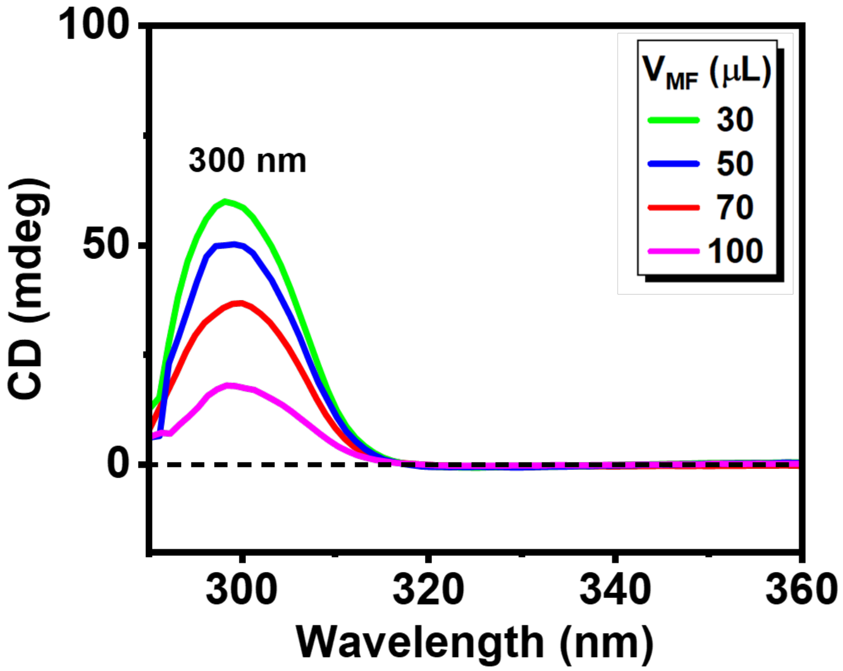

Supplementary Figure 31. CD spectra of the final obtained acidic chmeigels prepared *via* varying methyl formate concentrations during the reaction cycle.

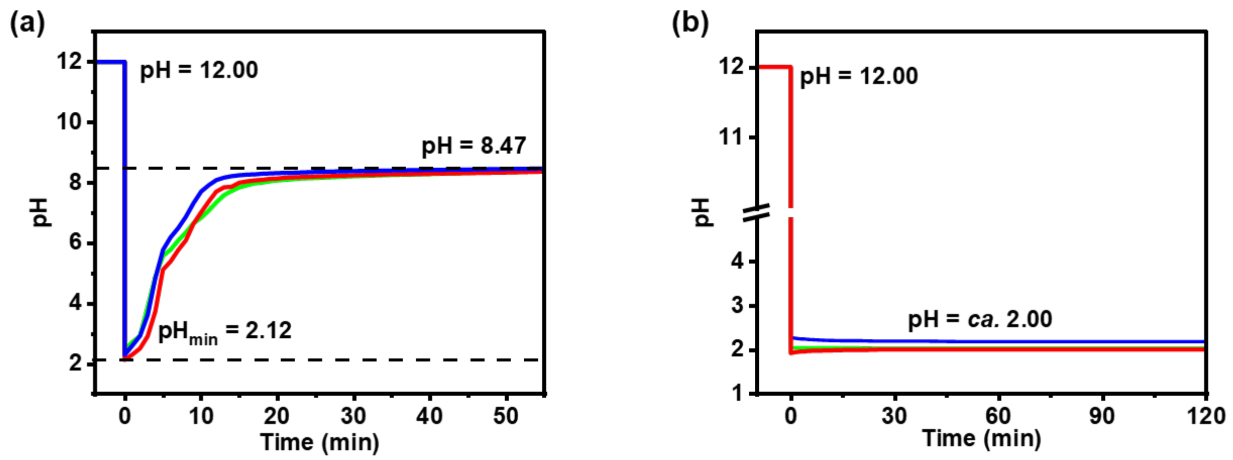

Supplementary Figure 32. (a) pH-time profiles of KOH and nitroacetic acid solution. 2.0 equiv. of nitroacetic acid was added to a solution of 0.010 M KOH (pH = 12.00) at time t = 0. (b) pH-time profiles of KOH and trichloroacetic acid solution. 2.0 equiv. of trichloroacetic acid was added to a solution of 0.010 M KOH (pH = 12.00) at time t = 0. In each case, three samples were tested in parallel.

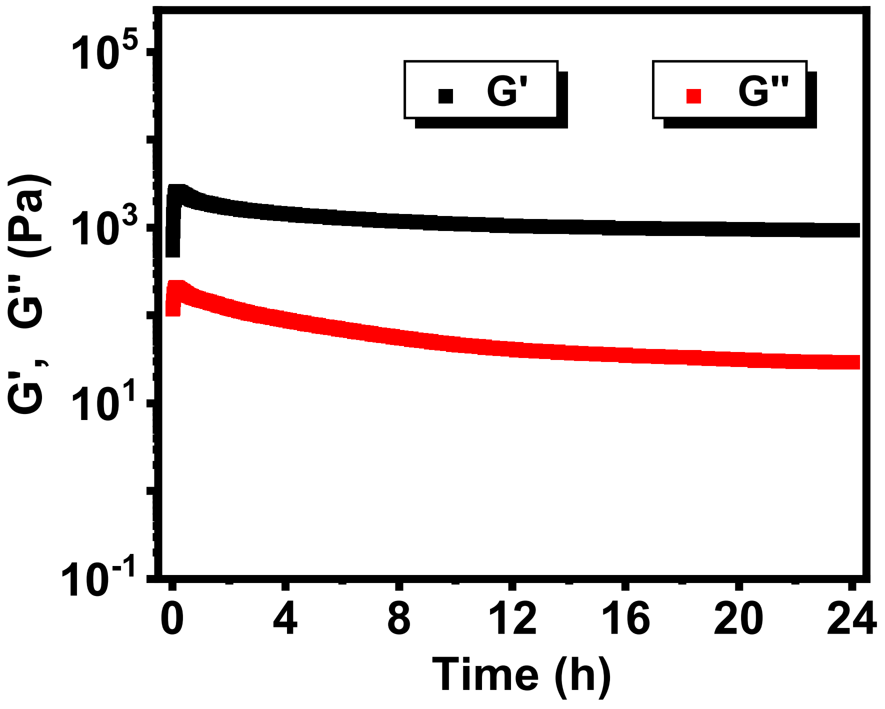

Supplementary Figure 33. Time-dependent rheological moduli profiles of the sample after cycle initiation driven by KOH and nitroacetic acid.

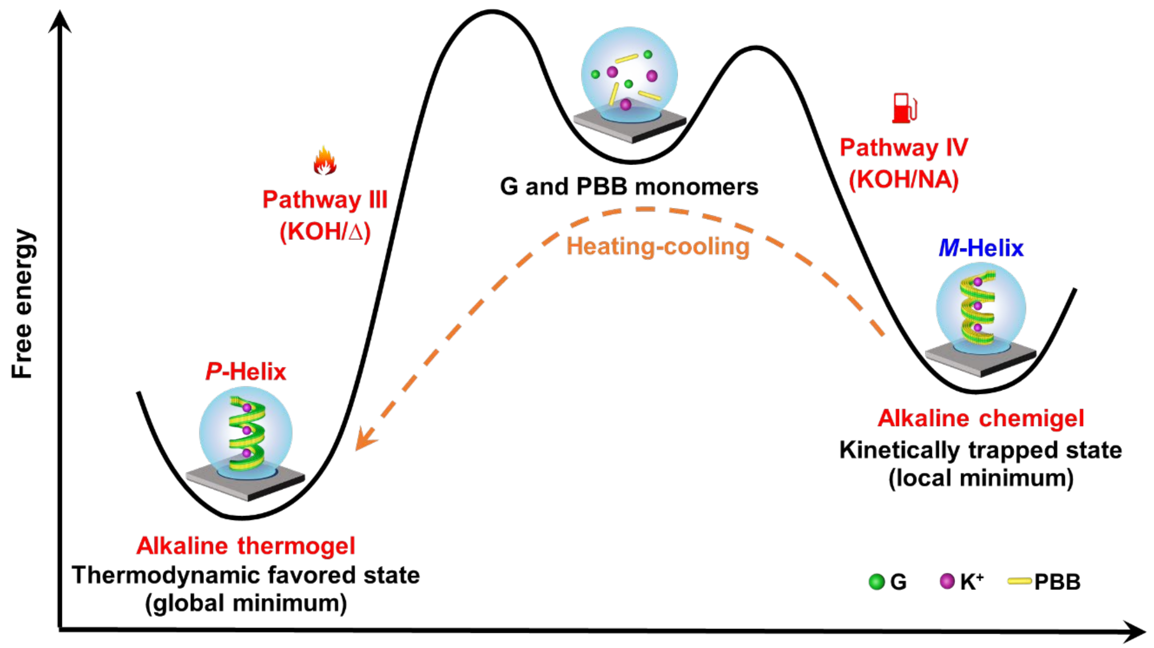

Supplementary Figure 34. Energy landscape for pathway dependent self-assembly of G and PBB mixture under alkaline condition (pathway III: thermal-driven process; pathway IV: chemical fuel-driven process).

Supplementary Table 8. Representative pH values and lifetime of the acidic and alkaline gels of the system with varying nitroacetic acid (NA) concentration, related to Supplementary Figures 35 and 36.

KOH (aq. pH = 14) (μL)	m_{NA} (mg)	pH values			Lifetime of acidic gel (min)	Lifetime of alkaline gel
		The minimum pH value after adding KOH and NA	Starting point of gel state	Final state		
140	12	7.22	7.38	8.54	53	> 24 h
	15	6.15	6.54	8.45	1352	> 24 h
	18	5.05	6.15	7.97	1725	> 24 h
	22	3.29	6.20	7.75	1544	> 24 h

Note: the initial concentration of G was 0.5% w/v, the molar ratio of G and PBB was 2:1, and the total volume was 4.0 mL and the sample thickness was *ca.* 1 cm.

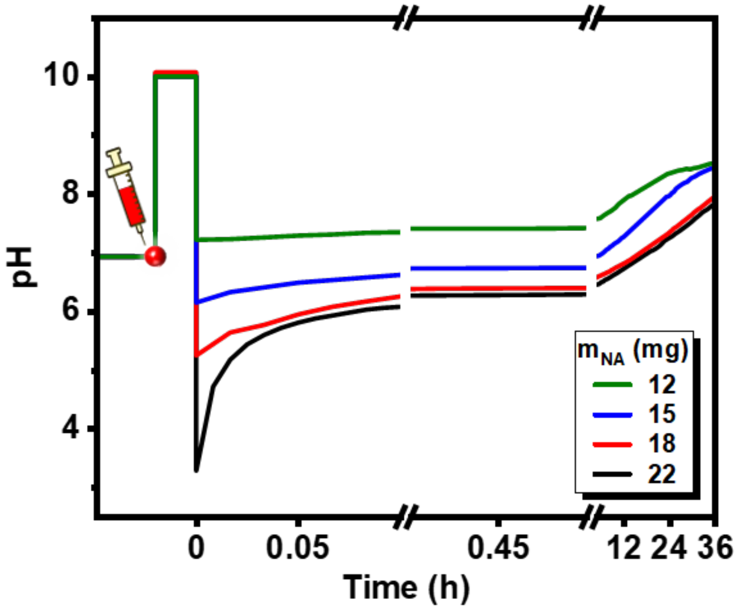

Supplementary Figure 35. Representative pH-time profiles of G and PBB mixture during one reaction cycle with varying nitroacetic acid concentrations. In each case, three samples were tested in parallel, while only one group of data was shown to aviod poor visualization.

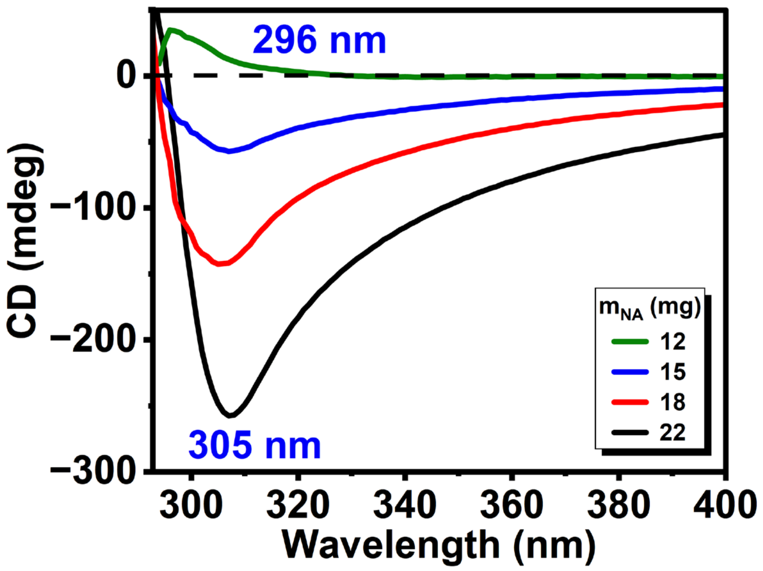

Supplementary Figure 36. CD spectra of the final obtained alkaline chemigels prepared *via* varying nitroacetic acid concentrations during the reaction cycle.

Supplementary Table 9. The quantity of added chemicals for each cycle for construction of multistate supramolecular chiroptical switches, related to Supplementary Figure 37.

	**Step 1 (Chemical process)**		**Step 3 (Chemical process)**	
	**KOH (aq. pH = 14) (μL)**	**Methyl formate (μL)**	**KOH (aq. pH = 14) (μL)**	**Nitroacetic acid (mg)**
**1st cycle**	60	28	120	12
**2nd cycle**	100	80	180	13
**3rd cycle**	120	100	200	14

Note: the initial alkaline thermogel (gel 1, pH = *ca.* 9.0) was prepared by conventically heating-cooling method in pH 12.5 aqueous KOH, the starting concentration of G was 0.7% w/v, the molar ratio of G and PBB was 2:1, and the total volume was 2.0 mL and the sample thickness was *ca.* 1 cm.

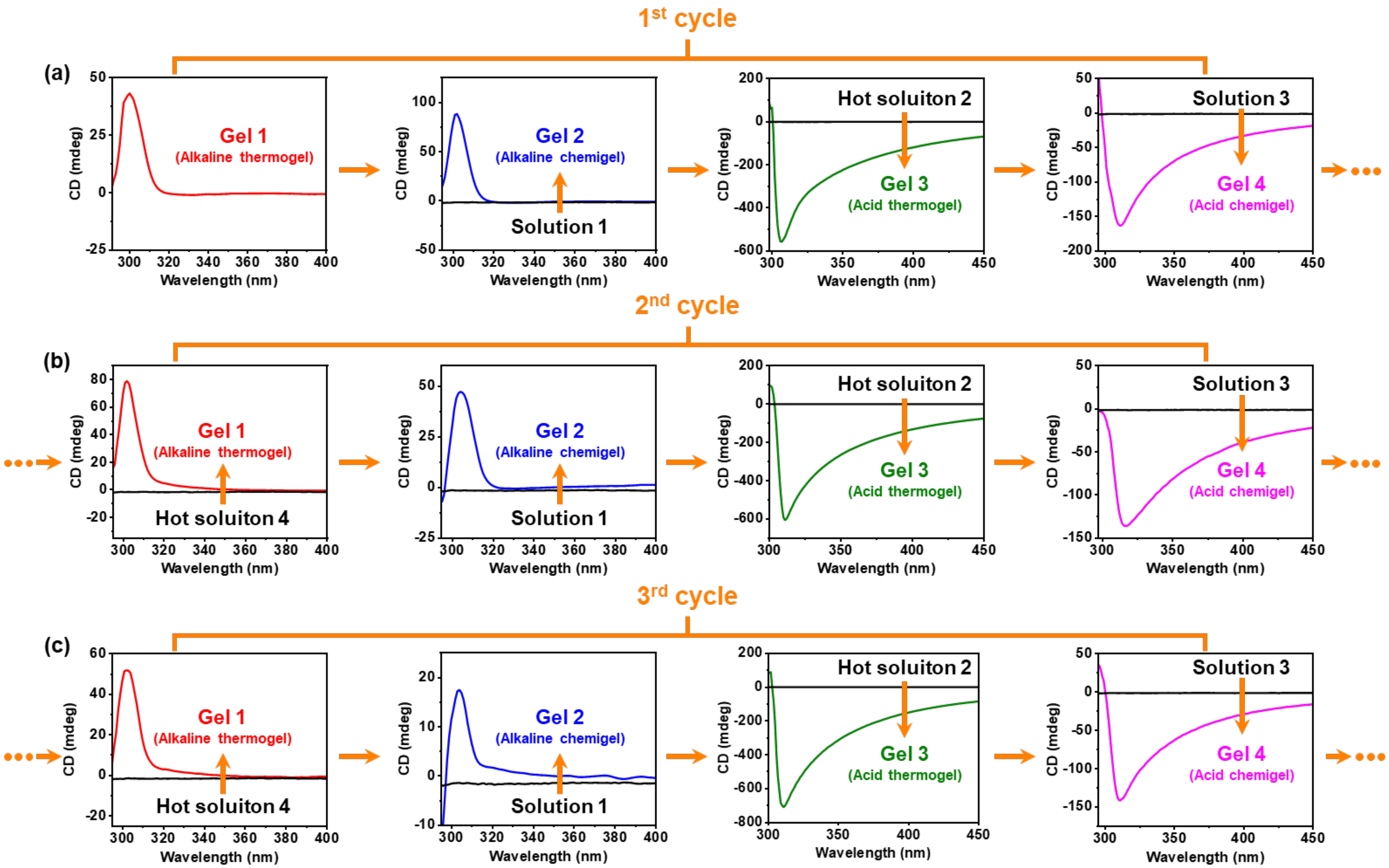

Supplementary Figure 37. Circular dichroism spectra of the switching samples in the (a) first, (b) second and (c) third full cycles.

Supplementary References

1. Adamczyk-Woźniak, A. et al. Straightforward synthesis and crystal structures of the 3-piperazine-bisbenzoxaboroles and their boronic acid analogs. *Tetrahedron* **69**, 8936–8942 (2013).
2. Qi, H., Li, X. & Xu, J. Stereoselective control in the Staudinger reactions involving monosubstituted ketenes with electron acceptor substituents: experimental investigation and theoretical rationalization. *Org. Biomol. Chem.* **9**, 2702–2714 (2011).
3. Vanier, S. F., Larouche, G., Wurz, R. P. & Charette, A. B. Formal Synthesis of Belactosin A and Hormaomycin via a Diastereoselective Intramolecular Cyclopropanation of an α-Nitro Diazoester. *Org. Lett* **12**, 672–675 (2010).
4. Zarzeczańska, D. et al. Fluorinated Boronic Acids: Acidity and Hydrolytic Stability of Fluorinated Phenylboronic Acids. *Eur. J. Inorg. Chem.* 4493–4498 (2017).
5. Bannwarth, C. et al. Extended tight-binding quantum chemistry methods. *WIREs Comput. Mol. Sci.* **11**, e1493 (2021).
6. Pracht, P., Caldeweyher, E., Ehlert, S. & Grimme, S. A Robust Non-Self-Consistent Tight-Binding Quantum Chemistry Method for large Molecules. *ChemRxiv* (2019), preprint. DOI: 10.26434/chemrxiv.8326202.v1.
7. Bannwarth, C., Ehlert, S. & Grimme, S. GFN2-xTB—An Accurate and Broadly Parametrized Self-Consistent Tight-Binding Quantum Chemical Method with Multipole Electrostatics and Density-Dependent Dispersion Contributions. *J. Chem. Theory Comput.* **15**, 1652–1671 (2019).
8. Becke, A. D. Density-functional thermochemistry. III. The role of exact exchange. *J. Chem. Phys.* **98**, 5648–5652 (1993).
9. Raghavachari, K. Perspective on "Density functional thermochemistry. III. The role of exact exchange". *Theor. Chem. Acc.* **103**, 361–363 (2000).
10. Becke, A. D. A new mixing of Hartree–Fock and local density-functional theories. *J. Chem. Phys.* **98**, 1372–1377 (1993).
11. Lee, C., Yang, W. & Parr, R. G. Development of the Colle-Salvetti correlation-energy formula into a functional of the electron density. *Phys. Rev. B* **37**, 785–789 (1988).
12. Schäfer, A., Horn, H. & Ahlrichs, R. Fully optimized contracted Gaussian basis sets for atoms Li to Kr. *J. Chem. Phys.* **97**, 2571–2577 (1992).
13. Weigend, F. & Ahlrichs, R. Balanced basis sets of split valence, triple zeta valence and quadruple zeta valence quality for H to Rn: Design and assessment of accuracy. *Phys. Chem. Chem. Phys .* **7**, 3297–3305 (2005).
14. Legault, C. Y. CYLview, 1.0b. Université de Sherbrooke, http://www.cylview.org. (2009).
15. Meng, L. et al. From G-quartets to G-ribbon gel by concentration and sonication control. *Org.*

Biomol. Chem. **11**, 1525–1532 (2013).

16. Venkatesh, V. et al. Supramolecular Photoactivatable Anticancer Hydrogels. *J. Am. Chem. Soc.* **139**, 5656–5659 (2017).

17. Ghosh, S., Li, X.-Q., Stepanenko, V. & Würthner, F. Control of H- and J-Type π Stacking by Peripheral Alkyl Chains and Self-Sorting Phenomena in Perylene Bisimide Homo- and Heteroaggregates. *Chem. Eur. J.* **14**, 11343–11357 (2008).

18. Xie, X.-Q. et al. Programmable Transient Supramolecular Chiral G-quadruplex Hydrogels by a Chemically Fueled Non-equilibrium Self-Assembly Strategy. *Angew. Chem. Int. Ed.* **61**, e202114471 (2022).
