# Supplementary material for: Programmable supramolecular chirality in non-equilibrium systems affording a multistate chiroptical switch
Source: Nat Commun. 2023 Aug 18;14:5030. doi: 10.1038/s41467-023-40698-9 (PMC10439165; doi:10.1038/s41467-023-40698-9)
Supplement: Supplementary file 3 — Description of Additional Supplementary Files [file 41467_2023_40698_MOESM3_ESM.pdf]

Description of Additional Supplementary Files

File Name: Supplementary Movie 1
Description: Simulated structural models of G and PBB mixture under both acidic and alkaline conditions.

File Name: Supplementary Movie 2
Description: Dynamic non-equilibrium self-assembly of G and PBB mixture with transient helicity *via* a fuel-driven chemical reaction network.

File Name: Supplementary Movie 3
Description: Kinetically trapped *P*-helical hydrogel driven by KOH and methyl formate (MF).

File Name: Supplementary Movie 4
Description: Kinetically trapped *M*-helical hydrogel driven by KOH and nitroacetic acid (NA).
