# Supplementary material for: Programmable supramolecular chirality in non-equilibrium systems affording a multistate chiroptical switch
Source: Nat Commun. 2023 Aug 18;14:5030. doi: 10.1038/s41467-023-40698-9 (PMC10439165; doi:10.1038/s41467-023-40698-9)
Supplement: Supplementary file 2 — Peer Review File [file 41467_2023_40698_MOESM2_ESM.pdf]

nature portfolio

Peer Review File

Programmable supramolecular chirality in non-equilibrium systems affording a multistate chiroptical switch

Open Access This file is licensed under a Creative Commons Attribution 4.0 International License, which permits use, sharing, adaptation, distribution and reproduction in any medium or format, as long as you give appropriate credit to the original author(s) and the source, provide a link to the Creative Commons license, and indicate if changes were made. In the cases where the authors are anonymous, such as is the case for the reports of anonymous peer reviewers, author attribution should be to 'Anonymous Referee' followed by a clear attribution to the source work. The images or other third party material in this file are included in the article's Creative Commons license, unless indicated otherwise in a credit line to the material. If material is not included in the article's Creative Commons license and your intended use is not permitted by statutory regulation or exceeds the permitted use, you will need to obtain permission directly from the copyright holder. To view a copy of this license, visit http://creativecommons.org/licenses/by/4.0/.

REVIEWER COMMENTS</B>

Reviewer #1 (Remarks to the Author):

The authors report a multistate chiroptical switch that can be used multiple times.
To achieve a multistate chiroptical switch, the authors couple an extended version of their previous in Angew. Chem.2022,134, e202114 reported chemically-fueled transient supramolecular chiral G-quadruplex hydrogels to a heating-cooling cycle, showing that they can switch pathways dependent on different chiralities.

On the novelty
The reported system is interesting for designing new supramolecular materials with programmable supramolecular chirality. However, the idea has been introduced previously. Chiroptical switches based on two or three types of switching phenomena exist. The novelty comes from developing three-step chiroptical switches based on a chemical-fueled reaction that is coupled with a heat-cooling cycle. The manuscript would become more powerful if the authors commented on for which applications their system could be used and on how generalizable the design of their multistate chirality switches is for other chiral assemblies, e.g. crystals.
Major
1) Please add a descriptive scheme on your developed switch system in the introduction.
2) You use AFM, TEM, and SEM to determine the morphology of your assemblies. For this, you dry your hydrogels, thereby converting them into xerogels. This heavily affects the morphology. For the alkaline gels, you mention that you do not observe helical features due to the xerogel formation. Cryo-TEM does not affect morphology. Thus, I suggest you do Cryo-TEM on your samples to confirm that your obtained morphologies do not result from drying effects. Tomography on your Cryo-TEM samples allows you to determine the chirality.
3) In your introduction, line 5, page 3, you state that currently developed supramolecular materials exist mainly in equilibrium. Later, you state that chemically fueled self-assemblies, e.g., hydrogels, have been recently developed. These two statements are contradicting, please clarify.
4) In line 11, page 6, you mention that control experiments show the crucial role of …. This seems crucial for understanding the manuscript. Thus, please explain in one sentence in your manuscript what the control experiments showed.
5) In lines 6-8, page 7, you state that you used different w/v of G at different pH`s. Please explain why and state in figure Caption 1B that you used different conditions.
6) Which role do the waste products play in your system? Does the accumulation of waste product poison your system at some point?
7) For which applications can your multistate chiroptical switch be used?
8) How generally applicable is the mechanism of sustaining chirality due to kinetic trapping for other systems, e.g. crystals? Please comment based on the literature.
9) In line 15, p7, you mention the internal agreement. Please clarify what this means.
10) In Figures 1g and 1k, you overlay multiple CD spectra to show the change of the CD spectra by increasing or decreasing the temperature. From these graphs, it is unclear which CD-spectra

corresponds to which temperature. Please show a maximum of three CD spectra per plot and label the corresponding temperature. E.g., for Figure 1g: 25°C, 45°C, and 70°C. Please do this also for Figure 3i, Figure 4 e, and 4i.
11) In Figure 2b and line 15, p9, you state that the intensity of the G-PBB-G diester increased relatively. I hardly see this from the NMR chromatograms. It would be much better if you would plot the two peaks' relative intensity as a function of temperature. Please do this also for Figure 2 e to show that the peak does not change.
12) In lines 7-9, p10, you state that a transitional aggregation state exists G-PBB-pHaq.1– 2 and G-PBB-pHaq.12–14. What does this mean? Do both species coassemble and form a new supramolecular structure, or do orthogonal self-assemblies of M and P helices exist at these pH values?
13) In line 25, p11, you state that you develop in situ regulation of the helical structures resembling those in living systems. Please give examples of regulation of the helical structures in living systems.
14) Self-assembled molecules typically have a reduced transversal relaxation time (T2), leading to a signal broadening and, thus, signal loss. Self-assembled molecules are thus NMR silent. Please clarify in your manuscript how you measured the NMR spectra of your hydrogels to avoid this effect so that you can rely on your NMR spectra.
15) Why does the G-PBB-G disassemble and form precipitates at very low pH and not convert into the M-helices? (lines 14-15, p 13).
16) Line 28-29, p11, Line 7-8, p14. Please express the amount of added KOH and PrS as concentrations or equivalents.
17) In lines 11-13, p 16, you state that the P-helices are kinetically trapped, even though the G-PBB-G diester is completely hydrolyzed. Please clarify why the helices remain and do not dissociate. In lines 14-15, p 13, you stated that the gel disassembles and precipitates at a very low pH. So, do you only have “kinetically trapped” P-helices in a certain pH window?
18) When you fuel your system with KOH and NA do you observe G-PBB-G diester formation?
19) Showing an energy landscape of your multistate chiroptical switch in the manuscript would be helpful.

Reviewer #2 (Remarks to the Author):

The self-assembly behaviors of the G/PBB gelator were studied under different temperature and pH values. The samples were well characterized and the results were well discussed. Especially, the molecular dynamic simulations gave us a better understanding the molecular packing structures. This manuscript is acceptable after minor revising.
Herein, the authors only show the first CD signal at the long wavelength. It is reasonable to know the supramolecular handedness based on the sign of the signal. The UV-vis and CD spectra can be taken simultaneously using the apparatus of CD. It is better to show the UV-vis and CD spectra at 200-280 nm. Then, the packing handedness of the aromatic rings can be revealed. Moreover, based on the molecular packing structure, the CD spectra can be calculated using TD-DFT. Namely, the simulated molecular packing structures can be partially confirmed by the calculated CD spectra.

Reviewer #3 (Remarks to the Author):

In this manuscript, the authors explored the fuels-triggered self-assembly behavior of guanosine (G) and a mono-benzoxaborole (MB) derivative, which performs pH-responsive supramolecular chirality. The authors conducted a series of experiments to study the assembly mechanism under non-equilibrium systems and demonstrated a potential application as a chiroptical switch. However, the property of pH-dependent chirality and related mechanism had already been reported in the authors' previous work "Programmable Transient Supramolecular Chiral G-quadruplex Hydrogels by a Chemically Fueled Non-equilibrium Self-Assembly Strategy" (Angew. Chem. Int. Ed. 2022, 61, e202114471), in which a similar assembly system had been investigated by using guanosine (G) and 5-fluorobenzoxaborole (B) in the presence of chemical fuels (KOH and 1,3-propanesultone). Compared with the authors' previous report, this manuscript does not make essential improvement on scientific novelty. Therefore, I'm afraid that this manuscript does not reach the high-quality requirement of Nature Communications.

Here are detailed suggestions:

1) In the section of "Kinetically trapped supramolecular chiral inversion", how did the molecular assemblies change when the acidic (alkaline) gel switched to the alkaline (acidic) gel. The authors should keep tracking the molecular assembly during the pH change by using IR, NMR, XRD, and UV spectrum to analyze the assembly mechanism, which will make a better understanding on the relationship between the molecular assemblies and the CD signals.
2) As shown in Figure 4a, the CD signal of the molecular assemblies keep positive or negative during the pH change. Generally, the chiral signal of the supramolecular aggregate would not change unless the assembly was affected or disturbed by environmental factors. In my opinion, the change of the chirality showed in Figure 4 is a common phenomenon. I suggest the authors reconsidering the concept of the chiral inversion.
3) In the section of "Chiroptical switching between the thermogels and the chemigels", the inversion of chiral signals can only be achieved by a heating-cooling process, which usually takes a long time to realize gelation. I suppose that thermal control is not a good choice for operating a chiroptical switch. I suggest that it is better to design a new demonstration to realize a fast and easily controlled switch between different states.

Prof. Dr. Chun-Sen Liu
Henan Provincial Key Laboratory of Surface & Interface Science
Zhengzhou 450002, Henan, People's Republic of China
E-mail: chunsenliu@zzuli.edu.cn Fax: ++86-371-86608700 (O)

Point-by-point response

To Reviewer 1:

1. Please add a descriptive scheme on your developed switch system in the introduction.

Thank you very much for reviewing our work. Your suggestions are very helpful for us. A descriptive scheme (Fig. R1 below) has been added in the introduction to illustrate the developed switch system. ***Please see: Page 4, Figure 1 in the main text.*** Accordingly, all figure numbers of the cross-indexes in the main text and Supplementary Information have been updated with this revision.

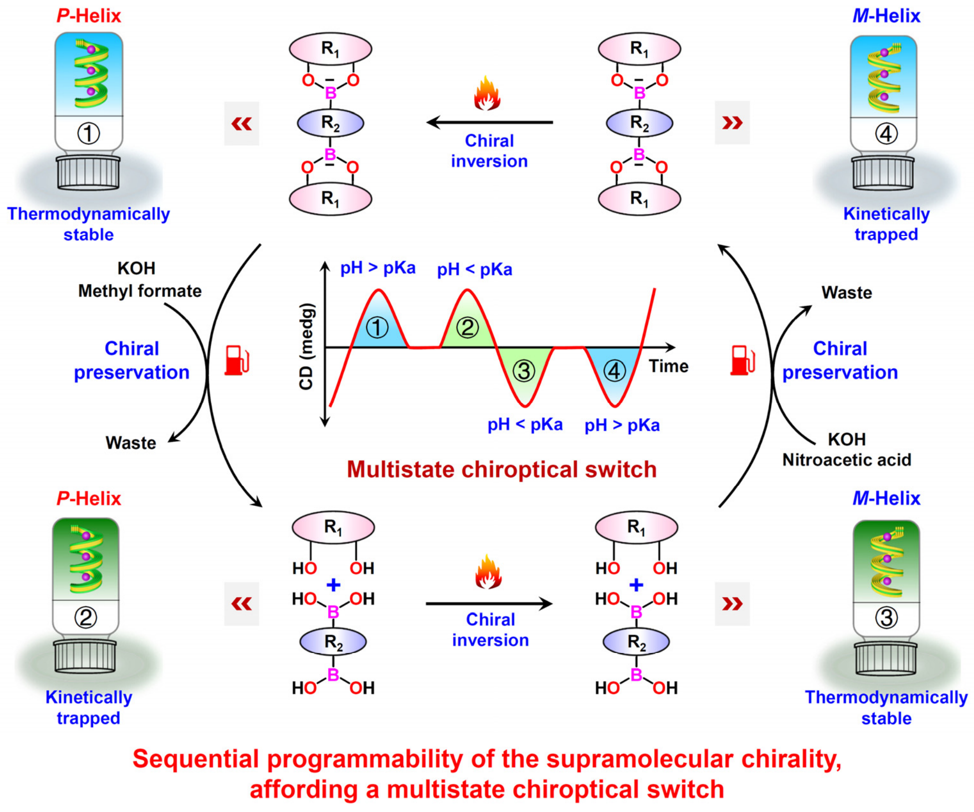

Fig. R1 Illustration of the design concept of a multistate supramolecular chiroptical switch via coupling of successive chemical-fueled process and thermal annealing process.

2. You use AFM, TEM, and SEM to determine the morphology of your assemblies. For this, you dry your hydrogels, thereby converting them into xerogels. This heavily affects the morphology. For the alkaline gels, you mention that you do not observe helical features due to the xerogel

Prof. Dr. Chun-Sen Liu
Henan Provincial Key Laboratory of Surface & Interface Science
Zhengzhou 450002, Henan, People's Republic of China
E-mail: chunsenliu@zzuli.edu.cn Fax: ++86-371-86608700 (O)

formation. Cryo-TEM does not affect morphology. Thus, I suggest you do Cryo-TEM on your samples to confirm that your obtained morphologies do not result from drying effects. Tomography on your Cryo-TEM samples allows you to determine the chirality.

Thank you very much for your comments and valuable suggestions. According to your suggestion, we have attempted to observe the assembly structure of G-PBB-$pH_{aq.1}$ and G-PBB-$pH_{aq.13}$ hydrogels using cryo-TEM (Fig. R2 below). It revealed that transparent G-PBB-$pH_{aq.13}$ hydrogel (2% w/v in G, 40 mg/mL) produced uniform fibers with a diameter of ~3 nm (Fig. R2a below). However, the fibers were too thin to discern their macromolecular helicity. For opaque G-PBB-$pH_{aq.1}$ hydrogel (4% w/v in G, 40 mg/mL), it was very difficult to prepare suitable samples for cryo-TEM observation due to its high concentration and rapid gelation speed. When the concentration is below 4%, it forms a precipitate. Therefore, it is not possible to dilute the hydrogel of G-PBB-$pH_{aq.1}$ for cryo-TEM sample preparation. However, we were delighted to discover that the helical structure of the G-PBB-$pH_{aq.1}$ hydrogel could be clearly seen using cryo-SEM (Fig. R2b). We have added this figure in the main text and included a few more sentences to discuss this point. ***Please see: Page 6, Figures 2c and d; Page 7, lines 6-12 in the revised manuscript, as well as Page S18, Supplementary Figure 9 in the SI file.***

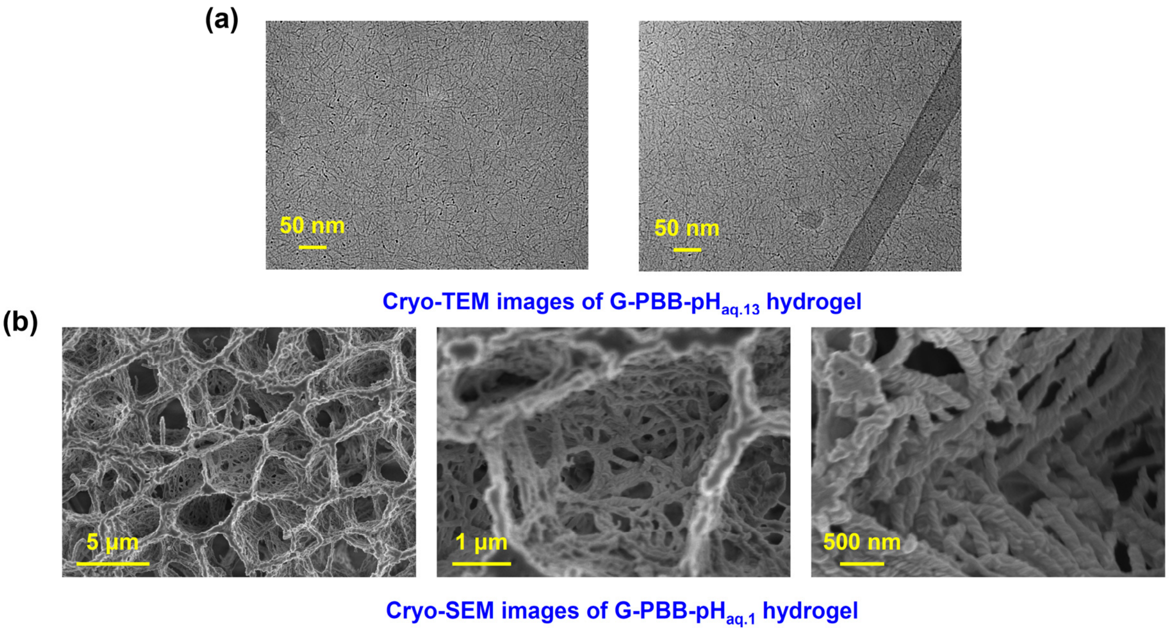

Figure R2. Cryo-TEM and Cryo-SEM images of the assembly structures of G-PBB-$pH_{aq.13}$ and G-PBB-$pH_{aq.1}$ hydrogels, respectively.

Prof. Dr. Chun-Sen Liu
Henan Provincial Key Laboratory of Surface & Interface Science
Zhengzhou 450002, Henan, People's Republic of China
E-mail: chunsenliu@zzuli.edu.cn Fax: ++86-371-86608700 (O)

***3.** In your introduction, line 5, page 3, you state that currently developed supramolecular materials exist mainly in equilibrium. Later, you state that chemically fueled self-assemblies, e.g., hydrogels, have been recently developed. These two statements are contradicting, please clarify.*

Thank you for bringing this to my attention. I apologize for any confusion caused. Indeed, there has been a lot of research on both equilibrium and non-equilibrium supramolecular materials. However, from an overall viewpoint, research on equilibrium supramolecular materials has a long history dating back to the 1960s, and remains a rapidly growing field (please refer to *Supramolecular Chemistry*, J.-M. Lehn (Ed.), New York: Wiley, **1995**). Supramolecular materials are primarily synthesized and applied in equilibrium systems across science and technology fields (please refer to *Supramolecular Nanotechnology*, O. Azza, M. Conda-Sheridan (Ed.), New York: Wiley, **2023**). Whereas research on artificially synthesized non-equilibrium supramolecular materials, such as hydrogels, began only in 2010 (please refer to *Angew. Chem. Int. Ed.* **2010**, *49*, 4825−4828; *Adv. Mater.* **2020,** *32*, e1906834). There is still much to be learned about these fascinating materials. Therefore, these two sentences are not contradictory, they just provide different perspectives on the current state of supramolecular materials.

***4.** In line 11, page 6, you mention that control experiments show the crucial role of …. This seems crucial for understanding the manuscript. Thus, please explain in one sentence in your manuscript what the control experiments showed.*

Sorry for missing this information. Control experiments showed that, without addition of guanosine, PBB or KOH or replacing KOH with LiOH, no hydrogels could be obtained in pH 1-14 aqueous. These findings highlight the significant role of G, PBB, and K^+ as well as their strong interactions for gelation. To further clarify these results, we have included an additional sentence in the main text. ***Please see: Page 7, lines 1-4 in the revised manuscript.***

***5.** In lines 6-8, page 7, you state that you used different w/v of G at different pH`s. Please explain why and state in figure Caption 1B that you used different conditions.*

Thank you for bringing up these issues. As shown in Supplementary Table 2, the critical gelator concentration (CGC) of G/PBB mixture in aqueous with different pH was different. Re-

Prof. Dr. Chun-Sen Liu
Henan Provincial Key Laboratory of Surface & Interface Science
Zhengzhou 450002, Henan, People's Republic of China
E-mail: chunsenliu@zzuli.edu.cn Fax: ++86-371-86608700 (O)

search on macroscopic gelation properties does not have special requirement for concentration. However, spectroscopy studies, especially CD studies, necessitated the use of the lowest possible concentrations to maintain absorbance levels below 2.0. To ensure consistency across all characterization data, experiments were carried out at their respective CGCs, resulting in different concentrations at different pH values. As suggested, we have highlighted this point in Figure caption 1B. ***Please see: Page 6, lines 9-10 in the revised manuscript.***

***6.** Which role do the waste products play in your system? Does the accumulation of waste product poison your system at some point?*

Yes, you are quite right. An important issue with synthetic dissipative systems is the accumulation of waste product, which will poison the system. As a result, most of the previously reported chemical reaction networks can only be recycled two or three times without removing the waste (please refer to *Science* **2015**, *349*, 1075–1079; *Angew. Chem. Int. Ed.* **2010**, *49*, 4825–4828; *Angew. Chem. Int. Ed.* **2019**, *58*, 244–247; *J. Am. Chem. Soc.* **2018**, *140*, 2869–2874). By using small molecules as chemical fuels (KOH and PrS), our system maintains the waste at a minimal level. As a result, the system can maintain a stable assembly cycle of at least six rounds.

7. For which applications can your multistate chiroptical switch be used?

By combining heating stimulus, chemical fuels stimulus, and acid-base indicators to afford color change, the proposed chiroptical switch has potential for creating chiral logic gates and for information storage and encryption (please refer to *Adv. Mater.* **2022**, *34*, 2109496; *Adv. Mater.* **2016**, *28*, 1644−1649, *et al.*). Additionally, our G-quadruplex hydrogels with multistate chiroptical switch can be used as excellent templates for circularly polarized luminescence (CPL) materials design and application. For example, these hydrogels can be co-assembled with fluorescent dye thioflavin T (ThT), a molecular chaperone for G-quartet, or bind with Eu(III) ions to produce CPL materials (please refer to *Chem* **2022**, 8, 1395−1414; *J. Mater. Chem. B* **2021**, 9, 7140−7144, *et al.*).

Prof. Dr. Chun-Sen Liu
Henan Provincial Key Laboratory of Surface & Interface Science
Zhengzhou 450002, Henan, People's Republic of China
E-mail: chunsenliu@zzuli.edu.cn Fax: ++86-371-86608700 (O)

8. How generally applicable is the mechanism of sustaining chirality due to kinetic trapping for other systems, e.g. crystals? Please comment based on the literature.

In our systems, the sustaining of the chirality throughout the consumption of chemical fuels allows for the creation of kinetic trapped chiral supramolecular materials. This approach presents a novel method for the synthesis of other chiral supramolecular materials, including liquid crystals and inorganic nanocrystals. Recently, there have been reports on the preparation of transient silicon nanocrystals (SiNCs) and kinetically trapped gold nanoparticles using chemical fuels (*Angew. Chem. Int. Ed.* **2018**, 57, 14608−14612; *J. Am. Chem. Soc.* **2019**, 141, 9872−9878). Unfortunately, these materials are not chiral. The development of chiral materials such as chiral crystals through the chemical fuel-driven kinetically trapped method could lead to distinct structures and properties resembling those found in living systems (*Nature* **2022**, 607, 287–293). Therefore, the mechanism of maintaining chirality through kinetic trapping driven by chemical fuels provides a general method for creating new chiral materials.

9. In line 15, p7, you mention the internal agreement. Please clarify what this means.

Sorry for confusing you. The original sentence was intended to convey that two different methods were used to measure molar enthalpy (ΔH), and that the values obtained from each method were found to be in good agreement with each other. As a result, the data is deemed to be self-consistent. To avoid any further confusion, we have deleted the related description.

10. In Figures 1g and 1k, you overlay multiple CD spectra to show the change of the CD spectra by increasing or decreasing the temperature. From these graphs, it is unclear which CD-spectra corresponds to which temperature. Please show a maximum of three CD spectra per plot and label the corresponding temperature. E.g., for Figure 1g: 25°C, 45°C, and 70°C. Please do this also for Figure 3i, Figure 4 e, and 4i.

Many thanks for your kind reminding. The CD spectra have been revised as suggested. The figure numbers have been revised due to the inclusion of a scheme as Figure 1. Therefore, ***please see: Page 6, Figure 2; Page 13, Figure 4 and Page 16, Figure 5 in the revised version.***

Prof. Dr. Chun-Sen Liu
Henan Provincial Key Laboratory of Surface & Interface Science
Zhengzhou 450002, Henan, People's Republic of China
E-mail: chunsenliu@zzuli.edu.cn Fax: ++86-371-86608700 (O)

11. In Figure 2b and line 15, p9, you state that the intensity of the G-PBB-G diester increased relatively. I hardly see this from the NMR chromatograms. It would be much better if you would plot the two peaks' relative intensity as a function of temperature. Please do this also for Figure 2 e to show that the peak does not change.

Many thanks for your valuable suggestions. The relative intensity of the two peaks ($I_{G\text{-}PBB\text{-}G}/I_{PBB}$) in Figure 3b as a function of temperature has been plotted (see Fig. R3a below). The normalized intensity of the single peak (I_{PBB}) in Figure 3e as a function of temperature has also been plotted (see Fig. R3b below). Both values increased with increasing temperatures due to the dissociation of the main components in the gel phase to the solution phase during melting of the hydrogels. We have added this figure in Supplementary Information. ***Please see: Page S22, Supplementary Figure 14 in the SI file.***

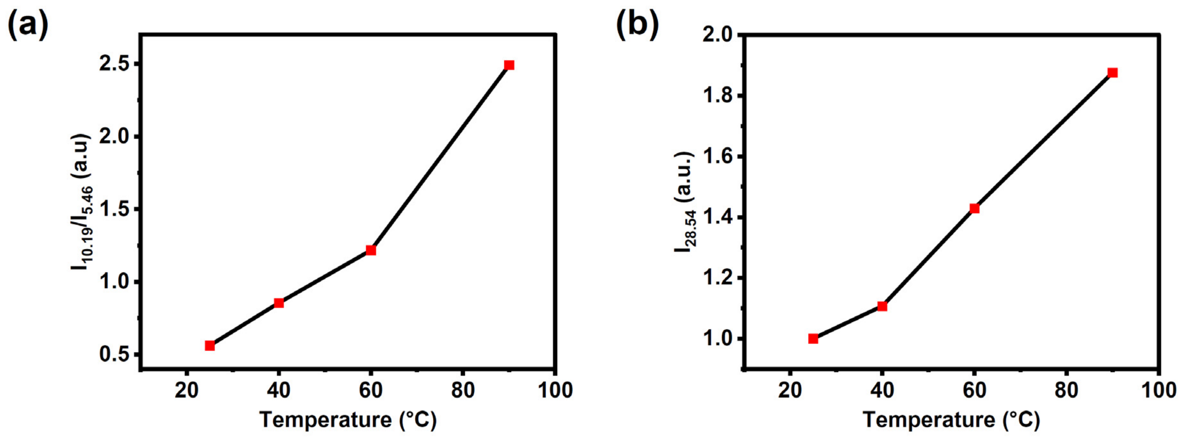

Figure R3. (a) The relative intensity of the two peaks ($I_{G\text{-}PBB\text{-}G/PBB}$) in Figure 3b in the main text as a function of temperature. (b) The normalized intensity of the single peak (I_{PBB}) in Figure 3e in the main text as a function of temperature.

12. In lines 7-9, p10, you state that a transitional aggregation state exists G-PBB-pHaq.1–2 and G-PBB-pHaq.12–14. What does this mean? Do both species coassemble and form a new supramolecular structure, or do orthogonal self-assemblies of M and P helices exist at these pH values?

Thank you for your insightful questions. As discussed in Figure 3 in the main text, cova-

lently bonded G-PBB-G diesters self-assembled to form alkaline G-PBB-pH$_{aq.13}$ hydrogel; however, non-covalently bonded G and PBB mixtures co-assembled to form the acidic G-PBB-pH$_{aq.1}$ hydrogel. Supplementary Figure 15 shows that the ^{11}B NMR spectra of G-PBB-pH$_{aq.12}$ and G-PBB-pH$_{aq.14}$ hydrogels had similar signals to those of G-PBB-pH$_{aq.13}$ hydrogel, showing coexistence of G-PBB-G diester and PBB^{2-} signals, indicating a similar gelation mechanism. The ^{11}B NMR spectra of G-PBB-pH$_{aq.2}$ hydrogel was similar to that of the G-PBB-pH$_{aq.1}$ hydrogel, displaying only one signal for neutral PBB signal, revealing their comparable self-assembly mechanism. However, the^{11}B NMR spectra of G-PBB-pH$_{aq.3-11}$ hydrogels exhibited both G-PBB-G diester and PBB signals. Hence, we infer that G-PBB-pH$_{aq.3-11}$ hydrogels represent an intermediate stage in the transition from G-PBB-pH$_{aq.1-2}$ hydrogels to G-PBB-pH$_{aq.12-14}$ hydrogels where both covalent and non-covalent bonding mechanisms contribute towards the self-assembly process. However, However, it is still unclear whether the two species co-assemble to form novel supramolecular structures or undergo orthodontic self-assembly. We have provided additional sentences to highlight this point and have removed the ambiguous description of transitional aggregation state. ***Please see: Page 11, lines 2-5 in the revised version.*** Once again, thank you for your thought-provoking inquiries.

13. *In line 25, p11, you state that you develop in situ regulation of the helical structures resembling those in living systems. Please give examples of regulation of the helical structures in living systems.*

Certainly, there are many examples of regulation of helical structures in living systems. For example, the bacterial flagellar filament, a helical propeller constructed from 11 protofilaments of a single protein, switches between left- and right-handed supercoiled forms when bacteria switch their swimming mode between running and tumbling (*Nature* **2001**, *410*, 331–337). Many viruses, such as the tobacco mosaic virus (TMV), have helical structures in their capsids. Once inside the host cell, TMV will disassembly the helical capsid structure, and release the genetic material for subsequent infection (*Sci. Rep.* **2018**, *8*, 11220). These are just a few examples of the regulation of helical structures in living systems. We have added these literatures in the main text. ***Please see: Page 23, refs.59 and 60 in the revised version.***

Prof. Dr. Chun-Sen Liu
Henan Provincial Key Laboratory of Surface & Interface Science
Zhengzhou 450002, Henan, People's Republic of China
E-mail: chunsenliu@zzuli.edu.cn Fax: ++86-371-86608700 (O)

***14.** Self-assembled molecules typically have a reduced transversal relaxation time (T2), leading to a signal broadening and, thus, signal loss. Self-assembled molecules are thus NMR silent. Please clarify in your manuscript how you measured the NMR spectra of your hydrogels to avoid this effect so that you can rely on your NMR spectra.*

Thank you for your comment. It is indeed correct that self-assembled molecules are NMR silent. In the case of hydrogels containing both solid fibers and solution, the NMR spectra of gels obtained at room temperature only reveal the molecules that dissolved in the entrapped solvent. However, as the temperature increases, the self-assembled molecules dissolve due to the melting of the gels, allowing them to be detected by NMR. Variable-temperature NMR spectra, as presented in Figure 3, can thus provide reliable information about the molecular structures in gel systems. We have added additional sentences in the manuscript to further clarify this point. ***Please see: Page 10, lines 15-18 in the revised version.***

***15.** Why does the G-PBB-G disassemble and form precipitates at very low pH and not convert into the M-helices? (lines 14-15, p 13).*

At very low pH, the stability of the G-quadruplex structure is decreased, leading to disassembly and precipitation of the hydrogels. The reason for this is likely due to changes in the hydrogen bonding and electrostatic interactions between the guanosine in the G-quadruplex structure at low pH, which disrupt the stabilizing interactions required for maintaining the quadruplex structure. Therefore, it is not likely to form an *M*-helix under the conditions where it disassembles and precipitates.

***16.** Line 28-29, p11, Line 7-8, p14. Please express the amount of added KOH and PrS as concentrations or equivalents.*

Thank you for your feedback. The amount of added KOH and PrS has been expressed as concentrations. ***Please see: Page 12, line 27 and Page 14, lines 2-3 in the revised version.***

***17.** In lines 11-13, p 16, you state that the P-helices are kinetically trapped, even though the G-PBB-G diester is completely hydrolyzed. Please clarify why the helices remain and do not dissociate. In lines 14-15, p 13, you stated that the gel disassembles and precipitates at a very low pH. So, do you only have "kinetically trapped" P-helices in a certain pH window?*

Prof. Dr. Chun-Sen Liu
Henan Provincial Key Laboratory of Surface & Interface Science
Zhengzhou 450002, Henan, People's Republic of China
E-mail: chunsenliu@zzuli.edu.cn Fax: ++86-371-86608700 (O)

Thank you for your insightful questions. Regarding the *P*-helices on lines 11-13 of page 16, they are stabilized by favorable interactions between the guanosine G-quadruplex backbone and the PBB molecules, which can provide enough energy to overcome any unfavorable interactions that might arise from the solvent molecules. However, the stability of the *P*-helices is not indefinite, as they can disassemble and precipitate at very low pH, as noted on lines 14-15 of page 13. Therefore, there is indeed a pH window within which the *P*-helices are kinetically trapped - this pH window is determined by the balance between favorable interactions between the guanosine backbone and the PBB molecules, and unfavorable interactions with solvent molecules. Outside of this pH window, the balance of stability shifts towards the unfavorable interactions and the *P*-helices dissociate. We have added additional sentences in the manuscript to discuss this point. ***Please see: Page 17, lines 12-16 in the revised version.***

18. *When you fuel your system with KOH and NA do you observe G-PBB-G diester formation?*

Yes, as can be seen from Fig. R4 below, time-dependent ^{11}B NMR spectra of the chemigel obtained in the final stage exhibited a distinct diester signal of G-PBB-G, firmly indicating the presence of the desired structure. We have added this figure in the main text and provided additional sentences in the manuscript to further discuss this point. ***Please see: Page 16, Figure 5h and Page 18, lines 10-13 in the revised version.***

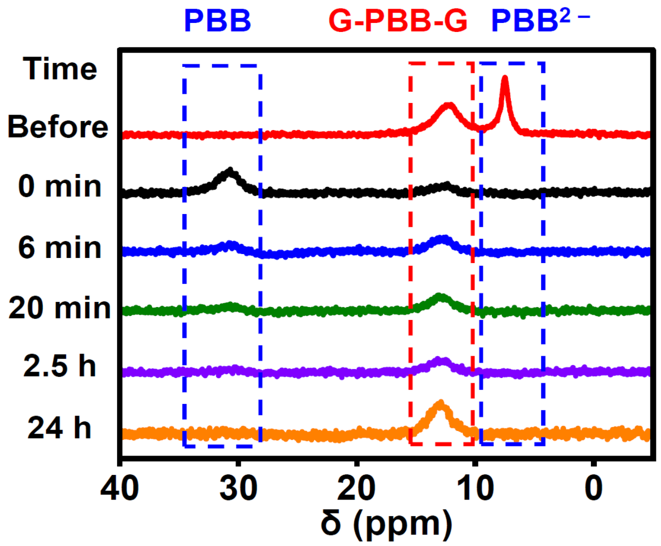

Figure R4. Time-dependent ^{11}B NMR spectra of the samples, showing structural evolution of the species after cycle initiation driven by KOH/nitroacetic acid.

Prof. Dr. Chun-Sen Liu
Henan Provincial Key Laboratory of Surface & Interface Science
Zhengzhou 450002, Henan, People's Republic of China
E-mail: chunsenliu@zzuli.edu.cn Fax: ++86-371-86608700 (O)

19. Showing an energy landscape of your multistate chiroptical switch in the manuscript would be helpful.

Thank you for your valuable suggestion. Based on our experimental data (Supplementary Table 3, Supplementary Figures 27 and 32), a possible energy landscape diagram of our multi-state chiroptical switch is demonstrated as below (Fig. R5). At present, we do not have experimental data that can verify the energy level differences between Gel 2 and Gel 4. Our speculation that the energy of Gel 4 is slightly lower than that of Gel 2 is based only on the observed energy difference between Gel 1 and Gel 3. Consequently, we decided not to include this hypothetical energy landscape diagram in the article due to the absence of definitive experimental evidence. Nevertheless, we appreciate your input and valuable suggestions for improving our article.

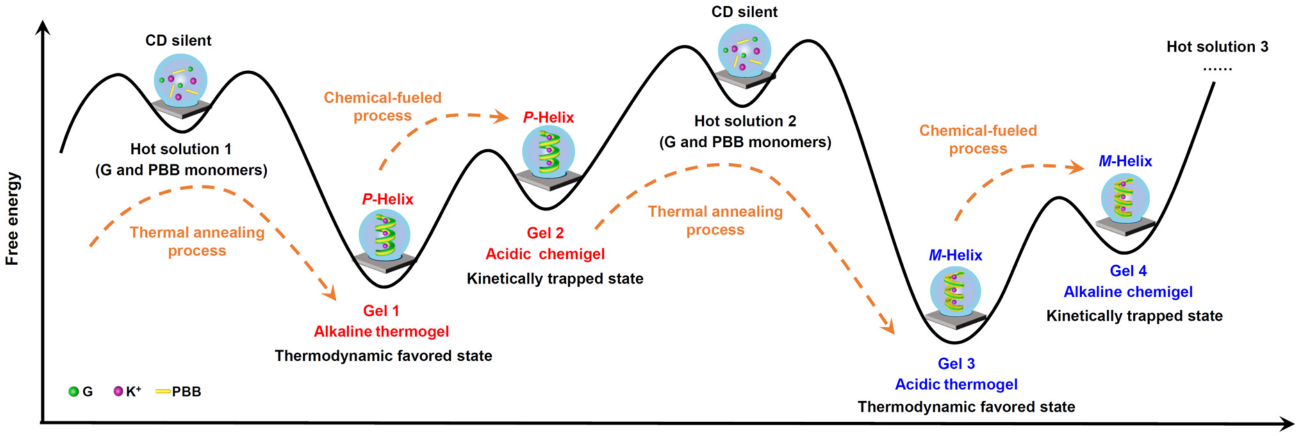

Figure R5. A possible energy landscape of our multistate chiroptical switch.

Many thanks once again for your valuable suggestions and careful reviewing of our paper.

Prof. Dr. Chun-Sen Liu
Henan Provincial Key Laboratory of Surface & Interface Science
Zhengzhou 450002, Henan, People's Republic of China
E-mail: chunsenliu@zzuli.edu.cn Fax: ++86-371-86608700 (O)

To Reviewer 2:

1.** The self-assembly behaviors of the G/PBB gelator were studied under different temperature and pH values. The samples were well characterized and the results were well discussed. Especially, the molecular dynamic simulations gave us a better understanding the molecular packing structures. **This manuscript is acceptable after minor revising.

Herein, the authors only show the first CD signal at the long wavelength. It is reasonable to know the supramolecular handedness based on the sign of the signal. The UV-vis and CD spectra can be taken simultaneously using the apparatus of CD. It is better to show the UV-vis and CD spectra at 200-280 nm. Then, the packing handedness of the aromatic rings can be revealed. Moreover, based on molecular packing structure, CD spectra can be calculated using TD-DFT. Namely, simulated molecular packing structures can be partially confirmed by calculated CD spectra.

Thank you for your positive feedback and valuable suggestions. Due to the high concentration of the hydrogels, the samples exhibit high UV absorption below 280 nm even using the thinnest cuvette (L = 0.1 mm), leading to overwhelming noise in the CD signals and making the resulting data unusable (see Fig. R6 below). Therefore, we only showed the UV-vis and CD spectra at the long wavelength (> 280 nm).

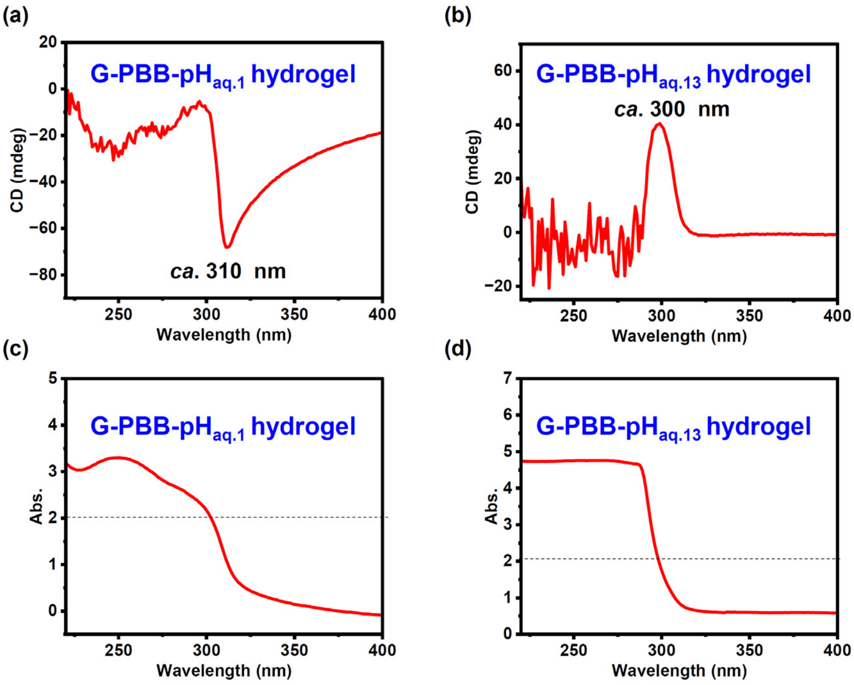

Figure R6. The CD (a, b) and corresponding UV-vis spectra (c, d) of G-PBB-pH$_{aq.1}$ and G-PBB-pH$_{aq.13}$ hydrogels.

Prof. Dr. Chun-Sen Liu
Henan Provincial Key Laboratory of Surface & Interface Science
Zhengzhou 450002, Henan, People's Republic of China
E-mail: chunsenliu@zzuli.edu.cn Fax: ++86-371-86608700 (O)

Additionally, we have performed TD-DFT calculations to confirm the simulated molecular packing structures as you suggested (Fig. R7 below). The results show that the theoretical CD spectra for helically arranged motifs derived from the self-assembled G-quadruplex structures are in good agreement with the experimental results. We have added this figure to the SI file and included a few more sentences to discuss this point in the main text. ***Please see: Page 12, lines 16-19 in the revised manuscript and Page S28, Supplementary Figure 21 in the SI file.***

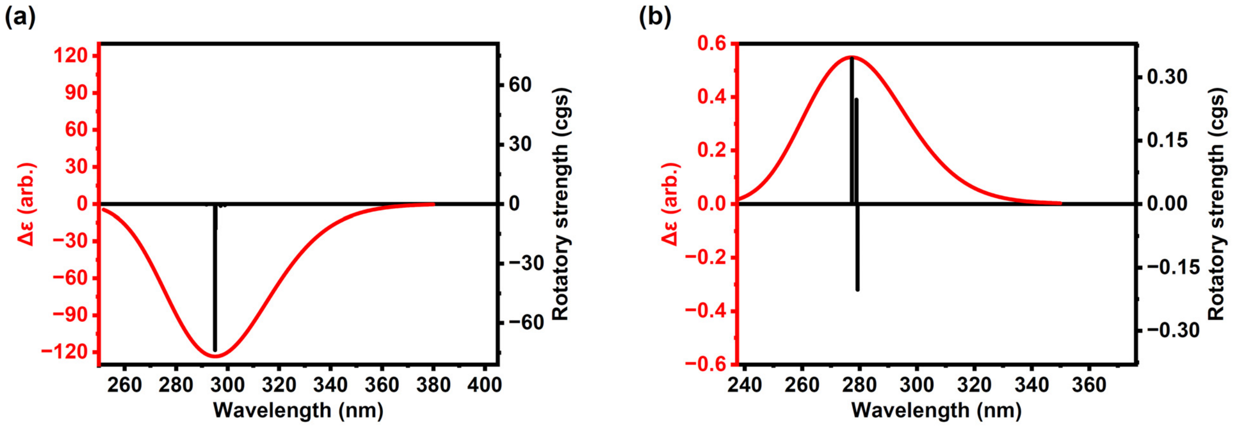

Figure R7. The calculated ECD spectra for G-PBB hydrogels under (a) acidic and (b) alkaline conditions, respectively.

Many thanks once again for your valuable suggestions and careful reviewing of our paper.

Prof. Dr. Chun-Sen Liu
Henan Provincial Key Laboratory of Surface & Interface Science
Zhengzhou 450002, Henan, People's Republic of China
E-mail: chunsenliu@zzuli.edu.cn Fax: ++86-371-86608700 (O)

To Reviewer 3:

***1.** In the section of "Kinetically trapped supramolecular chiral inversion", how did the molecular assemblies change when the acidic (alkaline) gel switched to the alkaline (acidic) gel. The authors should keep tracking the molecular assembly during the pH change by using IR, NMR, XRD, and UV spectrum to analyze the assembly mechanism, which will make a better understanding on the relationship between the molecular assemblies and the CD signals.*

Many thanks for your valuable suggestions. We have utilized NMR spectra (Fig. R8 below) to track the changes in the molecular assembly during pH changes. In KOH and methyl formate system, time-dependent ^{11}B NMR spectra have shown that the chemical structure evolved from G-PBB-G diesters to mixtures of G and PBB as the pH changed from alkaline to acidic (Fig. R8a below). In contrast, time-dependent ^{11}B NMR spectra in the KOH and nitroacetic acid (NA) system showed that during continuous pH reversal, the G-PBB-G diesters dissociated rapidly at first and then gradually reformed (Fig. R8b below). Given the high hydrogel concentrations, UV tracking was not possible; methods like IR and XRD would require the samples to be xerogels and, therefore, were also not suitable. As we have gained a firm understanding of the relationship between molecular assemblies and CD signals in the section of "Thermally triggered self-assembly and gelation (thermogels)," the NMR spectra presented in this section sufficiently illustrate the relationship between the molecular assemblies and the CD signals. We have added this figure in the main text and provided additional sentences in the manuscript to discuss these data. ***Please see: Page 15, lines 24-26; Page 16, Figures 5d and h, and Page 18, lines 10-13 in the revised version.***

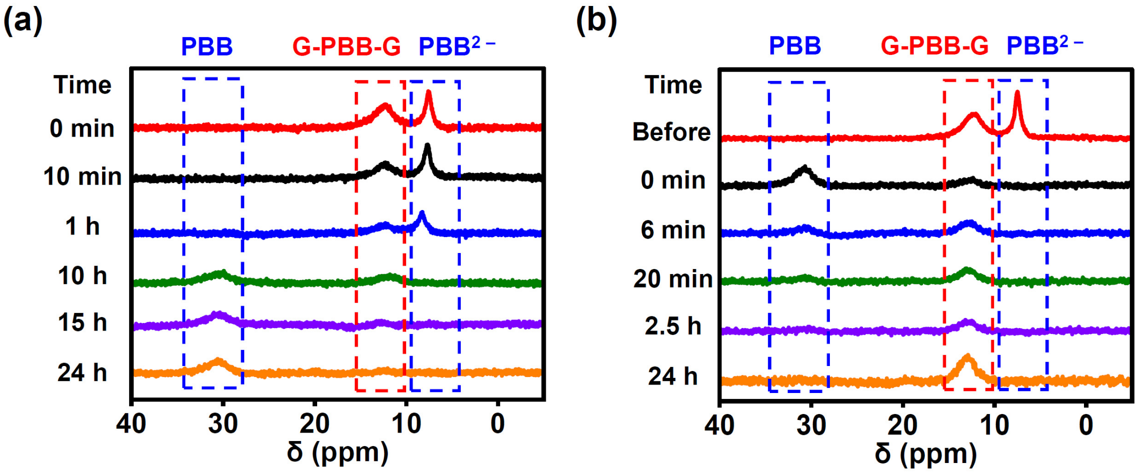

Figure R8. Time-dependent ^{11}B NMR spectra of the samples, showing structural evolution of the species after cycle initiation driven by (a) KOH/methyl formate and (b) KOH/nitroacetic acid.

2. As shown in Figure 4a, the CD signal of the molecular assemblies keep positive or negative during the pH change. Generally, the chiral signal of the supramolecular aggregate would not change unless the assembly was affected or disturbed by environmental factors. In my opinion, the change of the chirality showed in Figure 4 is a common phenomenon. I suggest the authors reconsidering the concept of the chiral inversion.

Many thanks for your comments. As you pointed out, the chiral signal of the supramolecular aggregate typically remains unchanged unless there are environmental disturbances affecting the assembly. In our study, we observed a transition from G-PBB-G diesters to mixtures of G and PBB when the alkaline gel switched to the acidic gel (and vice versa, as noted in your previous comment). Despite this transformation, the chiral signal was preserved, indicating that this is not a common phenomenon but rather a kinetically trapped result.

Moreover, by maintaining the same precursors and fuel alkalis while simply changing the type of fuel acids, the system displayed pathway-dependent non-equilibrium self-assembly, which led to either dynamic self-assembly with transient supramolecular chirality (Figure 4 in the main text) or kinetically trapped self-assembly with inverse supramolecular chirality (Figure 5 in the main text). This diverse regulation of supramolecular chirality has not been reported previously. The CD spectra in Figure 5 are not exact mirror images of each other, suggesting that the

(*P*)-supramolecular helicity obtained in the KOH/methyl formate (MF) system is diastereomerically related to the (*M*)-helicity observed in the KOH/nitroacetic acid (NA) system. Chiral inversion of this kind has been widely reported (please refer to *Adv. Mater.* **2020**, 32, 2004420; *J. Am. Chem. Soc.* **2022**, 144, 6019−6027; *Angew. Chem., Int. Ed.* **2016**, 55, 1001−1004. *Science* **2004,** *304*, 278−281. *et al*). Therefore, we believe that the concept of chiral inversion shown in Figure 5 is acceptable (Notably, Figure 4 in the origin manuscript has been changed as figure 5 in the revised version).

3. *In the section of "Chiroptical switching between the thermogels and the chemigels", the inversion of chiral signals can only be achieved by a heating-cooling process, which usually takes a long time to realize gelation. I suppose that thermal control is not a good choice for operating a chiroptical switch. I suggest that it is better to design a new demonstration to realize a fast and easily controlled switch between different states.*

Many thanks for your comments and valuable suggestions. In fact, similar to a photo stimulus, temperature is also a non-invasive stimulus that can be rapidly applied and removed. The heating and cooling rates are more readily adjustable, providing enhanced control over the gelation process. As a result, it has been widely utilized in chiroptical switchable systems (Please refer to *Chem. Soc. Rev.* **2020,** *49*, 9095−9120, section 6: *Temperature driven chiroptical switches; J. Am. Chem. Soc.* **2014***, 136,* 7972−7980; *J. Am. Chem. Soc.* **2022**, 144, 6019−6027; *Adv. Mater.* **2020**, 32, 2004420; *Science* **2004,** *304*, 278−281. *et. al*). In our system, gelation can be achieved within just 11 minutes using a cooling rate of 5 °C min^{-1} (please see Supplementary Figure 26), and the gelation time can be shortened further by adjusting the cooling rate. Moreover, by directly cooling the hot solution in ice water, the sample immediately undergoes gelation. Hence, thermal control is an efficient method for constructing chiroptical switches.

Many thanks once again for your valuable suggestions and careful reviewing of our paper.

REVIEWER COMMENTS

Reviewer #1 (Remarks to the Author):

In general, the reviewers respond by stating that other studies have seen similar behavior. But they don't cite that literature or make changes to the manuscript.

Please change the following points:

Reviewer 1. point 6:
Which role do the waste products play in your system? Does the accumulation of waste product poison your system at some point?
Authors: Yes, you are quite right. An important issue with synthetic dissipative systems is the accu- mulation of waste product, which will poison the system. As a result, most of the previously re- ported chemical reaction networks can only be recycled two or three times without removing the waste (please refer to Science 2015, 349, 1075–1079; Angew. Chem. Int. Ed. 2010, 49, 4825–4828; Angew. Chem. Int. Ed. 2019, 58, 244–247; J. Am. Chem. Soc. 2018, 140, 2869–2874). By using small molecules as chemical fuels (KOH and PrS), our system maintains the waste at a minimal level. As a result, the system can maintain a stable assembly cycle of at least six rounds.

These works should then be cited and a brief comment on the accumulation of waste should be added.

The same holds for point 7

Reviewer 1: 7. For which applications can your multistate chiroptical switch be used?

Authors: By combining heating stimulus, chemical fuels stimulus, and acid-base indicators to afford color change, the proposed chiroptical switch has potential for creating chiral logic gates and for information storage and encryption (please refer to Adv. Mater. 2022, 34, 2109496; Adv. Mater. 2016, 28, 1644−1649, et al.). Additionally, our G-quadruplex hydrogels with multistate chiropti- cal switch can be used as excellent templates for circularly polarized luminescence (CPL) materi- als design and application. For example, these hydrogels can be co-assembled with fluorescent dye thioflavin T (ThT), a molecular chaperone for G-quartet, or bind with Eu(III) ions to produce CPL materials (please refer to Chem 2022, 8, 1395−1414; J. Mater. Chem. B 2021, 9, 7140−7144, et al.).

These works should then be cited and a brief comment on the applicability should be added.

Reviewer 1: 8. How generally applicable is the mechanism of sustaining chirality due to kinetic trapping for other systems, e.g. crystals? Please comment based on the literature.

Authors: In our systems, the sustaining of the chirality throughout the consumption of chemical fuels

allows for the creation of kinetic trapped chiral supramolecular materials. This approach presents a novel method for the synthesis of other chiral supramolecular materials, including liquid crys- tals and inorganic nanocrystals. Recently, there have been reports on the preparation of transient silicon nanocrystals (SiNCs) and kinetically trapped gold nanoparticles using chemical fuels (Angew. Chem. Int. Ed. 2018, 57, 14608−14612; J. Am. Chem. Soc. 2019, 141, 9872−9878). Un- fortunately, these materials are not chiral. The development of chiral materials such as chiral crystals through the chemical fuel-driven kinetically trapped method could lead to distinct struc- tures and properties resembling those found in living systems (Nature 2022, 607, 287–293). Therefore, the mechanism of maintaining chirality through kinetic trapping driven by chemical fuels provides a general method for creating new chiral materials.
Great! Please add a comment to the main text and cite these works. I would also like to add: https://chemistry-europe.onlinelibrary.wiley.com/doi/10.1002/syst.202200035

In the response to reviewer 3, there is the same issues. Comments are dismissed by referring to the literature, but not updating the manuscript accordingly:

Reviewer 3. 2. As shown in Figure 4a, the CD signal of the molecular assemblies keep positive or negative during the pH change. Generally, the chiral signal of the supramolecular aggregate would not change unless the assembly was affected or disturbed by environmental factors. In my opinion, the change of the chirality showed in Figure 4 is a common phenomenon. I suggest the authors reconsidering the concept of the chiral inversion.

Authors: ...(P)-supramolecular helicity obtained in the KOH/methyl formate (MF) system is diastereomeri- cally related to the (M)-helicity observed in the KOH/nitroacetic acid (NA) system. Chiral inver- sion of this kind has been widely reported (please refer to Adv. Mater. 2020, 32, 2004420; J. Am. Chem. Soc. 2022, 144, 6019−6027; Angew. Chem., Int. Ed. 2016, 55, 1001−1004. Science 2004, 304, 278−281. et al). Therefore, we believe that the concept of chiral inversion shown in Figure 5 is acceptable (Notably, Figure 4 in the origin manuscript has been changed as figure 5 in the re- vised version).
Please cite these works and change the main text.

Reviewer 3. 3 In the section of “Chiroptical switching between the thermogels and the chemigels”, the inver- sion of chiral signals can only be achieved by a heating-cooling process, which usually takes a long time to realize gelation. I suppose that thermal control is not a good choice for operating a chiroptical switch. I suggest that it is better to design a new demonstration to realize a fast and easily controlled switch between different states.

Athors: Many thanks for your comments and valuable suggestions. In fact, similar to a photo stimu- lus, temperature is also a non-invasive stimulus that can be rapidly applied and removed. The heating and cooling rates are more readily adjustable, providing enhanced control over the gela- tion process. As a result, it has been widely utilized in chiroptical switchable systems (Please re- fer to Chem. Soc. Rev.

2020, 49, 9095−9120, section 6: Temperature driven chiroptical switches; J. Am. Chem. Soc. 2014, 136, 7972−7980; J. Am. Chem. Soc. 2022, 144, 6019−6027; Adv. Mater. 2020, 32, 2004420; Science 2004, 304, 278−281. et. al). In our system, gelation can be achieved within just 11 minutes using a cooling rate of 5 °C min-1 (please see Supplementary Figure 26), and the gelation time can be shortened further by adjusting the cooling rate. Moreover, by direct- ly cooling the hot solution in ice water, the sample immediately undergoes gelation. Hence, thermal control is an efficient method for constructing chiroptical switches.

Please cite these works and update the main text with a discussion.

Reviewer #2 (Remarks to the Author):

Since the molecular packing structures were confirmed. This manuscript is suitable to be published in NC.

Point-by-point response

To Reviewer 1:

1. Reviewer 1. point 6: *Which role do the waste products play in your system? Does the accumulation of waste product poison your system at some point?*

Authors: *Yes, you are quite right. An important issue with synthetic dissipative systems is the accumulation of waste product, which will poison the system. As a result, most of the previously reported chemical reaction networks can only be recycled two or three times without removing the waste (please refer to Science 2015, 349, 1075–1079; Angew. Chem. Int. Ed. 2010, 49, 4825–4828; Angew. Chem. Int. Ed. 2019, 58, 244–247; J. Am. Chem. Soc. 2018, 140, 2869–2874). By using small molecules as chemical fuels (KOH and PrS), our system maintains the waste at a minimal level. As a result, the system can maintain a stable assembly cycle of at least six rounds.*

These works should then be cited and a brief comment on the accumulation of waste should be added.

Many thanks for your valuable suggestions and careful reviewing on your paper again. We have added a brief comment on the accumulation of waste and cited the above mentioned literatures in the revised manuscript. ***Please see: Page 14, lines 14-17; Page 22, ref. 44 and Page 23, refs. 61-63 in the revised version.***

2. Reviewer 1: point 7. *For which applications can your multistate chiroptical switch be used?*

Authors: *By combining heating stimulus, chemical fuels stimulus, and acid-base indicators to afford color change, the proposed chiroptical switch has potential for creating chiral logic gates and for information storage and encryption (please refer to Adv. Mater. 2022, 34, 2109496; Adv. Mater. 2016, 28, 1644−1649, et al.). Additionally, our G-quadruplex hydrogels with multistate chiroptical switch can be used as excellent templates for circularly polarized luminescence (CPL) materials design and application. For example, these hydrogels can be co-assembled with fluorescent dye thioflavin T (ThT), a molecular chaperone for G-quartet, or bind with Eu(III) ions to*

produce CPL materials (please refer to Chem 2022, 8, 1395−1414; J. Mater. Chem. B 2021, 9, 7140−7144, et al.).

These works should then be cited and a brief comment on the applicability should be added.

As suggested, we have added a brief comment on the applicability and cited the above mentioned literatures in the revised manuscript. ***Please see: Page 20, lines 14-16; Page 22, ref. 28 and Page 24, refs. 76-78 in the revised version.***

3. Reviewer 1: point 8*. How generally applicable is the mechanism of sustaining chirality due to kinetic trapping for other systems, e.g. crystals? Please comment based on the literature.*

Authors: *In our systems, the sustaining of the chirality throughout the consumption of chemical fuels allows for the creation of kinetic trapped chiral supramolecular materials. This approach presents a novel method for the synthesis of other chiral supramolecular materials, including liquid crystals and inorganic nanocrystals. Recently, there have been reports on the preparation of transient silicon nanocrystals (SiNCs) and kinetically trapped gold nanoparticles using chemical fuels (Angew. Chem. Int. Ed. 2018, 57, 14608−14612; J. Am. Chem. Soc. 2019, 141, 9872−9878). Unfortunately, these materials are not chiral. The development of chiral materials such as chiral crystals through the chemical fuel-driven kinetically trapped method could lead to distinct structures and properties resembling those found in living systems (Nature 2022, 607, 287–293). Therefore, the mechanism of maintaining chirality through kinetic trapping driven by chemical fuels provides a general method for creating new chiral materials.*

Great! Please add a comment to the main text and cite these works. I would also like to add: https://chemistry-europe.onlinelibrary.wiley.com/doi/10.1002/syst.202200035

Many thanks for your positive comments and insightful input. We have added a comment on the generally applicability of the kinetic trapping for other systems and cited all above mentioned literatures in the revised manuscript. ***Please see: Page 18, lines 22-27; Page 23, ref. 47, and Page 24, refs. 72-74 in the revised version.***

4. Reviewer 3. point 2. *As shown in Figure 4a, the CD signal of the molecular assemblies keep positive or negative during the pH change. Generally, the chiral signal of the supramolecular*

aggregate would not change unless the assembly was affected or disturbed by environmental factors. In my opinion, the change of the chirality showed in Figure 4 is a common phenomenon. I suggest the authors reconsidering the concept of the chiral inversion.

Authors: *...(P)-supramolecular helicity obtained in the KOH/methyl formate (MF) system is diastereomerically related to the (M)-helicity observed in the KOH/nitroacetic acid (NA) system. Chiral inversion of this kind has been widely reported (please refer to Adv. Mater. 2020, 32, 2004420; J. Am. Chem. Soc. 2022, 144, 6019−6027; Angew. Chem., Int. Ed. 2016, 55, 1001−1004. Science 2004, 304, 278−281. et al). Therefore, we believe that the concept of chiral inversion shown in Figure 5 is acceptable (Notably, Figure 4 in the origin manuscript has been changed as figure 5 in the re- vised version).*

Please cite these works and change the main text.

Thank you very much for your valuable suggestions. We have cited these works and changed the main text. ***Please see: Page 18, lines 19-21; Page 21, ref. 8, and Page 24, refs. 69-71 in the revised version.***

5. Reviewer 3. point 3. *In the section of "Chiroptical switching between the thermogels and the chemigels", the inversion of chiral signals can only be achieved by a heating-cooling process, which usually takes a long time to realize gelation. I suppose that thermal control is not a good choice for operating a chiroptical switch. I suggest that it is better to design a new demonstration to realize a fast and easily controlled switch between different states.*

Authors: *Many thanks for your comments and valuable suggestions. In fact, similar to a photo stimulus, temperature is also a non-invasive stimulus that can be rapidly applied and removed. The heating and cooling rates are more readily adjustable, providing enhanced control over the gelation process. As a result, it has been widely utilized in chiroptical switchable systems (Please refer to Chem. Soc. Rev. 2020, 49, 9095−9120, section 6: Temperature driven chiroptical switches; J. Am. Chem. Soc. 2014, 136, 7972−7980; J. Am. Chem. Soc. 2022, 144, 6019−6027; Adv. Mater. 2020, 32, 2004420; Science 2004, 304, 278−281. et. al). In our system, gelation can be achieved within just 11 minutes using a cooling rate of 5 °C min*$^{-1}$ *(please see Supplementary Figure 26), and the gelation time can be shortened further by adjusting the cooling rate. Moreover, by directly cooling the hot solution in ice water, the sample immediately undergoes gelation.*

Hence, thermal control is an efficient method for constructing chiroptical switches.

Please cite these works and update the main text with a discussion.

Many thanks for your kind suggestions. We have cited these works and updated the main text with a discussion. ***Please see: Page 20, lines 11-13; Page 21, refs. 7-8, and Page 24, refs. 69, 71, 75 in the revised version.***

Many thanks once again for your valuable suggestions and careful reviewing of our paper.

To Reviewer 2:

No further revision should be done.

Many thanks once again for Reviewer 2’s positive comments and careful reviewing on our paper.
